# Sonolysis of per- and poly fluoroalkyl substances (PFAS): A *meta*-analysis

**DOI:** 10.1016/j.ultsonch.2022.105944

**Published:** 2022-02-07

**Authors:** Tim Sidnell, Richard James Wood, Jake Hurst, Judy Lee, Madeleine J. Bussemaker

**Affiliations:** a Department of Chemical and Process Engineering, University of Surrey, Guildford, Surrey GU2 7XH, United Kingdom; b Institute of Biomedical Engineering, Department of Engineering Science, University of Oxford, Oxford, United Kingdom; c ARCADIS, 1 Whitehall Riverside, Leeds LS1 4BN, United Kingdom

**Keywords:** PFAS, Sonolysis, Literature review, Meta-analysis, Parametric, Ultrasonic degradation

## Abstract

Human ingestion of per- and polyfluoroalkyl substances (PFAS) from contaminated food and water is linked to the development of several cancers, birth defects and other illnesses. The complete mineralisation of aqueous PFAS by ultrasound (sonolysis) into harmless inorganics has been demonstrated in many studies. However, the range and interconnected nature of reaction parameters (frequency, power, temperature etc.), and variety of reaction metrics used, limits understanding of degradation mechanisms and parametric trends. This work summarises the state-of-the-art for PFAS sonolysis, considering reaction mechanisms, kinetics, intermediates, products, rate limiting steps, reactant and product measurement techniques, and effects of co-contaminants. The *meta*-analysis showed that mid-high frequency (100 – 1,000 kHz) sonolysis mechanisms are similar, regardless of reaction conditions, while the low frequency (20 – 100 kHz) mechanisms are specific to oxidative species added, less well understood, and generally slower than mid-high frequency mechanisms. Arguments suggest that PFAS degradation occurs via adsorption (not absorption) at the bubble interface, followed by headgroup cleavage. Further mechanistic steps toward mineralisation remain to be proven. For the first time, complete stoichiometric reaction equations are derived for perfluorooctanoic acid (PFOA) and perfluorooctane sulfonic acid (PFOS) sonolysis, which add H_2_ as a reaction product and consider CO an intermediate. Fluorinated intermediate products are derived for common, and more novel PFAS, and a naming system proposed for novel perfluoroether carboxylates. The *meta*-analysis also revealed the transition between pseudo first and zero order PFOA/S kinetics commonly occurs at 15 – 40 µM. Optimum values of; ultrasonic frequency (300 – 500 kHz), concentration (>15 – 40 μM), temperature (≈20 °C), and pH range (3.2 – 4) for rapid PFOX degradation are derived by evaluation of prior works, while optimum values for the dilution factor applied to PFAS containing firefighting foams and applied power require further work. Rate limiting steps are debated and F^−^ is shown to be rate enhancing, while SO_4_^2−^ and CO_2_ by products are theorised to be rate limiting. Sonolysis was compared to other PFAS destructive technologies and shown to be the only treatment which fully mineralises PFAS, degrades different PFAS in order of decreasing hydrophobicity, is parametrically well studied, and has low-moderate energy requirements (several kWh g^−1^ PFAS). It is concluded that sonolysis of PFAS in environmental samples would be well incorporated within a treatment train for improved efficiency.

## Highlights

• PFAS sonolysis parameter *meta*-analysis.
• Optimum pH, frequency and temperature derived for PFOX sonolysis.
• Degradation occurs via adsorption bubble and headgroup removal.
• PFOA/S sonolysis produces only CO_2_, H_2_, H^+^, and F^−^
• Oxidative agents alter degradation mechanism.

## Graphical abstract

**Figure:**
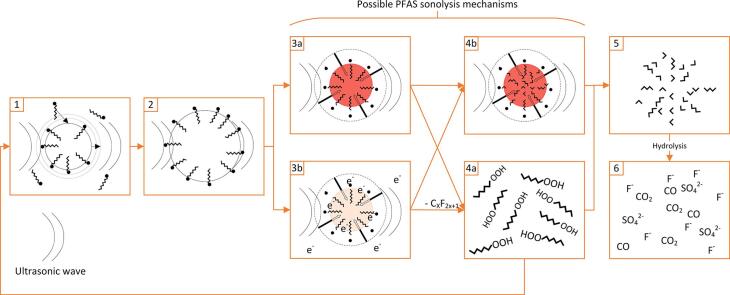

## 1 Introduction – PFAS concerns, definitions, and research to date

Per- and polyfluoroalkyl substances (PFAS) have been manufactured since the 1940s but, since the 1990s, have come under increasing scrutiny [1], [2] due to their detrimental impacts on human, animal and environmental health [3], [4]. PFAS enter aqueous matrices from; use and disposal of various consumer products and aqueous firefighting foams (AFFFs) [5], industrial pollution [6], landfills [7], [8] and in situ transformation from larger precursor molecules [9], [10]. Most water bodies contain at least a few pg L^−1^ of the two formerly most used PFAS; perfluorooctanoic acid (PFOA) and perfluorooctane sulfonic acid (PFOS) [11]. PFAS are even detected in air at several pg m^−3^ [12]. Despite low environmental concentrations, PFAS concentrate in living tissue due to bio-accumulation [13], contributing to hormonal imbalances, organ dysfunction and development of several cancers [14], [15], among other diseases. As such, PFOS, PFOA and related compounds are deemed persistent organic pollutants under the Stockholm convention (added in 2009 and 2019, respectively) and only permitted in applications without alternatives [16], [17].

**Fig. 1:**
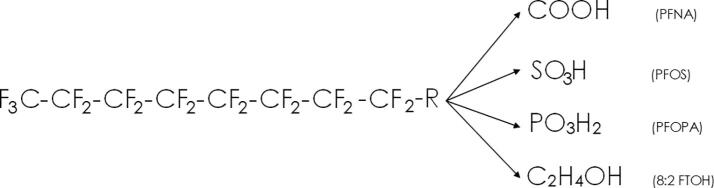
Example of a C8 perfluorocarbon chain and four possible functional groups of PFAS, top to bottom: Perfluorononanoic acid (PFNA), Perfluorooctane sulfonic acid (PFOS), Perfluorooctyl phosphonic acid (PFOPA) and, 8:2 Fluorotelomer alcohol (8:2 FTA) [19].

PFAS molecules contain the perfluoroalkyl moiety (C_n_F_2n+1_), which is commonly C4-C8 in length and arranged linearly. Shorter, longer and branched perfluoro chains also exist and some carbons within the chain may not be fluorinated. Non-fluorinated regions in the PFAS chain differentiate a partially (poly-) fluorinated PFAS from a totally (per-) fluorinated one [18] (Fig. 1).

The high degree of fluorination makes PFAS thermally stable and unreactive due to the strength (≈440 - 530 kJ mol^−1^) and shortness (≈1.3 – 1.4 Å) of C—F bonds and electrostatic and steric shielding of C—C bonds by fluorine atoms [20], [21]. PFAS have a plethora of non-fluorinated headgroups (Fig. 1), which are often hydrophilic, while the perfluoro-chain is hydro- and oleo-phobic, making many PFAS useful surfactants [20]. Variations in length, branching, fluorination and headgroup have enabled at least 9,252 different PFAS structures to be developed [19], [22]. Structural variations also give specificity for certain applications, which include; waterproofing, foaming agents, non-stick coatings, paper additives and metal plating aids, amongst many others [3].

The high solubility, number of different structures, and lack of emissions data makes quantification of global PFAS pollution extremely challenging [11], [23]. Historical PFAS production and sale was as semi-quantified mixtures of branched and unbranched, variable length, mixed isomer components [2], [24], [25], leaving users unable to quantify their emissions [18]. Globally, historic PFAS pollution is broadly estimated at 10,000 to 53,000 tonnes, with the majority presumed to be aqueous [11]. The actual amount is likely much higher, since current production is estimated at 42,000 tonnes/year [26] and stockpiled AFFFs are estimated at 30,000 tonnes, with 19,000 tonnes in Japan alone [27]. Since PFAS are inert by design, many are unaffected by environmental degradation processes and conventional water treatments, as reviewed previously [24], [28]. Although, select polyfluorinated compounds undergo partial degradation to form other perfluorinated PFAS [29], causing some waste water treatment plants (WWTPs) to be net PFAS emitters [24], [30]. Therefore, PFAS pollution remains largely undetected, dilute, and ubiquitous worldwide. Further reading on PFAS definitions [18], environmental distributions [11], and health effects [31] is available in several works [32].

Sonolysis is a physio-chemical treatment which splits molecules by applying ultrasonic waves (frequency ≥ 20 kHz). Sonolysis degrades many unreactive organic compounds including; chloro-hydrocarbons [33], esters [34], phenolic compounds [35] and PFAS [36], [37], [38], [39], [40], [41], [42], [43], [44], [45], [46], [47], [48], [49], [50], [51], [52]. Ultrasound can degrade PFAS in pure aqueous solutions [37], [38], [39], [40], [41], [42], [43], [44], real and simulated ground waters [46], [47], diluted AFFFs [49], [50], and soil slurries [51]. Research to date has focused on the parametric understanding of factors such as; chain length [41], ultrasonic frequency [36], PFAS concentration [45] and co-contaminants [46], [48], [49], [50]. Work has even utilised a large scale (91 L) reactor for treatment of a pure PFOS solution [53] and several PFAS within diluted AFFFs [50]. Sonolysis mineralises PFAS into inorganic CO, CO_2_ and F^-^ [39], without the addition or production of harsh chemicals. Gaseous or aqueous fluorinated by-products represent <1 % of the initial PFAS mass [36] since these are also degraded with continued sonication [39], [41], [36], [37].

No universally agreed theory exists for the PFAS-ultrasound reaction mechanism; thus, one cannot confidently identify all rate controlling parameters. Despite ≈30 experimental works to date, the interconnected nature of sonochemical parameters (frequency, power, temperature etc.) and the different conditions used in each work, obstruct understanding of any one parameter. To utilise PFAS sonolysis at an environmentally beneficial scale, researchers must understand the numerous works on the topic to date. A literature review on PFAS sonolysis has been completed previously which considered the effects of various reaction parameters [54]. The focus of this *meta*-analysis, however, is to derive novel information on reaction mechanisms, parametric effects, reaction monitoring methods and optimal reactor/reaction design, by comparison between works. Concepts are discussed from works in the field of sonochemistry, separate to that of PFAS, to improve understanding. Finally, comparison to other PFAS treatment technologies is presented to give context to the effectiveness and competitiveness of sonolysis. The authors assume the reader is familiar with the basic theory of sonochemistry, however, further reading on sonochemical mechanisms [55], [56] and parametric effects [57] is available elsewhere. A list of acronyms is given in Table S1 of the Supplementary information. To demonstrate the progression of the subject knowledge, Table S2 provides a chronological summary of the topics discussed in each work to date, and Table S3 shows all data usage in the *meta*-analysis.

## 2 Methodology – Measurements of PFAS sonolysis reaction products

PFAS sonolysis results in four main inorganic species; F^−^, SO_4_^2–^, CO, and CO_2_ [36], [48], [52], [38], [39], [58], [59]). Small quantities of shortened PFAS have also been observed [36], [37], [49], [50], [60], [61], [62], [63] or implied by F^−^, SO_4_^2–^, and total organic carbon (TOC) balances [42], [44], [48], [64], [65], but products with longer chains than the initial PFAS have not been reported. For the stoichiometric equations leading to these products see Section 3.2. Some have also measured pH [50], [58], [60], OH radicals [42] and nitrite/nitrate levels [38] as a means for identifying the PFAS degradation route. The rates of PFAS sonolysis under each set of conditions are required to quantitatively compare parametric effects across numerous works. However, different metrics for reaction rate or reaction completeness are often used. In this section, we describe these metrics and the technologies used to measure them to aid understanding in proceeding sections.

### 2.1 Fluoride release

Fluoride ion concentration, as a measure of PFAS degradation, is determined by ion chromatography [36], [60], [61], [62] or fluoride-selective electrodes [42], [44], [50], [51], and the two devices show strong agreement (±1 %) [36]. Although most PFAS are fully mineralised during sonolysis at mid-high frequencies (100 – 1,000 kHz) [41], [62], [36], [37], fluoride concentration alone does not necessarily correlate with PFAS degradation rates, since partial defluorination may occur, resulting in shorter PFAS chains (including polyfluorinated species) formed from the starting molecules [36], [42], [44], [66]. Notably high concentrations of short chain/partially fluorinated products are formed under low frequency sonication [60], [62], [63]. Percentage fluoride release is, therefore, used to demonstrate reaction completeness (percentage mineralisation) (Eq. (1)).

(1) $$\%\;F^{-}release=\frac{\left[F^{-}\right]}{\left[{\mathrm{TOF}}_{0}\right]}\times 100\;\%$$

Where:

$\left[F^{-}\right]$ = Fluoride ion concentration (mol L^−1^)

$\left[{\mathrm{TOF}}_{0}\right]$ = Total organic fluorine at time t = 0 (mol L^−1^)

  TOF_0_ is measured by combustion ion chromatography [49] or calculated from initial PFAS concentrations. Fluoride release varies with time and applied conditions, but always tends toward 100 %, since short chain by-products typically contain <1 % of initial fluorine atoms and are degraded with continued sonication [41], [62], [36], [37]. Short chain PFAS formation can thus be indicated using a fluoride balance (Eq. (2)) [39].

(2) $$\%\;fluorine\;balance=\frac{\left[F^{-}\right]+\left[F\;in\;unreacted\;PFAS\right]}{\left[{\mathrm{TOF}}_{0}\right]}\times 100\;\%$$

Where:

$\left[F\;in\;unreacted\;PFAS\right]$ = $\sum_{a}^{n}\left[Unreacted\;{\mathrm{PFAS}}_{a}\right]\times fluorine\;molarity\;per\;{\mathrm{PFAS}}_{a}\;mol$

ForninitialPFAScompounds

Eq. (3) thus identifies the contribution of fluorine in all short chain by-products. Note that fluoride containing gases likely represent <0.1 % of fluorine in the system, F_2_ formation has not been observed [39] and a small amount of HF gas has only been noted in one study to date [51].

(3) %fluorideinshortchainproducts=100%-%fluorinebalance

Fluorine balances simplify identification of short chain products, which requires analytical standards of all possible by-products and mass spectrometry (MS). This is especially difficult in complex starting matrices like AFFFs. During one study on PFOA and PFOS (PFOX) sonolysis, shortened species were not identified, but incomplete fluoride balances were reported during treatment [39]. The fluorinated gases formed did not account for the lack of fluorine, suggesting formation of aqueous fluorochemicals not tested for or below limits of detection (LODs) [38]. Other authors have measured defluorination efficiency [47] (Eq. (4)), which is taken relative to the amount of fluoride which should be expected from complete mineralisation of any measured PFAS loss (excluding non-PFAS fluorine). [47]

(4) $$\%\;defluorination\;efficiency=\frac{\left[F^{-}\right]}{F\;molarity\;per\;\mathrm{PFAS}\;\mathrm{molecule}\times [degraded\;PFAS]}\times 100\;\%$$

However, percentage fluoride release and defluorination efficiency can differ. In one experiment, the degradation of 120 µM PFOA under 150 W of 40 kHz ultrasound for four hours yielded 24.4 % PFOA removal but only 5.8 % fluoride release [58]. Hence, % F^−^ release is not always an indication of mineralised PFAS concentrations. One can calculate the defluorination efficiency (23.7 %) which indicates incomplete mineralisation of PFOA and the formation of partially fluorinated by-products.

### 2.2 Sulfate release

Like fluoride, sulfate (SO_4_^2−^) release and sulfur balance (Eq. (5), Eq. (6)) are accounted for via ion chromatography [39], [42], [44], [48], [49]. Sulfur balance also varies with time and applied ultrasonic conditions but is typically proportional to the removal and oxidation of SO_3_^−^ headgroups from per- and polyfluorosulfonic acids (PFSAs) and tends toward 100 % with time [39]. Both sulfur and fluorine balances exceeding 100 % have also been reported [39] and should therefore be subject to background subtraction and error analysis.

(5) $$\%\;Sulfur\;release=\frac{\left[{\mathrm{SO}}_{4}^{2-}\right]}{\left[S\;in\;the\;initial\;PFSAs\right]}\times 100\;\%$$

(6) $$\%\;Sulfur\;balance=\frac{\left[{\mathrm{SO}}_{4}^{2-}\right]+\left[S\;in\;unreacted\;PFSAs\right]}{\left[S\;in\;the\;initial\;PFSAs\right]}\times 100\;\%$$

### 2.3 Carbon balance

CO and CO_2_ are thought to be formed during PFAS sonolysis from the oxidation of the defluorinated carbon chain, and removal of –COO- groups in per- and polyfluorocarboxylic acid (PFCA) sonolysis [39]. CO and CO_2_ formation has been measured using electron ionisation mass spectrometry [39]. Like fluoride release, CO or CO_2_ formation cannot demonstrate percentage PFAS degradation in isolation, since CO_2_ can dissolve in solution to form carbonic and formic acids, or degas from solution [67]. Carbon accounting is typically done by measuring total organic carbon (TOC) bound in PFAS molecules [40], [44], [49], [53] (Eq. (7)). Percentage TOC removal may differ from percentage PFAS, fluoride, or sulfur change, hence a carbon balance may be used (Eq. (8)).

(7) $$\%\;TOC\;removal=(1-\frac{\left[TOC\;in\;unreacted\;PFAS\right]}{\left[TOC\;in\;initial\;PFAS\right]})\times 100\;\%$$

(8) $$\%\;Cabon\;balance=\frac{\left[CO\right]+\left[{\mathrm{CO}}_{2}\right]+\left[TOC\;in\;unreacted\;PFAS+carbonic\;acid\right]}{\left[TOC\;in\;initial\;PFAS\right]}\times 100\;\%$$

  

### 2.4 Chromatographic short chain measurement

The measurement of inorganics can suggest the extent of mineralisation but, as mentioned, care should be taken since the % removal of PFAS and % generation of inorganic products are not equal when partial defluorination, chain shortening, or sulphate head group recombination occurs. Such reactions will be discussed in more detail in Section 3.1. Therefore, it is best to directly measure the concentration of the starting PFAS as well as several possible by-product PFAS. Reactant and product PFAS concentrations are directly measured using (ultra [38], [68]) high performance liquid chromatography mass spectrometry (U)(HPLC-MS) [37], [39], [41], [45], [46], [47], [48], [50], [51], [59], [60], [61], [62], [63], [64], [65], [69], [70] or gas chromatography (GC) [66]. During chromatographic analysis, some authors utilised a total ion chromatogram (TIC), which identified and quantifyied several short chain PFAS products [37], [49], [50], [60], [61], [62]. Others, however, analysed only specific compounds [58], [39], [40], [45], [46], even when fluorine or sulfur balances indicated chain shortening [43], [44], [48], [59], [64]. Anticipating which by-products to measure is difficult for more novel PFAS (e.g. perfluoroether carboxylates, PFECs) which have different reaction mechanisms (Section 3.2) and products [61] (Table S2) to typical PFAS compounds. Therefore, TICs help to identify unknown by-products. Observation of no truncation by-products must be complimented with other metrics, since this may be due to analytical method errors or lack of products tested for and contradicts many previous findings [37], [49], [50], [60], [61], [62]. PFAS analysis via HPLC-MS/MS is fraught with difficulties, such as contamination from Teflon coated components [2] and sample container caps [45]. For accurate testing, these should be replaced with stainless steel or polyetheretherketone alternatives [71]. The choice of chromatography solvents also affects measured PFAS concentrations and a high methanol fraction in the early stages of separation is recommended to allow appropriate detection time [72]. This may explain why PFAS chain shortening was observed in some works [37], [51], [60], [61], [63] but not some others [38], [47], [58], since short chains are more stable in water and may be flushed too quickly through the column/detector [41].

### 2.5 Choice of measurements and products to monitor

Direct PFAS measurement does not demonstrate the treatment effectiveness unless short chain formation is also quantified. Hence, several metrics are needed to determine the extent of PFAS removal. 100 % F^−^ release might serve as the best *individual* indicator of “treatment completeness”, since this is consistent with complete mineralisation (no short chain organic remnants) [73], provided that the analysis is accurate. PFAS sonolysis products are consistently smaller than the initial species [36], [37], [49], [50], [60], [61], [62] and eventually fully mineralised [63], [36], [37]. PFSAs can convert into comparable chain length perfluorocarboxylic acids (PFCAs) [63], [36], [37] but the reverse is not possible without sulphur present. Thus, with or without observation, one can predict all plausible fluorinated by-products from a given starting PFCA by noting down the structure, removing a CF_2_ group, and repeating until no CF_2_ groups remain. One can take a similar approach for PFSAs but considering the truncated chains with both SO_3_H and COOH headgroups. Based on these observations, Table 1 derives potential and detected PFAS truncation products from the sonication of several common starting PFAS. This table thus provides, for the first time, all fluorinated breakdown products for the PFAS mentioned and demonstrates a methodology with which to predict the breakdown products of other (for example longer chain) PFAS. This may aid understanding and quantifying of PFAS sonolysis by-products and thus understanding of their reaction mechanisms. The table provides PFAS anion *m*/*z* values for use during mass spectrometry; hence the complete molecules have an added H^+^ ion and mass = (*m*/z * z) + 1. For those considering more novel PFAS structures, Table S4, in the Supplementary information derives observed and plausible breakdown products for some PFECs, which degrade via a different truncation mechanism and are not thought to convert to (nor be formed from) non-ether containing PFAS (Section 3.1). No standard acronym convention was found for PFECs, so names were derived as shown in Table S5. These tables can be used in the selection of analytical standards/techniques, especially in considering that the number of possible breakdown products increases linearly with chain length.

**Table 1:** *m*/*z* values of common PFAS ions and their breakdown products during sonolysis.

Starting ion		PFOS	PFHpS	6:2 FTS	PFOA	PFHxS	PFHpA	PFPeS	4:2 FTS	PFHxA	PFBS	PFPeA	PFPrS	PFBA	PFES	PFPrA	PFMS	PFEA	PFMA
m/z value:		499	449	427	413	399	363	349	327	313	299	263	249	213	199	163	149	113	63
Breakdown products																			
Ion	m/z value:																		
PFOS	499														Key		Meaning		
PFHpS	449	✓													■		Plausible		
6:2 FTS	427														✓		Observed		
PFOA	413	✓																	
PFHxS	399	✓	■																
PFHpA	363	✓	■		✓														
PFPeS	349	✓	■			■													
4:2 FTS	327																		
PFHxA	313	✓	■	■	✓	■	■												
PFBS	299	■	■			■		■											
PFPeA	263	✓	■	■	✓	■	■	■		■									
PFPrS	249	■	■			■		■			■								
PFBA	213	✓	■	■	✓	■	■	■	■	■	■	■							
PFES	199	■	■			■		■			■		■						
PFPrA	163	✓	■	■	✓	■	■	■	■	■	■	■	■	■					
PFMS	149	■	■			■		■			■		■		■				
PFEA	113	✓	■	■	✓	■	■	■	■	■	■	■	■	■	■	■			
PFMA	63	■	■	■	✓	■	■	■	■	■	■	■	■	■	■	■	■	■	
	Reference:	[36], [37], [63]			[37], [60], [62], [63]														

## 3 PFAS sonolysis *meta*-analysis

The use of ultrasonic waves to degrade aqueous contaminants (sonolysis) was previously considered an “auxiliary” treatment [74] likely due to its higher power requirements compared to traditional water treatment techniques (e.g., anaerobic digestion, granular activated carbon, membrane filtration, ion exchange etc.). However, for particularly recalcitrant contaminants, such as PFAS, traditional water treatment approaches are ineffective [75], [76], [77], [78], [79]. Hence, sonolysis research since the late 1990s and early 2000s, has focused on more novel contaminants such as; carbon tetrachloride [80], trichloroethane [81], bisphenol A [82], alachlor [83], methyl *tert*-butyl ether [84], chloro-hydrocarbons [33], esters [34], phenolic compounds [35] and PFAS [36], [37], [38], [39], [40], [41], [42], [43], [44], [45], [46], [47], [48], [49], [50], [51], [52]. Sonolysis offers comparable treatment capabilities to some physical removal methods but without the need to regenerate the removal media. Further, the PFAS are degraded by the technology, which mitigates another treatment step [75], [76], [77], [78], [79]. Sonolysis works by generating cavitating bubbles in solution by irradiation of ultrasonic waves [55]. Upon collapse of cavitating bubbles, temperatures of several hundred degrees and pressures of several hundred bar are generated within an area localised to the microscopic bubble [55], [56], [57]. These conditions can result in plasma and radical species generation, which can both degrade dissolved contaminants, the extent to which either act depends on the ultrasonic conditions applied and the species to be treated. For further reading on sonochemical mechanisms [55], [56] and parametric effects [57], please see previous works. Within the research to date on PFAS sonolysis, there is no unified theory on the mechanism of PFAS degradation, although there is evidence for some mechanistic steps. Further, several parametric effects have been studied but with some differing conclusions. Due to the wide variety of reaction conditions used, comparisons between works is difficult.

Accordingly, this work presents an analysis of the 33 PFAS sonolysis papers to date, to generate novel information on the mechanisms, products, and parametric effects of PFAS sonolysis. Theoretical PFAS degradation mechanisms are derived by comparing arguments and data from PFAS sonolysis publications, in combination with sonolysis concepts and data from PFAS degradation via other techniques (e.g. pyrolysis, plasma degradation, etc.). Where possible, optimum parameter values (pH, temperature, frequency, etc.) for enhanced PFAS destruction rates are derived through comparison of sonolysis rates under different conditions. By considering the different conditions used between works, information on the relative effect of each parameter is analysed. In this section, a distinction is made between mid-high (100 – 1,000 kHz) and low-frequency (<100 kHz) sonolysis, since at mid-high frequencies, PFAS degrade quickly (0.002 – 0.04 min^−1^) without the addition of oxidative agents, compared to low frequencies (0.0002 – 0.03 min^−1^) [47], [85], [60], [61]. A second distinction is made between long and short chain PFAS which will be taken as those with ≥ 6 and ≤ 5 carbons in the perfluoro chain, respectively, as defined previously [18].

### 3.1 PFAS degradation mechanisms at mid-high ultrasonic frequencies (100 – 1,000 kHz)

**Fig. 2:**
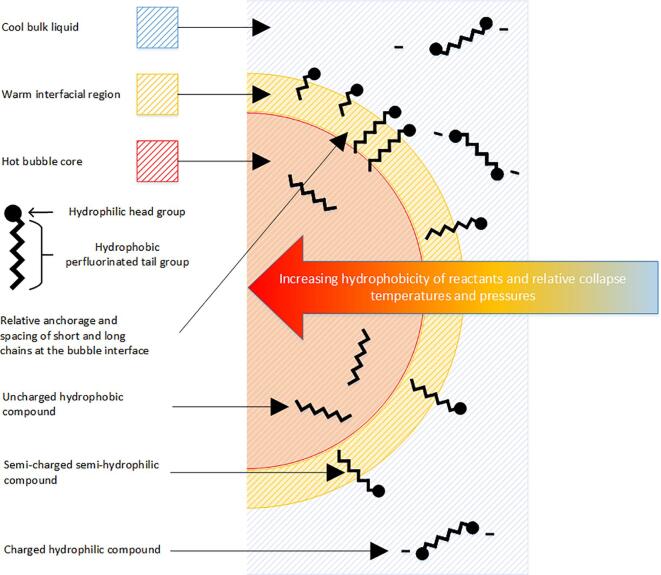
Position and relative collapse temperatures and pressures experienced by hydrophilic, semi-hydrophilic and hydrophobic surfactants, as well as relative anchoring and spacing of short and long chains at ultrasonic cavities. Not to scale.

Sonolysis of PFAS was first reported at a frequency of 200 kHz and power density of 3,333 W L^−1^ [37]. Sonication of water causes growth and formation of cavitation bubbles [55], [57]. When the bubbles reach a certain size, they move to antinode regions and collapse violently; causing disassociation/ionisation of water, gases and other species inside the bubble and high localised temperatures [57]. Aqueous PFAS behave as surfactants [8], hence PFAS sonolysis likely occurs predominantly at the bubble air-water interface, since prior to bubble collapse, the hydrophilic head should orientate in the bulk liquid, with the hydrophobic perfluoro-tail in the bubble gas phase [37] (Fig. 2). PFAS molecules are small (≈1 nm length [86], 75 – 477 cm^3^ molar volume [87]) when compared to ultrasonic bubbles (∼3.2 – 3.5 μm ambient radius, ≈10^14^ cm^3^ molar volume for a 355 kHz cavity under 2.5 – 4.6 bar pressure [88], [89]). Since the relative penetration of the molecule into the bubble core is small, we propose that, prior to cavity collapse, PFAS effectively adsorb at the bubble surface, rather than absorb/evaporate into the bubble core. Thus, adsorption to the bubble interface is considered the initiating step for PFAS sonolysis and PFAS diffusion to the bubble is likely rate limiting [42], [48], [37], [38], [44], [45], [65], [66]. Some authors question whether PFAS-bubble adsorption might not be diffusion dependant, since rectified bubble growth occurs over much faster time scales than the molecule can passively diffuse toward it and the bubble interface would move towards the PFAS, rather than vice versa [41], [45]. The implications of this for the rate-limiting mechanism of bubble surface adsorption are discussed further in Section 3.3.

Increasing PFAS concentration enhances reaction rates until, at sufficiently high concentration, rates became constant [38], [42], [48], [44], [45]. The reverse occurs when degrading high initial PFAS concentrations, as the reaction transitions from zero to pseudo-first order as concentrations decline [36]. The change in reaction order supports PFAS adsorption being rate limiting and reaction initiating, since it is consistent with PFAS saturation of the available bubble interfacial area [38], [42], [48], [44], [45]. Further evidence of interfacial adsorption was noted when the degradation rate of PFOA, an anionic surfactant, was reduced by addition of another anionic surfactant at 40 kHz and 500 W L^−1^, suggesting competitive adsorption at the bubble interface [65] (further discussed in Section 3.5.7). Similarly, sonolysis reaction rates for three different PFAS (perfluorooctane, perfluorooctanoic acid, and perfluoropropionic acid) were proportional to their relative hydrophobicity values, attributed to orientation of hydrophilic, semi-hydrophilic and hydrophobic molecules in the bulk liquid, bubble interface and bubble-gas core, respectively [66]. These regions experience relative collapse temperatures and pressures which increase with proximity to the bubble core (Fig. 2) [57]. Table 2 shows the log(K_OW_) values of various PFAS and some of their carboxylic acid equivalents (from previous works [90], [91]) to indicate their relative hydrophobicities. In agreement with aforementioned PFAS sonolysis studies, hydrophobicity increases with increasing chain length [66] and with replacement of fluorine atoms, with hydrogens [37]. Note however, that K_OW_ values are not easily measured for surfactant PFAS, due to their preference for interfacial regions, and researchers must utilise non-traditional methods [90]. Semi-hydrophobic (i.e. surfactant) PFAS like PFOA and PFOS accumulate at the bubble interface [66] and are subject to (multi-bubble) temperatures and pressures of ≈1,000 K [92] and 100 bar, respectively, upon bubble collapse [57]. Note that previous work on surfactant sonolysis suggests that all parts of a surfactant experience approximately the same collapse temperature [93], which is consistent with the relative sizes of the bubbles and surfactants described here.

**Table 2:** K_OW_ of PFCAs [90] and equivalent carboxylic acids [91].

Carbon chain length	PFAS	Log (k_OW_)	Carboxylic acid	Log (k_OW_)
1			Formic acid	−0.54
2			Acetic acid	−0.17
3			Propanoic acid	0.33
4	PFBA	−0.62	Butanoic acid	0.79
5	PFPeA	−0.02	Pentatonic acid	1.4
6	PFHxA	0.59	Hexanoic acid	1.9
7	PFHpA	1.19	Heptanoic acid	2.4
8	PFOA	1.79	Octanoic acid	3.1
9	PFNA	2.40	Nonanoic acid	3.4
10	PFDA	3.00		
11	PFUnA	3.51		
12	PFDoA	4.21		

**Fig. 3:**
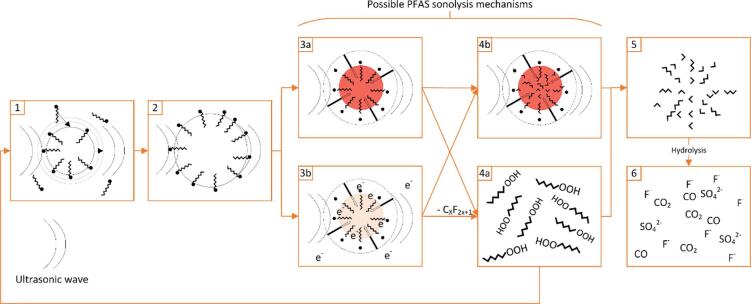
Competing PFAS sonolysis mechanisms at mid-high frequencies: PFAS adsorption to bubble interface under ultrasound (1), bubble growth to critical size (2) bubble collapse and reaction initiation via thermolysis (3a) vs solvated electron attack (3b), release of truncated perfluoro moiety and repeated oxidation-truncation (1-4a loop) vs sono-intermediate pyrolysis in bubble core (4b), formation of C1-C2 intermediates (5) and intermediate hydrolysis to end products in liquid bulk (6). Not to scale.

Following interfacial adsorption, PFAS degradation is suggested to begin via pyrolytic cleavage between the headgroup and perfluoro tail [42], [48], [37], [38], [44], [45], [65], [66] (Fig. 3). This was concluded since SO_4_^2–^ and shortened PFSAs and PFCAs formed during PFOS sonolysis, indicating removal and subsequent oxidation of the SO_3_^−^ headgroup, while partially maintaining the perfluoro chain [37]. Several works also observed SO_4_^2–^ concentrations stoichiometrically consistent with measured PFOS removal, but slower F^−^ removal rates, suggesting delayed tail degradation [39], [44], [48]. Note that not all authors have observed chain shortening during PFOS sonolysis [39], [68], [94], and two showed greater stoichiometric F^−^ release than SO_4_^2–^, suggesting that partial tail-defluorination may occur before headgroup cleavage [42], [44]. However, this may also be due to recombination of cleaved SO_4_^2–^ and CF_3_(CF_2_)_n_- groups, to form shortened PFSAs in solution [37] which were not measured.

Headgroup cleavage is a more likely initial degradation step than defluorination, since the C-F bond energies (≈440-530 kJ mol^−1^ [20], [21]) exceed that of the C—C bonds (≈348 kJ mol^−1^ [21]) or C-S bonds (≈301-355 kJ mol^−1^ [95], [96]) between the perfluoroalkyl chain and the headgroup. Further, these C—S and C—C bonds would not be subject to the same fluorine-shielding effects as the C—C bonds in the perfluoro-chain [20], [21]. This is consistent with computational work on the thermolysis of PFOS [97] and other fluorochemicals [98] which showed headgroup cleavage as the initial step. Maximum vibrational temperatures reached inside a bubble under mid-range frequencies are around 4,000 – 7,000 K [55], [99], [100], suggesting C-F bond cleavage may be possible. However, these temperatures are achieved for only a few nano- or peco-seconds for single-bubble sonication [101]. Such temperatures likely do not exist in multi-bubble sonication, where collapse temperatures are ≈ 1,000 K [57].

An argument against pyrolysis-driven head-group cleavage considers that, at concentrations below their critical micelle concentration (CMC), PFAS would orientate non-uniformly at the bubble-interface, thus not necessarily be cleaved at the head-group [38]. However, this contradicts numerous other observations [42], [48], [37], [38], [44], [45], [65], [66] and the comparison of ultrasonically induced PFAS-bubble diffusion with hydrophobically induced micelles may not be valid. Micelles form in the bulk liquid, thus, the adsorbed PFAS-bubble interfacial system might be better compared to a PFAS hemi-micelle, which forms on surfaces. Further, the hemi-micelle of PFOA, for example, can occur at concentrations of 1 mg L^−1^ [102], which represents <0.001 % of the CMC [103]. Such concentrations are common during PFAS sonolysis [36]. The high frequency oscillations of the cavities can also contribute to PFAS adsorption [41] and literature on the sonolysis of other surfactants demonstrated accumulation at the bubble below the CMC [104]. Hence, uniform PFAS adsorption at cavity surfaces is not entirely hydrophobically dependant and therefore not CMC dependant.

Accepting the evidence for interfacial PFAS adsorption, uncertainty remains over the mode of headgroup cleavage. In ultrasonic systems, dissolved gases with high specific heat ratios (polytropic indices, γ = C_p_/C_v_), such as Argon (γ = 1.66), generate cavities with high collapse temperatures [105], and are also correlated with better PFAS degradation rates [37], [61], [62]. For reference, the polytropic index of Air at 20 °C is 1.40 [47], [62]. This suggests that headgroup removal is thermally dominated, as opposed to via radical oxidation, as is common for other compounds [37], [106], [61], [62]. Further, radical degradation was rejected for PFOA and PFOS, since the Fenton reaction, which utilises hydroxyl radicals (OH), did not degrade these PFAS [37]. When *tert*-butyl alcohol was used to scavenge radicals, the degradation rate of PFOS was reduced by only 12 % and the effect on PFOA was inconclusive [37]. Conversely, correlations between OH formation and PFAS destruction were observed at applied frequencies from 25 - 1,000 kHz, suggesting a chemically driven process [42]. However, the reactor volume, shape, power densities, position of transducers and open/closed nature of the reactor was varied between frequencies [42]. Hence, these trends may not be entirely reflective of frequency/radical production alone, but several parameters. PFOS degradation rates under 44 – 1,000 kHz ultrasound have also been correlated with a sonochemical route, measured via sonochemiluminescence and potassium iodide dosimetry, rather than an entirely thermal one, measured via sonoluminescence [36]. Hence, an initial degradation route via solvated electrons at or near the surface of the collapsing bubble was discussed [36]. Several works have theorised [107], [108], [109], [110] and observed [111], [112] solvated electron release during sonolysis and solvated electrons are also theorised in the degradation of PFOS in other advanced oxidative techniques, including; electrochemical oxidation [113], photochemical oxidation [114] and plasma treatment [115]. To the authors’ knowledge, PFOS degradation has not been observed under any technology without an associated solvated electron reaction theory. Semi-empirical work on PFAS degradation via aqueous electrons showed that different PFAS structures show different degradation routes, with all PFAS tested except PFCAs, showing partial defluorination [116]. Solvated electrons are short-lived (≈100 ps in plasma systems) but can penetrate to liquid depths of 1.5 – 3.5 nm [117]. Thus, aqueous electrons released from the plasma of an ultrasonically generated cavity are capable of attacking anywhere on an adsorbed PFAS molecule (length ≈1 nm [86]), the majority of which (the perfluoro-tail) will be within the cavity, and the tail-headgroup bond will be close to the interface. Since the remaining C—C bonds are well shielded by fluorine atoms and the C-F bond energy is strong, chemical attacks are not typically successful at degrading PFAS, particularly PFOS. However, solvated electrons may be small enough to attack more positive regions on the carbon-fluorine bonds, since a strong dipole exists due to the high electron withdrawing nature of fluorine atoms, which attract electrons toward themselves, away from the carbons [20]. Solvated electrons are also thought to cleave C-Cl bonds during chloroacetate sonolysis [118], so may similarly cleave C-F bonds in PFAS sonolysis.

After headgroup removal, two mechanisms are proposed for the perfluoroalkyl chain degradation: 1) repeated oxidation and truncation and 2) pyrolysis within the bubble, in one collapse event. Repeated truncation is evidenced by the formation of PFOA and shorter PFCAs during PFOS degradation [36], [37]. PFCA formation was attributed to oxidation of the removed CF_3_(CF_2_)_n_- tails in the liquid bulk to form a shortened PFCA [CF_3_(CF_2_)_n-1_COOH] [37]. The shortened PFCA would then be adsorbed to a new bubble interface and the cycle would repeat, truncating one CF_2_ group per cycle, until the initial PFAS is mineralised into F^−^, CO and CO_2_. Similar mechanisms were identified for the hydrocarbon equivalents of PFOA and PFOS (n-octanoic acid and 1-octanesulfonic acid, respectively), however, this occurred 6.90 – 13.75 times faster due to the greater hydrophobicity of hydrocarbons [37]. The repeated truncation theory is supported by several direct measurements or indications of short chain formation (through partial defluorination and/or TOC removal) at both low (28 & 40 kHz) [60], [62], [63] and mid-high (200 – 1,000 kHz) [36], [42], [44], [66] frequencies. However short chains may also form through fragmentation of the initial perfluoro chain and reaction with water to form hydrated intermediates [39], which may then react via opposing condensation reactions, in which water is produced, when H and OH groups are removed from two combining molecules. Such reactions are assumed to occur at the bubble surface, as observed for other complex organic molecules, such as lignin [119]. Similarly, shortened PFSA formation during PFOS sonolysis was attributed to SO_3_^−^ headgroup removal and recombination with a truncated, possibly unbalanced, fissional C_n_F_2n+1_ group [37]. An argument against degradation solely via repeated truncation might be that longer PFAS show greater initial fluoride release rates [44]. Given the repeated truncation theory, regardless of initial PFAS chain length, similar removal of CF_2_ units would be anticipated per bubble collapse event. However, longer chains are more hydrophobic, making them more attracted to the bubble interface and penetrating further into the hot bubble core [41], and thus experience faster uptake, pyrolysis and, hence, defluorination.

The alternative theory, pyrolysis inside the bubbles, evolved from observation of near stoichiometric release of F^−^ ions consistent with complete PFOX mineralisation, without short chain formation [39]. Hence, headgroup cleavage was instead proposed to precede rapid degradation of the perfluoroalkyl chain via the formation of sonochemical intermediates, such as hydrated C_n_F_2n+1_-H groups, within one acoustic cycle [39]. Within the same cycle, the intermediates would be degraded via numerous plasma/pyrolysis reactions inside the bubble, to form C1 - C2 length fluorinated radicals [39]. The fluorinated radicals would then be hydrolysed by water vapour in the bubble to form CO, CO_2_ and HF (which dissolves into H^+^ and F^−^ in the bulk liquid [37], [45]) over approximately 100,000 acoustic cycles $\left(\approx \frac{1}{3}\;s\right)$ [39]. Rapid and complete mineralisation of the perfluoro chain may be plausible due to the high temperatures reached during aqueous bubble collapse (up to 5,000 K) [55], [99], [100]. However, as mentioned previously, questions remain over the lifetime of these temperatures and their applicability in multi-bubble systems [101]. Where shorter chain by-products were not detected, the bubble core pyrolysis theory was not supported with stoichiometric fluoride or SO_4_^2–^ release, nor TOC mineralisation, relative to the removed PFAS [38], [39]. Hence, it is possible that some shortened fluorochemicals formed which were not tested for, did not exceed the LOD or entered the untested gas phase above the reactor liquid (Section 2). Moreover, the reaction could proceed via a repeated truncation process which is simply too fast to observe short chain formation. This may follow since the half-lives of PFOA and PFOS were 22 and 43 min, respectively, where chain shortening was observed [37] and 17 and 26 min, respectively, where it was not observed [39]. Further, samples were taken from the reactor sooner in works where short chains were observed than where they were not (5 vs 15 min) [37], [39]. Conversely, short chain products ejected from the collapsing bubble, which did not fully mineralise under the intra-bubble pyrolysis mechanism, or which reform from radicals in the liquid bulk may lead to the appearance of products indistinguishable from those of a repeated truncation mechanism [37]. The competing mechanisms for PFAS sonolysis at mid-range frequencies are summarised diagrammatically in Fig. 3. This figure does not consider mechanisms suggested in low frequency works since there is less evidence for these mechanisms, they have been shown to be more varied (depending on oxidative species added), and the reaction rates are generally slower (0.0002 – 0.03 min^−1^) [47], [85], [60], [61] than at high frequencies (0.002 – 0.04 min^−1^) [47], [85], [60], [61]. Low frequency mechanisms are discussed further in Section 3.5.6.

The two theories (repeated truncation vs rapid intra-bubble pyrolysis) were derived using results taken under different experimental conditions (10 – 100 mg L^−1^ PFOX, degraded at 200 kHz and 3,333 W L^−1^ ultrasonic power [37] vs 5 mg L^−1^ PFOX, degraded at 618 kHz and 250 W L^−1^ power [39]). The higher concentration and use of a frequency considered to give slower PFOS degradation rates (200 kHz) [36], [37] (Section 3.5.1) would likely lead to the formation and observation of short chain intermediate products. However, chain truncation was also observed at lower concentrations (10 mg L^−1^) and more optimal frequencies (400 kHz and 500 kHz) [36], to a lower extent. The analytical evidence and arguments shown here indicate that PFAS undergo chain shortening over time, as opposed to solely instantaneous mineralisation, regardless of the reaction mechanism. Therefore, given strong evidence for both the repeated truncation and bubble core pyrolysis theories, as well as the observation of small quantities of short chains in some works but not others, we propose that both degradation routes may occur during PFAS sonolysis and that the extent of by-product formation therefore depends on the reaction conditions used (e.g. frequency, power, sampling/treatment time, concentration etc.).

### 3.2 Derivation of complete PFOX sonolysis reaction equations

Based on the mechanistic works discussed in Section 3.1, the first reported complete reaction equation for PFAS sonolysis can be derived. Previous attempts have not achieved a fully balanced equation. For example, a partial equation for PFOS degradation has been given previously [42] (*Eq.* (9)).

(9) $$C_{8}F_{17}{{\mathrm{SO}}_{3}}^{-}\overset{)))}{\to }{17F}^{-}+SO_{4}^{2-}+\sum C_{n}\left(n=1-8\right)$$

Where;

$\overset{)))}{\to }denotes\;reaction\;under\;ultrasound$

The stoichiometric balance of fluorine during PFOS mineralisation is easily derived, since, with no initial F^−^ or other fluorinated compounds in the solution, [F^−^]_final_ must equal [PFAS]_initial_ × 2n + 1 (where n is the initial perfluoro chain length). *Eq.* (9) assumes that all fluorine atoms are cleaved to generate fluoride. However, several shorter chain PFAS can also be generated during sonolysis, as discussed in Section 3.1, which have not been accounted for in *Eq.* (9). Further, the generation of organics (hydrocarbon or otherwise) from PFAS sonolysis has never been measured or observed, only assumed based on TOC measurements in the work where *Eq*. (9) was derived [42]. The organic products are assumed since only 55.8 % of TOC was removed after 330 min of sonication, while 100 % of fluoride release was achieved in under 300 minutes [42]. However, in said work, the starting TOC concentration for 100 μM PFOS was measured at 1,000 μM [42]. Since PFOS has 8 carbon atoms, the starting TOC for 100 μM PFOS should be 800 μM. Similar issues are found when considering the fluoride concentration, which takes a final value of ≈2.0 mM [42]. Since PFOS has 17 fluorine atoms, for the degradation of 100 μM PFOS, the total fluoride released should be 1.7 mM. Both of these inflated values suggest either some contamination or error in the samples or methods has occurred, which likely lead to over estimation of the final TOC value and defluorination %, thus the assumption of some organic by-products. Further, it is unlikely that non-fluorinated by products would be formed in any great quantity, since truncated PFAS by products are at low concentrations compared to the starting PFAS [39], [44], [48], [36], [37] and hydrocarbon equivalents of two PFAS (PFOA and PFOS) were previously shown to degrade 6.9 – 13.75 times faster due to the greater hydrophobicity of hydrocarbons [37]. Thus, any non-fluorinated organics would likely be degraded extremely quickly, difficult to detect compared to fluorinated by-products and thus cannot be considered significant by-products. Further, the stoichiometry of *Eq.* (9) is incomplete, since it does not state the complete formulae of the organics formed, nor suggest where the oxygen comes from in the oxidation of SO_3_^−^ to SO_4_^2–^.

Four inorganics species (H^+^, CO, CO_2_ and F^−^) are the dominant reaction products of PFAS sonolysis observed experimentally, with SO_4_^2–^ also generated in PFSA sonolysis [39], [59], [64], [36], [37] and some short chain PFAS are also formed prior to complete mineralisaition [39], [44], [48], [36], [37]. Since short chain PFAS products form with a complex distribution, are undesirable end products, and have been shown to degrade with continued sonication, they can be treated as intermediates [36]. PFOA and PFOS sonolysis, under conditions of 100 µM PFAS in argon sparged water sonicated for 120 min at 354 kHz and 250 W L^−1^, produced CO and CO_2_ in ratios of 2/1 and 5/1, respectively [39]. The authors of the aforementioned work did not propose a stoichiometric equation based on these observations, only some possible intermediate reactions [39]. Hence, assuming near-zero loss of gaseous products, sufficient sonication time for complete mineralisation and the measured CO/CO_2_ ratios [39], the overall reaction equations for PFOA and PFOS are shown by *Eq.* (10) and *Eq.* (11), respectively. H_2_ is listed here as a newly suggested by-product, which has not yet been observed during PFAS degradation but is known to occur during sonolysis in water [55], particularly in the presence of an Argon atmosphere [67]. Hence, H_2_ formation balances the hydrogen atoms formed from the splitting of water, required to oxidise PFAS to CO/CO_2_. All components in *Eq.* (10) and *Eq.* (11) are aqueous.

(10) $$3C_{8}F_{15}O_{2}H+26H_{2}O\overset{)))}{\;\to \;}8CO_{2}+16CO+45F^{-}+45H^{+}+10H_{2}$$

(11) $$3C_{8}F_{17}{\mathrm{SO}}_{3}H+31H_{2}O\;\overset{)))}{\to \;}4CO_{2}+20CO+51F^{-}+57H^{+}+3SO_{4}^{2-}+4H_{2}$$

In previous work, the oxygen source for CO/CO_2_ formation during PFAS degradation was not derived [39], but is likely from water sonolysis [67], as observed in the sonolytic oxidation of hydrocarbons [120]. The percentage conversions of TOC to CO/CO_2_ were 96.7 % for PFOA and 88.6 % for PFOS [39]. Increasing the relative percentage of water vapour in collapsing bubbles, and hence the relative radical concentrations formed, was suggested to enhance CO_2_ formation during PFOA sonolysis [39]. Since PFOS is a larger surfactant molecule than PFOA [18], greater microstreaming around the bubble is expected [121] and enhanced water evaporation rates and CO_2_ formation rates might be anticipated for PFOS, compared to PFOA, due to enhanced fluid refreshing around the bubble. This does not match experimental observation [39]. Hence, we propose that differing PFOX degradation rates explain the higher CO/CO_2_ formation ratio for PFOA. PFOS is more difficult to degrade than PFOA, due to the strength of the C-S bond [37], [38], [40], [41], [43], [44], [45], [46], [63], [68]. Further, bubble collapse temperatures can be reduced by bubble instability in the presence of large surfactant molecules, which may partially explain why PFOS degradation rates are lower than PFOA. As stated in the work which observed their formation, CO and CO_2_ must be generated from oxidation of CF_2_ radicals [39]. CO_2_ is therefore generated in smaller quantities than CO because it requires a higher level of oxidation. Therefore, compounds which degrade less effectively will generate CO more slowly, giving less time to oxidise to CO_2_. Hence, the CO/CO_2_ formation ratio is likely specific to the species being degraded, the ultrasonic reaction conditions used, and the time of sampling. This is confirmed by the data in the aforementioned work, which shows that the CO and CO_2_ concentrations have not yet reached an equilibrium state at the time of sampling (120 mins) [39]. Therefore, given sufficient sonication time, the CO would likely oxidise to CO_2_. Thus, CO can be treated as an intermediate and the complete reaction equations for PFOA and PFOS under ultrasound are given by *Eq.* (12) and *Eq.* (13), respectively. The conditions for these equations are similar to those for *Eq.* (10) and *Eq.* (11), but with a treatment time exceeding 120 mins.

(12) $$3C_{8}F_{15}O_{2}H+42H_{2}O\;\overset{)))}{\to \;}24CO_{2}+45F^{-}+45H^{+}+21H_{2}$$

(13) $$3C_{8}F_{17}{\mathrm{SO}}_{3}H+51H_{2}O\overset{)))}{\;\to \;}24CO_{2}+51F^{-}+57H^{+}+3SO_{4}^{2-}+24H_{2}$$

### 3.3 Rate limiting steps

Since PFAS sonolysis reaction mechanisms are subject to debate, rate determining steps are also disputed and suggested to depend on the specific reaction conditions and PFAS structures [41]. Since F^−^ and SO_4_^2–^ release rates are near constant at increasingly high PFOX concentrations, at mid-high frequencies, sorption to the bubble interface is the rate-initiating and rate-limiting step in PFAS sonolysis [38], [39], as shown for the sonolysis of pesticides [122]. However, based on the work of Fyrillas and Szeri [123], it was argued that passive diffusion of long PFAS molecules to the bubble interface cannot be rate limiting, since the bubble interface oscillates into the molecules’ path faster than the molecules diffuse towards it [45]. The degradation rate of small chains may still be limited by passive diffusion, due to their much smaller hydrophobicity (partitioning coefficients, K_eq_) and reduced tendency to adsorb to the interface, hence the enhancement of short chain PFAS reaction rates at higher frequencies (Section 3.1) due to their increased bubble oscillation rates [41], [45]. The authors of this *meta*-analysis also propose that, at low PFAS and/or bubble concentrations, passive diffusion toward the bubble may still dominate, since the bubbles may not always be within oscillating distance of the PFAS molecule.

Following adsorption to the bubble surface, a second rate limiting step must be head-group cleavage [38], [45] which should be limited by the rate of bubble collapse events [41]. This, in turn, is dependent on the applied ultrasonic frequency [41], [55] and power [124]. Following head-group cleavage, the reaction rate of the initial PFAS cannot be further limited, since it has been degraded. However, the rates of product generation can be further limited by the rate of rectified diffusion of the aqueous sonochemical intermediates into the bubble core [39] or the rate of short chain adsorption, which is dependent on the short chain volatility, applied ultrasonic frequency [41], [55] and power [124]. At higher frequencies there is an increase in diffusion of both gases and volatile species to the bubble surface [125]. The rate of fluorinated gaseous by-product absorption from the bubble vapour and the vapour head space above the bulk liquid, back into the bulk liquid may also limit by-product release. However, due to relatively fast collapse of the bubble (which is undergoing a reduction in surface area), and non-equilibrium condensation at the surface, one can assume that rectified diffusion back to the liquid is negligible [126], [127]. Further, since fluorinated gases in the reactor head space represent <0.1 % of the initial fluorine in solution (as measured by gas chromatography) [39], their re-absorption is not rate controlling.

The possibility that the reaction products themselves might be rate limiting or enhancing has yet to be discussed in other works. Any short chains formed are unlikely to become competitive species for the bubble interface due to their lower volatility than the initially longer starting species [41], [66], [43], [44], until the starting PFAS concentration drops below the bubble surface saturation point. Defluorination rates might be reduced, since any shorter chains formed are less well adsorbed by the bubbles [41] and degraded slower. However, this effect is limited since short chain intermediates/by-products represent a small proportion of the initial fluorine [36]. The effects of inorganic species on the rate of PFAS degradation have been studied previously and are shown to follow the Hofmeister series, i.e. inorganics which cause salting-out effects hasten PFAS transit to the bubble interface, which increases degradation rates [46]. The inorganics gather water molecules in their hydration sphere which would otherwise solubilise PFAS, hence reducing PFAS solubility and increasing their attraction to the bubble interface [128]. While the effect of fluoride on PFAS sonolysis was not studied previously [46], fluoride is known to have strong salting out effects in several studies reviewed previously [129]. Hence, fluoride formation may be rate enhancing for PFAS sonolysis. Conversely, sulfate is known to reduce PFAS reaction rates, by salting-in (increasing the solubility of) PFAS [46], hence PFSA degradation may be moderately self-limiting. CO_2_ is also a known by-product of PFAS sonolysis [39], which has been shown previously to limit the rate of iodine and H_2_O_2_ production during the sonolysis of potassium iodide (KI) at 300 kHz, under an argon atmosphere [67], similar to the conditions under which CO_2_ formation was observed [39]. Since both of these are commonly used metrics for the approximation of sonochemical activity levels, CO_2_ is likely slightly rate limiting for PFAS sonolysis as it will likely quench sonochemical effects. Given knowledge of the four rate-limiting steps, parameters that enhance reaction rates or help develop kinetic models to predict degradation rates can be reasoned, as discussed in the following section.

### 3.4 Kinetic modelling

To date, only single-bubble or very limited multi-bubble sonolytic systems have been accurately modelled [130], limiting prediction of real-world sonolysis reaction rates. However, using bulk properties, empirical data, and knowledge of rate limiting steps, such modelling can be simplified. Langmuir-Hinshelwood (LH) kinetics were applied to systems limited by surface area, such as sonolysis of volatile pesticides [122] and PFAS (*Eq.* (14)) [45].

(14) $$R_{\mathrm{PFAS}}=R_{{\mathrm{PFAS}}_{\mathrm{Max}}}\theta_{\mathrm{Sono}}^{\mathrm{PFAS}}=R_{P_{\mathrm{Max}}}\frac{k_{\mathrm{Sono}}\left[PFAS\right]}{1+k_{\mathrm{Sono}}\left[PFAS\right]}$$

Where:

$R_{\mathrm{PFAS}}=Reaction\;rate\;\left(mol\;L^{-1}s^{-1}\right)$

$R_{{\mathrm{PFAS}}_{\mathrm{Max}}}=Reaction\;rate\;when\;the\;bubble\;surface\;is\;saturated\left(mol\;L^{-1}s^{-1}\right)$

$\theta_{\mathrm{Sono}}^{\mathrm{PFAS}}=Fraction\;of\;PFAS\;molecules\;sorbed\;to\;the\;bubble\;interface\;(-)$

$k_{\mathrm{Sono}}=PFAS\;sonochemical\;interface\;partitioning\;coefficient\;\left(L\;{\mathrm{mol}}^{-1}\right)$

This model is also easily extended for multi-component PFAS systems [45] by summing the $k_{\mathrm{Sono}}\left[PFAS\right]$ terms for X number of individual PFAS (termed PFAS_1_, PFAS_2_ … PFAS_X_).

*Eq.* (15) [45]

(15) $$R_{1}=R_{1_{\mathrm{Max}}}\frac{k_{\mathrm{Sono}}\left[{\mathrm{PFAS}}_{1}\right]}{1+k_{\mathrm{Sono},1}\left[{\mathrm{PFAS}}_{1}\right]+k_{\mathrm{Sono},2}\left[{\mathrm{PFAS}}_{2}\right]+\cdots k_{\mathrm{Sono},X}\left[{\mathrm{PFAS}}_{X}\right]}$$

$$R_{2}=R_{2_{\mathrm{Max}}}\frac{k_{\mathrm{Sono}}\left[{\mathrm{PFAS}}_{2}\right]}{1+k_{\mathrm{Sono},1}\left[{\mathrm{PFAS}}_{1}\right]+k_{\mathrm{Sono},2}\left[{\mathrm{PFAS}}_{2}\right]+\cdots k_{\mathrm{Sono},X}\left[{\mathrm{PFAS}}_{X}\right]}$$

  


…

$$R_{X}=R_{X_{\mathrm{Max}}}\frac{k_{\mathrm{Sono}}\left[{\mathrm{PFAS}}_{X}\right]}{1+k_{\mathrm{Sono},1}\left[{\mathrm{PFAS}}_{1}\right]+k_{\mathrm{Sono},2}\left[{\mathrm{PFAS}}_{2}\right]+\cdots k_{\mathrm{Sono},X}\left[{\mathrm{PFAS}}_{X}\right]}$$

*Equation*(14) and *Equation* (15) apply only under high PFAS concentrations (≥15 - 40 µM), where bubble surfaces become saturated, and the reaction order is zero. At undersaturated concentrations ($k_{\mathrm{Sono}}\left[PFAS\right]\ll 1$), the reaction becomes first order, and the equation becomes simplified (Eq. (16)). [45]

(16) $$R_{\mathrm{PFAS}}=R_{{\mathrm{PFAS}}_{\mathrm{Max}}}\theta_{\mathrm{Sono}}^{\mathrm{PFAS}}=R_{P_{\mathrm{Max}}}k_{\mathrm{Sono}}\left[PFAS\right]$$

LH kinetics showed that PFAS interfacial adsorption due to bubble growth is approximately two orders of magnitude faster than passive equilibrium partitioning, for PFOX [45]. Alternatively, Michaelis-Menten (MM) kinetics modelling of PFOX degradation (Eq. (17)) showed that the concentration of sonochemically active bubbles depends on the species being degraded [38]. The bubbles were considered as catalysts with constant concentration and reactions were assumed to be reversible. Calculated active site concentrations for PFOA and PFOS were 89.25 mM and 8.8 mM, respectively, and this was explained by the greater required collapse temperature for PFOS degradation [41], [37], [38], [44], [45].

(17) $$R_{\mathrm{PFAS}}=\frac{R_{\mathrm{PFAS},Max}\left[PFAS\right]}{K_{M}+\left[PFAS\right]}$$

Where:

$R_{\mathrm{PFAS},Max}=k_{\mathrm{sono}}\left[B_{A}\right]$

$k_{\mathrm{sono}}=Rate\;constant\;for\;conversion\;of\;PFAS\;bound\;to\;active\;bubbles\;into\;\mathrm{intermediates}\;({\min\;}^{-1})$

$\left[B_{A}\right]=Concentration\;of\;sonolytically\;active\;bubbles\;(mol\;L^{-1})$

$K_{M}=Overall\;rate\;constant\;for\;the\;creation\;and\;destruction\;of\;active\;bubbles$

Equation (17) describes MM kinetics for first order PFAS sonolysis, however the original authors of the work [38] did not discuss how this might change under sufficiently high concentrations to become zero order, i.e. when $K_{M}\ll \left[PFAS\right]$. This is described here by Eq. (18).

(18) $$R_{\mathrm{PFAS}}=R_{\mathrm{PFAS},Max}$$

LH and MM kinetics are useful for surface limited reactions; however, they are usually applied to solid catalyst reactions, which have constant active surface area. During sonolysis, the bubble size, concentration, distribution, and surface area are dynamic. Further, the available surface area and coalescence of bubbles will be dependent on surfactant type and concentration [131], i.e. PFAS concentration, which is also dynamic. Bubble motion and shape stability are also dependant on the applied ultrasonic frequency, power and reactor geometry [57], [132]. This leads to some incompatibility in the LH and MM models. LH models can over estimate interfacial adsorption for less-volatile species (i.e. PFAS), since the model assumes bubble-PFAS equilibrium is achieved, although bubble lifetimes are insufficient for equilibration [133], [134]. Further, the model was accurate for PFOS but not PFOA in competitive PFOX systems and exaggerated $R_{{PFOA}_{Max}}$ by ten fold [45]. Short chain PFSAs and PFCAs are degraded more slowly due to lower K_eq_ values [43], which is not accounted for in Eqs. (14), (15), (16), (17). Hence, matching of LH or MM kinetics to measured ultrasonic PFAS data limits understanding of the reactions. Further, LH and MM kinetics only accurately describe (sonochemical) reactions which are either zeroth [45] or first order [45], [135], [136]. While only two papers (to date) have attempted to fit kinetic models to PFAS sonolysis, 22 others have simply fitted zero or pseudo-first order rate constants to experimental data and achieved good fits (R^2^ values of 0.70 – 0.99) [36], [37], [38], [39], [40], [41], [42], [43], [44], [47], [51], [52], [58], [60], [61], [62], [63], [64], [68], [94]. It therefore appears more beneficial to develop semi-empirical rate equations based on zero or pseudo-first order kinetics and account for the ultrasonic and chemical properties used, rather than attempt theoretical model or curve fitting. The effects of the aforementioned sonochemical properties are discussed in the following section.

### 3.5 Effects of sonochemical conditions

Due to their interconnected and dynamic nature, it can be difficult to identify and isolate key ultrasonic parameters affecting PFAS sonolysis. Several parameters which have been investigated to date are reviewed here and a *meta*-analysis provided to bring insight on their relative effects on reaction mechanisms, rate, order, and products, as well as the optimum values for PFOX degradation rates.

#### 3.5.1 Frequency

The frequency (ν) applied in sonochemical systems can be used to partially control; the lifetime, oscillation rate, size distribution, number, symmetry and collapse intensity of cavitation events, as well as the ratios of standing/travelling waves and production of radical species [55], [57], [68], [137], [138]. Lower ultrasonic frequencies (20 – 100 kHz) [42], [81] generate large bubbles, higher individual collapse temperatures and hence are typically utilised for physical processes. Mid-high range frequencies (100 – 1,000 kHz) [81] show strong chemical degradation effects due to higher cavity populations, production of radicals and surface availability. However, chemical degradation rates reduce above 1,000 kHz [125]. Comparing reaction rates at mid-high frequencies (0.002 – 0.04 min^−1^) [45], [36], [37] and low frequencies (0.0002–0.03 min^−1^) [85], [60], [61] shows that lower frequencies are generally less effective at degrading PFAS, compared to high frequencies, particularly without use of radical generating reagents [85]. Hence, a balance of high collapse intensity and cavity population is required for environmental remediation [41], [57], [125]. Thus, this section will predominantly focus on frequency effects above 100 kHz, while Section 3.5.6 concerns low frequency treatment.

Table 3 shows a range of works which have investigated PFAS sonolysis and the frequencies at which they were conducted. Note this table does not contain all results from all relevant works, instead it focuses on works which have investigated multiple frequencies. The table also shows several other commonly reported reaction conditions, which should demonstrate the aforementioned complexity in comparing works from different authors, where multiple parameters and reported reaction metrics differ. It should also indicate the interconnected nature of these parameters, where differing conditions achieve similar rates. Note that the data are placed in order of the PFAS molecule(s) studied in the first column, then increasing frequency in the second.

**Table 3:** Conditions and reaction rates in studies on PFAS sonolysis under differing frequencies.

PFAS	Ultrasonic Parameters				PFAS Rate constant		F^-^ rate constant		Ref
	ν (kHz)	C_0_ (mg L^-1^)	PD (W L^-1^)	Dissolved gas	0^th^ order (nM min^-1^)	Pseudo-1^st^ order (10^–3^ min^-1^)	0^th^ order (µM min^-1^)	Pseudo-1^st^ order (×10^-3^ min^-1^)	
PFOA	20	20.0	6,000	Air	–	–	0.38	–	[59]
	20	20 + 2702.2 K_2_S_2_O_8_	6,000	Air	–	–	0.67	–	[59]
	40	49.7 + Sol-gel TiO_2_	166.7 + 5.3 UV light	O2	–	2.20	–	0.00	[60]
	40	50.0	150	Air	–	1.20	–	–	[58]
	40	70.4	500	N_2_	–	9.20	–	–	[62]
	40	70.4 + 0.03 IO_4_	500	N_2_	–	22.2	–	–	[62]
	40	50.0 + 2520 NaHCO_3_	150	N_2_	–	24.0	–	–	[58]
	200	10.0	3,333	Air	–	15.5	–	–	[37]
	200	10.0	3,333	Ar	–	32.0	–	–	[37]
	354	7.11	250	Ar	39.0	18.4	–	–	[45]
	354	0.103	250	Ar	–	41.0	–	–	[39]
	354	95.3	250	Ar	1,022.0	–	–	–	[45]
	618	4.97	250	Ar	–	36.0	–	0.30	[39]
PFOS	25	5.00	–	Ar	–	–	0.10	–	[42]
	44	10.0	100	Air	0.0	0.00	0.00	0.00	[36]
	200	10.0	3,333	Ar	–	16.0	–	–	[37]
	200	10.0	3333	Air	–	6.8	–	–	[37]
	354	1.04	250	Ar	–	28.0	–	–	[45]
	354	106	250	Ar	1,150.0	–	–	–	[45]
	354	0.104	250	Ar	–	27.0	–	–	[39]
	400	10.0	100	Air	133.0	1.30	2.10	1.40	[36]
	500	5.00	–	Ar	–	–	0.70	–	[42]
	500	50.0	–	Ar	–	–	3.60	–	[42]
	500	260	–	Ar	–	–	7.00	–	[42]
	500	10.0	100	Air	108.0	1.30	1.90		[36]
	1000	5.00	–	Ar	–	–	0.90	0.00	[42]
	1000	50.0	–	Ar	–	–	0.10	–	[42]
	1000	230	–	Ar	–	0.00	0.00	–	[42]
	618	5.38	250	Ar	–	16.0	–	–	[39]
	1000	10.0	100	Air	106.0	6.80	–	–	[36]
PFOA & PFOS	354	0.10 & 0.10	250	Ar	–	0.047 & 0.024	–	–	[46]
	612	0.10 & 0.10	250	Ar	–	0.008 & 0.008	–	–	[46]
	358	(4.11 & 5.00) ×10^−5^	333	Ar	–	0.057 & 0.040	–	–	[43]
	610	(4.11 & 5.00) ×10^−5^	333	Ar	–	0.043 & 0.029	–	–	[43]
	202	(4.11 & 5.00) ×10^−5^	250	Ar	–	0.020 & 0.010	–	–	[43]
	20 + 202	(4.11 & 5.00) ×10^−5^	250	Ar	–	0.027 & 0.013	–	–	[43]
	610	(4.11 & 5.00) ×10^−5^	250	Ar	–	0.034 & 0.020	–	–	[43]
	20 + 610	(4.11 & 5.00) ×10^−5^	250	Ar	–	0.037 & 0.021	–	–	[43]
PFHA & PFHS	202	0.117 & 0.092	250	Ar	–	0.019 & 0.012	–	–	[41]
	358	0.117 & 0.092	250	Ar	–	0.039 & 0.030	–	–	[41]
	610	0.117 & 0.092	250	Ar	–	0.036 & 0.022	–	–	[41]
	202	0.101 & 0.092	333	Ar	–	0.025 & 0.016	–	–	[43]
	610	0.101 & 0.092	250	Ar	–	0.036 & 0.022	–	–	[43]
	610	0.101 & 0.092	333	Ar	–	0.034 & 0.027	–	–	[43]
	1060	0.117 & 0.092	250	Ar	–	0.022 & 0.012	–	–	[41]
PFBA & PFBS	202	0.101 & 0.090	250	Ar	–	0.007 & 0.013	–	–	[41]
	358	0.101 & 0.090	250	Ar	–	0.012 & 0.018	–	–	[41]
	610	0.101 & 0.090	250	Ar	–	0.017 & 0.023	–	–	[41]
	1060	0.101 & 0.090	250	Ar	–	0.008 & 0.009	–	–	[41]
	202	0.101 & 0.090	250	Ar	–	0.007 & 0.013	–	–	[43]
	610	0.101 & 0.090	250	Ar	–	0.017 & 0.017	–	–	[43]
	610	0.101 & 0.090	333	Ar	–	0.021 & 0.021	–	–	[43]

- Indicates data not reported or calculable.

**Fig. 4:**
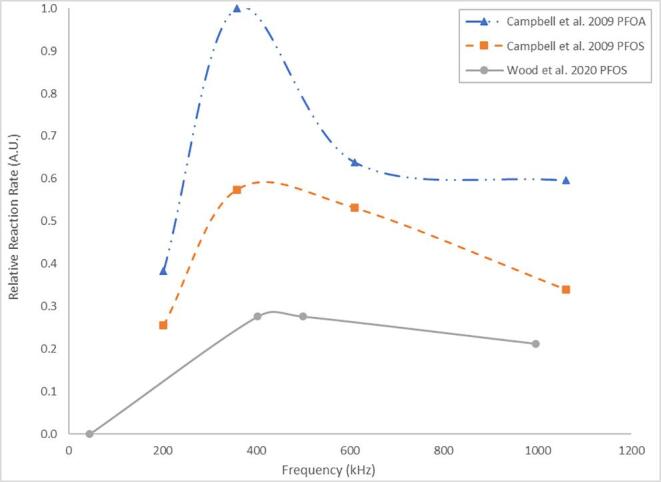
Comparison of frequency effects on the relative reaction rate (scaled to the fastest rate observed in all three experiments) for two works on PFOA and PFOS (Campbell et al. 2009 [41]) and PFOS (Wood et al. 2020 [36]) sonolysis at constant applied power.

For PFOA and PFOS, Table 3 shows that rates are typically higher under mid-high frequencies [39], [45] than low frequencies [36], [58], except in cases where radical additives are used to enhance PFOA degradation [58]. It also shows the lack of works on low-frequency PFOS sonolysis [36], which suggests it may be somewhat ineffective. In prior work, degradation rates of PFOA, PFOS, PFHxA and PFHxS at given frequencies (r_freq_) followed the order: r_358 kHz_ > r_619 kHz_ > r_1,060 kHz_ > r_202 kHz_ [41], [43], at constant applied power. The optimum frequency (358 kHz) was attributed to generating the greatest number of active bubbles by balancing bubble formation and collapse [41], [43], as was suggested for the optimal degradation of 1–4 dioxane at 358 kHz [125]. Other work agreed, showing that PFOS degradation followed the order r_402.6 kHz_ > r_500 kHz_ > r_996.1 kHz_ ≫ r_44 kHz_ [36]. Comparing these works [36], [41] suggests frequency effects depend on the target species, since there is a steeper initial decline in reaction rate after 358 kHz for PFOA than PFOS [41] (Fig. 4).

As shown in Table 3, PFHxA and PFHxS had similar pseudo-first order rate constants to PFOA and PFOS (when sonicated together) and the same optimal frequency [41]. However, PFBA and PFBS (PFBX) were optimally degraded at 610 kHz, which was attributed to the reduced hydrophobicity of shorter chains. Thus, shorter PFAS chains require more rapid oscillations, as well as the greater number and higher surface area of smaller bubbles generated at higher frequencies, which move the bubble toward the PFAS and enhance PFBX uptake [41], [43]. This is consistent with several other sono-degradation works, in which hydrophobic compounds (comparable to long chain PFAS) such as bisphenol A [82], alachlor [83] and methyl *tert*-butyl ether [84], were degraded well at 300 – 358 kHz, while hydrophilic compounds (comparable to short chain PFAS), such as carbon tetrachloride [80] and trichloroethane [81] were best degraded at higher frequencies (618 – 800 kHz). Another explanation is that higher frequencies enhance generation of radicals, such as hydroxyl (OH∙) [49], [80]. However, for PFOA and PFOS, radicals had little effect on degradation rates [37]. The slower reaction rates at 202 kHz and 1,000 kHz may partially explain the higher short chain formation [37], [42] at these frequencies compared to 358 – 575 kHz [36], [38], [39]. i.e., slower reaction rates might allow short chains to be formed and less readily degraded.

Two other works studying pure PFOS solution [42] and PFAS in diluted AFFFs [49] disagree with the trends in Fig. 4 since, at 1,000 kHz, greater fluoride release and TOC removal were observed compared to 500 kHz (PFAS concentrations not directly measured). The difference was attributed to increased bubble and radical formation at higher frequencies [49], however differing reactor volumes and geometries were applied between frequencies (Table 4). The power intensity for each transducer was identical (8 W cm^−2^) [42], [49] but, due to the higher volume, the power density (W L^−1^) would be lower in the 25/500 kHz reactor, thus affecting degradation potential [43], [68]. The differences in liquid height, reactor geometry and transducer mounting will have also affected sonochemical efficiency by changing the position, clustering and collapse intensity of cavitation bubbles [139], [140] as well as attenuation of the ultrasound due to differences in cavitation bubble location [140]. Hence, a fair comparison can only be made between frequency effects of 25 kHz and 500 kHz in the PFOS study, where (as shown in Table 4) fluoride release was ≈14.9 faster at 500 kHz than at 25 kHz [42]. This trend is in general agreement with previous findings on low frequency PFOS sonication [36], [41] (Table 3). Similarly, another work examined the sonication of a mixture of PFOA and PFOS whilst varying both power density (between 30.0 and 68.2 W L^−1^) and frequency (575 – 1,140 kHz) [68]. As shown in Table 4, the power densities used were not consistent across the frequencies studied, hence it is difficult to draw any parametric trend from the data. These works [42], [49], [68] demonstrate the interconnected nature of frequency and other parameters such as power density and PFAS structure, as suggested previously [41], [43]. Further, comparison between such works is difficult due to differences in initial concentrations used which were roughly double [43] or triple [41] those in previous studies [39], [42].

**Table 4:** Conditions used in frequency studies with variable frequency and power density.

PFAS/AFFF brand	Ultrasonic Parameters						0^th^ order F^-^ rate constant (µM min^-1^)		Ref
	ν (kHz)	C_0_ (mg L^-1^)	Reactor volume (L)	Reactor shape	Transducer mounting	Dissolved gas			
PFOS	25	5.0	12.0	Cubic	Sidewall	Argon	0.061		[42]
	500	5.0	12.0	Cubic	Sidewall	Argon	0.728		[42]
	1,000	5.0	4.50	Cylindrical	Base	Argon	0.906		[42]
3M	500	930	12.0	Cubic	Sidewall	Argon	0.430		[49]
	1,000	930	4.50	Cylindrical	Base	Argon	1.50		[49]
Ansul	500	930	12.0	Cubic	Sidewall	Argon	0.410		[49]
	1,000	930	4.50	Cylindrical	Base	Argon	2.17		[49]

PFAS	ν (kHz)	C0 (mg L^-1^)	PD (W L^-1^)	Reactor volume (L)	Initial pH	Dissolved gas	0th order rate constant (nM min^-1^)		Ref

PFOA	PFOS

PFOA & PFOS	575	0.0476 & 0.0600	30.0	0.5	6.02	Air	0.570	0.280	[68]
	860	0.0476 & 0.0600	50.0	0.5	6.02	Air	0.430	0.240	[68]
	1,140	0.0476 & 0.0600	62.8	0.5	6.02	Air	0.440	0.360	[68]

PFAS	ν (kHz)	C_0_ (mg L^−1^)	PD (W L^−1^)	Reactor volume (L)	Initial pH	Dissolved gas	Pseudo-1st order rate constant (×10^−3^ min^−1^)		Ref
							PFOA	PFOS	

PFOA & PFOS	575	0.0422 & 0.0565	77.0	0.2	6.02	Air	23.5	6.00	[68]
	860	0.0422 & 0.0565	113	0.2	6.02	Air	23.0	7.00	[68]
	1,140	0.0422 & 0.0565	148	0.2	6.02	Air	22.5	6.50	[68]

Multi-frequency (dual frequency) PFAS sonolysis has also been tested in two works to date [43], [50]. As shown in Table 5, pseudo-first order rate constants for PFOA and PFOS at 202 kHz were enhanced by 23 % (PFOA) and 12 % (PFOS) by the addition of the 20 kHz horn [43]. However, combined frequencies of 610 kHz + 20 kHz and of 202 kHz + 20 kHz showed no better degradation than at 610 kHz alone for PFOX, PFHX and PFBX [43]. The combination of 20 and 202 kHz was theorised to enhance the rate at 202 kHz (but not 610 kHz) since 20 kHz is closer to a multiple of 202 than 610, thus providing better constructive wave interference which reduces collapse time, possibly by enhanced rarefaction rate [43]. No results were given for using 20 kHz in isolation. Constructive waves can have greater amplitude than their constituent waves [141], thus dual frequency sonication can generate a greater size, number [140], and collapse intensity [142] per unit of power [51]. However, in this particular study, it offered no benefit compared to a single frequency [43]. Dual frequency (and multi-transducer) effects using a combination of 500 kHz and 1,000 kHz enhanced PFOS defluorination rates by 6.7 %, but enhanced sulphate removal rates by 68.4 %, compared to 1,000 kHz alone [53] (Table 5). The same set-up also enhanced fluoride release rates, but reduced sulphate release rates, for the degradation of two AFFFs (3M and Ansul) [50], compared to the individually applied 1,000 kHz frequency (Table 5). TOC removal was comparable for the 3M foam but reduced for the Ansul foam under dual frequency, compared to single frequency [50]. Thus, the distribution of sonolysis products depends on the frequencies used and the material being treated. Note, however that the power density in these dual frequency cases was higher [43], [50].

**Table 5:** Conditions used and degradation rates in works investigating multi-frequency PFAS sonolysis.

PFAS	Ultrasonic Parameters				PFAS Rate constant	0^th^ order rate constant		Ref
	ν (kHz)	C_0_ (mg L^-1^)	PD (W L^-1^)	Dissolved gas	Pseudo-1st order (10^–3^ min^-1^)	F^-^ (µM min^-1^)	SO_4_^2-^ (µM min^-1^)	
PFOA & PFOS	202	(4.11 & 5.00) ×10^−5^	250	Ar	0.020 & 0.010	–	–	[43]
	20 + 202	(4.11 & 5.00) ×10^−5^	250	Ar	0.027 & 0.013	–	–	[43]
	358	(4.11 & 5.00) ×10^−5^	333	Ar	0.057 & 0.040	–	–	[43]
	610	(4.11 & 5.00) ×10^−5^	250	Ar	0.034 & 0.020	–	–	[43]
	610	(4.11 & 5.00) ×10^−5^	333	Ar	0.043 & 0.029	–	–	[43]
	20 + 610	(4.11 & 5.00) ×10^−5^	250	Ar	0.037 & 0.021	–	–	[43]
PFOS	9x 1000	1300	109	Ar	–	0.30	0.19	[53]
	3x 500 + 9x 1000	1300	132	Ar	–	0.32	0.32	[53]

AFFF brand	Ultrasonic Parameters				PFAS Rate constant	0^th^ order rate constant		Ref
	ν (kHz)	AFFF dilution ratio	PD (W L^−1^)	Dissolved gas	Pseudo-1^st^ order (10^−3^ min^−1^)	F^−^ (µM min^−1^)	SO_4_^2−^ (µM min^−1^)	

Ansul	9x 1000	25	109	Ar	–	0.0441	0.223	[50]
	3x 500 + 9x 1000	25	132	Ar	–	0.0751	0.163	[50]
3 M	9x 1000	25	109	Ar	–	0.183	0.257	[50]
	3x 500 + 9x 1000	25	132	Ar	–	0.288	0.148	[50]

- Indicates data not reported or calculable.

The reviewed works in this section highlight that frequency is a critical parameter in the sonolysis of PFAS and that the optimum frequency depends on the specific PFAS to be treated. It has also been shown that power must be carefully controlled to compare frequency effects. The effects of power variations will be discussed in the next section.

#### 3.5.2 Power

In previous work, PFOA, PFOS, PFHxA and PFHxS degradation rates showed a near-linear response to increasing power density (83 – 330 W L^−1^) at frequencies of 202, 358 and 610 kHz [43] (Table 6). However, PFBX at 202 kHz and PFHxA at 610 kHz showed a maximum rate, above which increased power reduced reaction rates, possibly due to bubble clustering/growth, which increases the average PFAS diffusion distance, especially for PFBX which already has low volatility [43]. Similarly, treatment of several PFAS in investigation derived waste was enhanced near-linearly by increasing ultrasonic power from 250 − 1,250 W L^−1^ (data not extractable), however, PFOA’s response was less linear, perfluorooctane sulphonamide (PFOSA) showed reduced rates above 500 W L^−1^ and PFBS’s rate also reduced between 1,250 – 5,500 W L^−1^ [52]. In other work, PFOA and PFOS reaction rates had a near-linear “S”-shaped response to increasing power densities from 30 − 262 W L^−1^ at 575 kHz [94], the precise shape of which was dependent on the choice of dissolved gas (air vs argon, less vs more linear). However, in said work, the starting PFAS concentration also varied up to 20 % at each power density, perhaps suggesting why the response was different to work using similar power densities at 610 kHz where reported starting concentrations were constant (Table 6) [43].

**Table 6:** Conditions and degradation rates in works investigating power effects on PFAS sonolysis. Data is ordered by increasing power density (PD, column 5).

PFAS	Ultrasonic Parameters				PFAS pseudo-1^st^ order rate constant						Ref
	C_0_ (mg L^-1^)	Dissolved gas	Volume (L)	PD (W L^-1^)	ν (kHz)						
					20	40	202	358	575*	610	
PFOA	0.035	Air	0.5	30.0	–	–	–	–	0.0025	–	[94]
	0.040	Air	0.3	52.0	–	–	–	–	0.0030	–	[94]
	54.7	–	0.5	60.0	–	0.0025	–	–	–	–	[47]
	0.043	Air	0.2	77.0	–	–	–	–	0.0100	–	[94]
	0.099	Ar	0.6	83.0	–	–	–	0.0063	–	0.0080	[43]
	0.042	Air	0.1	147	–	–	–	–	0.024	–	[94]
	0.099	Ar	0.6	166	–	–	–	0.0220	–	0.0230	[43]
	54.7	–	0.5	180	–	0.013	–	–	–	–	[47]
	0.099	Ar	0.6	250	–	–	–	0.0480	–	0.0340	[43]
	0.040	Air	0.05	262	–	–	–	–	0.086	–	[94]
	0.099	Ar	0.6	330	–	–	–	0.0570	–	0.0430	[43]
	7.5 ×10^−5^	–	0.1	375	0.075	–	–	–	–	–	[63]
	49.7	–	0.3	500	–	0.0313	–	–	–	–	[64]
PFOS	0.051	Air	0.5	30.0	–	–	–	–	0.0020	–	[94]
	0.055	Air	0.3	52.0	–	–	–	–	0.0025	–	[94]
	0.060	Air	0.2	77.0	–	–	–	–	0.0050	–	[94]
	0.100	Ar	0.6	83.0	–	–	–	0.0070	–	0.0050	[43]
	0.059	Air	0.2	147	–	–	–	–	0.037	–	[94]
	0.100	Ar	0.6	166	–	–	–	0.0170	–	0.0180	[43]
	0.100	Ar	0.6	250	–	–	–	0.0280	–	0.0220	[43]
	0.057	Air	0.2	262	–	–	–	–	0.068	–	[94]
	0.100	Ar	0.6	330	–	–	–	0.0400	–	0.0290	[43]
	5.5 ×10^−5^	–	0.1	375	0.068	–	–	–	–	–	[63]
PFHxA	0.100	Ar	0.6	83.0	–	–	0.0070	–	–	0.0100	[43]
	0.100	Ar	0.6	166	–	–	0.0160	–	–	0.0210	[43]
	0.100	Ar	0.6	250	–	–	0.0190	–	–	0.0360	[43]
	0.100	Ar	0.6	330	–	–	0.0250	–	–	0.0340	[43]
PFHxS	0.092	Ar	0.6	83.0	–	–	0.0050	–	–	0.0070	[43]
	0.092	Ar	0.6	166	–	–	0.0120	–	–	0.0140	[43]
	0.092	Ar	0.6	250	–	–	0.0120	–	–	0.0220	[43]
	0.092	Ar	0.6	330	–	–	0.0160	–	–	0.0270	[43]
PFBA	0.100	Ar	0.6	83.0	–	–	0.0044	–	–	0.0037	[43]
	0.100	Ar	0.6	166	–	–	0.0064	–	–	0.0061	[43]
	0.100	Ar	0.6	250	–	–	0.0072	–	–	0.0170	[43]
	0.100	Ar	0.6	330	–	–	0.0065	–	–	0.0210	[43]
PFBS	0.090	Ar	0.6	83.0	–	–	0.0048	–	–	0.0040	[43]
	0.090	Ar	0.6	166	–	–	0.0090	–	–	0.0060	[43]
	0.090	Ar	0.6	250	–	–	0.0130	–	–	0.0170	[43]
	0.090	Ar	0.6	330	–	–	0.0130	–	–	0.0210	[43]

*data estimated from graph.

- Indicates data not reported or calculable.

Nonlinear/inverse responses of PFAS degradation rates to increasing power were also observed during low frequency sonication. At 40 kHz with sulfate ion addition, PFOA degradation peaked at 500 W L^−1^, out of a maximum 1,667 W L^−1^ [64]. At 20 kHz ammonium-PFOA degradation with persulfate oxidation peaked at 6,000 W L^−1^ across the power density range 2,000 – 10,000 W L^−1^ [59]. Similarly, at 20 kHz, trace PFOS contamination was degraded without oxidant addition at 1,500 – 4,100 W L^−1^, with peak degradation at 3,750 W L^−1^ [63]. However, for the three aforementioned works, not all rate constant data was given at the different power settings. However, Table 6 compares these with those of the higher frequency works and shows that low frequencies require higher concentrations of PFAS or power densities to achieve similar pseudo-first order rate constants to the mid-high frequency studies.

The lack of agreement between works on the optimum power density for the degradation of various PFAS demonstrates that parameters such as frequency, reactor size/geometry, PFAS type/concentration, and oxidant type/concentration are interlinked with the effects of power [57]). Increasing ultrasonic power can enhance sonochemical degradation through greater number, size, and collapse temperature and pressure of active bubbles [57], [143], increased concentrations of radicals [137], [144], bulk mixing, and stabilisation of bubble populations at the wave antinodes [145]. However, beyond a certain threshold, increased power reduces reaction rates [146], [147], as large inactive bubbles; 1) agglomerate at the transducer surface or antinode regions, which attenuates the sound wave [59], [140], [148] (known as “decoupling”), 2) become inactive due to reduced collapse temperatures [149], and/or 3) are pushed out of the antinode region [149]. High power also limits the maximum bubble size for active cavitation [57] and causes large bubbles to degas from solution [57], [143]. An initially linear relationship between sonochemical effects and power which eventually plateaus and then decreases at high powers has been observed for both sonoluminescence and sonochemiluminescence systems, in non-PFAS related work [143], [149]. Sonochemiluminescence has been correlated with the sonolytic degradation of PFOS [36] and so may follow a similar trend. The near universal observation of plateau, linear and then decreasing responses of sonochemical reaction rates to increasing power suggest that those works which did not observe them for PFAS degradation were likely not completed at low/high enough power densities to observe the plateau/decline regions [120], [143], [149], [150].

At high powers and low frequencies, bubbles are larger in size [138] (partly due to secondary Bjerknes forces) [57] and more populous in the antinode regions [145]. Thus, a contributing factor to non-linear power effects may be bubble ejection from the sonochemically active antinode regions due to primary Bjerknes forces and subsequent degassing [149]. Secondary Bjerknes forces also contribute to repulsion between different sized bubbles and coalescence between similarly sized bubbles due to their respective oscillation rates which may lead to degassing and growth to inactive sizes [57], with repulsive forces more likely in low frequencies due to more varied distribution in bubble size [138]. Of the works reviewed in this section, mid-high frequency (100 – 1,000 kHz) [43] peak PFAS reaction rates are achieved at much lower powers (200–300 W L^−1^) compared to low frequencies (20–100 kHz) (500 – 3,750 W L^−1^) [59], [63], [64]. This matches observations of decoupling limits, which were lower for higher frequencies [150], [151], hence non-linear power effects at higher frequencies may be due to decoupling. Since non-linear responses of reaction rate to power exist [43], [59], [68], [63], [64], the efficiency of treatments augmented by increased power should be considered, in addition to the reaction rate.

#### 3.5.3 Concentration

PFAS sonolysis has been reported at concentrations ranging from 20 pM [63] to 5.3 mM [53], with most investigations at ≈40 µM [37], [38], [41], [42], [43], [46], [49], [58], [60], [61], [62], [64], [65]. At lower concentrations, PFOX degradation proceeds via pseudo-first order kinetics [37], [39], [41]. At higher concentrations, however, PFAS saturation of the bubble surface limits the number of molecules degraded per cavitation event, making the reaction rate independent of concentration (zero order) [45]. This has been observed for various PFAS, sample matrices, and ultrasonic frequencies [38], [42], [44], [48], [63]. Some authors distinguish between three kinetic regimes: first order, pseudo-first order and zero order. The kinetic transition concentration may depend on several factors. The surface area available during bubble growth determines the number of PFAS molecules participating in the reaction [37]. Bubble surface area is dependent on the size and number of available active bubbles, which is controlled by factors including: ultrasonic frequency, power, surface tension (caused by surfactant concentration, i.e. [PFAS], and co-contaminants) and the type and concentration of dissolved gas used [57]. Degradation rates are also limited by the number of adsorbed molecules and hence the starting PFAS concentration and the PFAS partitioning coefficient [45]. Power density may also impact the PFAS reaction rate sufficiently to alter the reaction order at frequencies from 575 kHz to 1,140 kHz [68]. Table 7 compares key ultrasonic conditions in works where a PFOX sonolysis kinetic transition concentration was observed or derived from data available. For other PFAS, kinetic transition concentrations have not yet been reported, however, sonolysis of diluted AFFFs suggests that transition concentrations are lower for shorter chains (≈ 2 μM for PFBS) [48].

**Table 7:** Comparison of the PFAS kinetic transition concentration in known sonolytic works.

PFAS	Ultrasonic Parameters			Kinetic transition concentration (μM)	Ref
	Frequency (kHz)	Power Density (W L^-1^)	Dissolved gas choice (-)		
Na-PFOS	354	250	Argon	39.0	[45]
NH_4_-PFOA	354	250	Argon	30.5	[45]
PFOS in FC-600 AFFF	505	188	Argon	14.6 – 29.2*	[48]
PFOA & K-PFOS	575	77^+^	Air	23.6 29.5	[38]
PFOA & K-PFOS	20	1,500 – 3,750	Air^^^	0.0004 0.0006	[63]
K-PFOS	400 – 1,000	200	Air	2.30 – 2.90	[36]

*Calculated based on 1 in 250 – 500 dilution of 7.3 mM PFOS in initial FC-600 AFFF.

^+^Calorimetrically measured.

^^^Assume since not stated.

Transition concentrations for both PFOA and PFOS in Table 7 are consistently within the range of 15 µM – 40 µM, at mid-high frequencies [45], with one notable exception (2.3 – 2.9 μM [36]). Where PFOS and PFOA were degraded under identical conditions, PFOS consistently showed a 25 – 50 % higher transition concentration than PFOA (ignoring differences in the molecule’s respective cations) [38], [45], [63]. This may be due to PFOS having greater surface activity [39] at the bubble interface, as well as greater surface tension reduction, allowing greater PFOS concentrations at the bubble interface. The greater hydrophobicity overcomes PFAS-PFAS repulsion (which would otherwise push molecules back into the liquid phase) and allows an increased number (nucleation) [152] and size (reduced resistance to growth) [153] of bubbles. The PFOX transition concentration was several orders of magnitude lower at 20 kHz [63], despite the much higher power used, indicating frequency dependence. This may be due to lower levels of chemical activity at lower frequencies [154], leading to reduced reaction rates or an alternative physical degradation route. Comparing pure PFAS in water [45] with those in AFFFs [48] indicates that co-contaminants may slightly reduce the kinetic transition concentration by competitive bubble interface adsorption, and/or competitively reacting with radicals generated by ultrasound [50]. The slightly higher frequency and lower power used in the AFFF study may have also contributed to the lower transition concentration, by reducing the bubble size and hence available interfacial area, as discussed in 3.5.1, 3.5.2. Differences in dissolved gas mainly control heating inside the bubble after sorption [37], as discussed in Section 3.5.4, but also affect gas concentration due to differing solubilities, and hence affects bubble size distributions and, therefore, interfacial areas [155]. However, the effect of this seems limited, as shown in Table 7, where similar kinetic transition concentrations are seen in air and argon saturated systems [38], [45], [63]. The rate order and kinetic transition concentration may also be different for reactants and products. For example, when K-PFOA and PFOS were sonicated simultaneously, PFAS degradation was pseudo first order while fluoride release was zero order [38]. This may be due to the number of fluoride molecules per PFAS, hence fluoride was still produced after PFAS destruction due to partial defluorination of the initial species.

**Table 8:** Comparison of sonolysis conditions and optimum dilution factors applied to AFFFs.

AFFF brand	Dilution range tested	Frequency (kHz)	Optimum dilution factor				Ref
			(PFAS removal)	(TOC removal)	(F^−^ release)	(SO_4_^2−^ release)	
FC-600	250 – 50,000 ×	505	5,000 ×	–	–	–	[48]
3M	200 – 929.4 ×	1,000	–	200 ×	200 ×	–	[49]
Ansul	200 – 929.4 ×	1,000	–	200 ×	200 ×	–	[49]
3M	10 – 500 ×	500 + 1,000	–	500 ×	10 ×	25 ×	[50]
Ansul	25 – 900 ×	500 + 1,000	–	500 – 900 ×	25 – 100 ×	100 ×	[50]*

- Indicates data not reported.

*Optimum value changes over time.

There is some evidence of high concentrations having a negative impact on PFOX degradation rates at 1 MHz [42] and at 20 kHz [63]. At similar micro-molar concentrations, other organics are suggested to enter the bubble and quench the collapse temperature below the required pyrolysis temperature [131]. However, this should not be possible for surfactants such as PFAS, which prefer the interfacial region and barely penetrate into the bubble core (Section 3.1). The explanation proposed was that PFAS reduce gaseous rectified diffusion into bubbles, based on nitrite measurements [38], which may limit bubble growth. PFAS are anionic surfactants, so comparison to other anionic surfactants, such as sodium dodecyl sulfate (SDS), may predict their behaviour. Rectified diffusion is reduced by SDS, however, the effect is inferior to the effects of reduced bubble coalescence, which generates more sonochemically active bubbles [156]. This suggests that reduced rectified diffusion may not be solely responsible for the negative effects of high PFAS concentrations [42], [63].

AFFF sonolysis works utilise diluted AFFFs, likely due to viscosity [157] or foaming [158] effects which limit active bubble formation and ultrasonic testing. While one work demonstrated a linear correlation between dilution factor and F^−^ release rate [49], others showed nonlinear effects, with maximum reaction rates observed at 5,000x dilution [48], 100x and even 10x (with inversely proportional dilution effects) [50] (Table 8).

Each study in Table 8 uses a different metric for reaction completeness; hence the optimum dilution factor must be considered with respect to a given reaction rate. The relative effects of dilution on fluoride/sulphate release and TOC removal appear dependent on foam brand, likely due to their differing compositions [50]. The existence of an optimal dilution factor which is not at either extreme of the above tested ranges suggests competing physical effects of decreasing viscosity with increasing dilution (which facilitates cavity formation and growth) [157] and decreasing PFAS concentration (which reduces reaction rates) [37], [39], [41]. One work showed optimum dilution of 200x for both 3M Lightwater and Ansul TOC removal and defluorination [49], whereas another showed optimum Lightwater TOC removal and defluorination at 500x and 10x dilution, respectively, and optimum Ansul TOC removal and defluorination at 500 - 900x and 100x dilution, respectively [50]. The lack of consistency in findings between AFFF works is considerable compared to the near-consistent switch concentrations of pure PFAS solutions, regardless of frequency, power density or dissolved gas choice (Table 7) [36], [38], [45], [48]. AFFFs have more complex formulations and thus will demonstrate several competing effects as different PFAS, as other components break down and interact with the ultrasonic bubbles. Further, several different ultrasonic conditions were tested in each work including mid, high, and dual mid-high frequencies. Therefore, significant differences between works are less surprising.

#### 3.5.4 Dissolved gas

Degradation rates of PFOX under argon were consistently greater than under air, which was attributed to argon’s higher polytropic index (γ) [37], [47], [61], [62], which affects cavity collapse temperatures [57], [157]. Argon also reduced PFOA formation during PFOS sonolysis [37], likely due to the higher collapse temperatures allowing more complete and faster PFOS degradation. However, two mid-high frequency works reported alternative findings. In one work [68], gases were sparged or not sparged and rates of PFOA degradation followed the order r_Air,no sparging_ > r_Helium sparging_ > r_Nitrogen sparging_ > r_Argon sparging_ > r_Oxygen sparging_ > r_Ozone sparging_ and those of PFOS followed the order r_Air, no sparging_ > r_Helium sparging_ > r_Argon sparging_ > r_Oxygen sparging_ > r_Nitrogen sparging_ > r_Ozone sparging_ [68] (values not extractable). No tests were done for air sparging, which suggests that mechanical sparging may influence the effects of the chosen gas species (and reduce reaction rates). However, the original authors attributed these observations to the formation of unmeasured organic and inorganic species [38], [159], which are dependent on the dissolved gas choice and compete for the bubble interface [68]. Another work investigated the effects of increasing power density on PFOX degradation under air and argon [94]. However, the PFAS concentrations were not held constant as the gas and power conditions were varied, hence, no rate comparison table is presented.

Sonochemical effects of differing dissolved gases on PFAS reaction rates at low frequencies may also relate to chemical effects (in addition to thermal effects) [38]. PFOA’s reaction rate reduced under oxygen and increased under argon (compared to air) at 40 kHz with permanganate oxidation (Table 9) [47]. Rate enhancement by argon was attributed to increased collapse temperature and radical production rate [47]. Similarly, when using N_2_, O_2_ or air during PFOA degradation at 40 kHz with iodide oxidation, rate constants followed the order N_2_ > Air > O_2_ for both PFOA decomposition (Table 9) and fluoride release. Similar reaction rates should be expected here due to the similar γ values of these gases (1.403, 1.400, and 1.397, respectively at 20 °C) and the composition of air being mainly N_2_ and O2 [47], [62]. However, the relative reaction rates vary more considerably than the γ values, suggesting some non-linear effects with increasing polytropic index. One explanation was that increasing dissolved O_2_ reduced the degradation rate by limiting IO_3_ production in favour of less affective IO_4_^-^ radicals [62]. Argon may be less suitable than air for shorter chain perfluoro-ether by-products, since the NFDOHA degradation rate constant at 28 kHz, was increased by ∼16 % under argon, however, fluoride release was reduced by ∼11 % [61], likely due to reduced radical production and enhanced thermal degradation. Air saturated sonolysis systems also generate ionic products including nitrites and subsequently nitrates [38] which can lead to pH changes by the formation of nitrous acid [38], [159]. When degrading PFOA with CO_3_ radicals at 40 kHz, it was postulated that N_2_ enhanced OH formation and hence radical degradation, however, nitrogen sparging showed limited effect [69].

**Table 9:** Conditions used and degradation rates in low frequency works investigating dissolved gas effects on PFOA sonolysis.

PFAS	Ultrasonic Parameters				PFAS pseudo-1^st^ order rate constant				Ref
	C_0_ (mg L^-1^)	ν (kHz)	Volume (L)	PD (W L^-1^)	Dissolved gas (polytropic index)				
					O_2_ (1.397)	Air (1.400)	N_2_ (1.403)	Ar (1.670)	
PFOA	54.7 + (895 MnO_4_^−^)	40	0.5	180	0.0095	0.013	–	0.014	[47]
	70.4 + (895 IO_4_^−^)	40	0.3	500	0.0037	0.0053	0.0092	–	[62]

- Indicates data not reported.

While numerous authors use argon as the gas phase of choice due to its rate enhancing effects [37], [39], [42], [43], [45], [46], [48], the cost of using argon has not been compared with air, which could prove crucial for industrial scale treatment.

#### 3.5.5 PFAS structure

Here, structural effects of the PFAS molecule, including headgroup, degree of fluorination, and chain length, are considered with respect to partitioning coefficients, surface tension effects, reaction mechanisms and sonolysis rates. Then, the relative impacts of individual structural factors are assessed.

##### 3.5.5.1 Headgroup

PFOA degrades faster than PFOS at frequencies from 200 to 1,060 kHz [38], [39], [41], [44] and under dual low-frequency sonication (20 + 43 kHz) [51]. Likewise, PFHA degrades faster than PFHS across frequencies 200 to 1,060 kHz [41], [44]. At 28 kHz, addition of S_2_O_8_^2–^ enhanced the degradation rate of PFECs but not PFESs [61]. Previous explanations for these observations state that a higher bubble collapse temperature is required to cleave the C-S bond in the C-SO_3_H group, due to its increased activation energy (≈355 kJ mol^−1^[95], [96]), compared to the C—C bond found in the C-COOH group of PFCAs/PFECs (≈348 kJ mol^−1^[21]) [39]. While generally agreed by several authors [36], [41], [70], [94], measurements of C—C and C—S bond strengths shows significant variation and their effects on required bubble collapse temperature are yet to be measured. An alternative possibility is that the SO_3_H group, being larger in size than the COOH group, instead provides greater shielding of the headgroup-tail bond from thermal, radical or electron attack. Several other PFAS headgroups exist, such as alcohol and phosphoric acid [19], however, their effect on degradation rate has not been studied to date. Fernandez et al. studied the structural effects of several different PFAS on sonolysis rate [44], as summarised and compared with other works in Table 10. As can be seen, PFCAs are degraded faster than PFSAs of comparable carbon chain length.

**Table 10:** Conditions used and degradation rates in works investigating PFAS structural effects on PFAS sonolysis.

Substance	Ultrasonic Parameters				Structural parameters			Rate constant		Ref
	C_0_ (mg L^-1^)	ν (kHz)	V (L)	PD (W L^-1^)	Acid head group	Perfluoro chain length	Carbon chain length	Pseudo-1^st^ order PFAS (10^–3^ min^-1^)	Zero order F^-^ (μM min^-1^)	
PFEES	59.7	500	12.2	8 (W cm^−2^)	Sulphonic	4	4	–	3.9	[44]
PFBS	45.6	500	12.2	8 (W cm^−2^)	Sulphonic	4	4	–	1.8	[44]
PFHxS	56.1	500	12.2	8 (W cm^−2^)	Sulphonic	6	6	–	2.6	[44]
6:2 FTS	56.0	500	12.2	8 (W cm^−2^)	Sulphonic	6	8	–	1.5	[44]
PFOS	50.0	500	12.2	8 (W cm^−2^)	Sulphonic	8	8	–	3.5	[44]
PFOS	10.0	200	0.06	3,333	Sulphonic	8	8	16	–	[37]
OS^	10.0	200	0.06	3,333	Sulphonic	0	8	220	–	[37]
PFPrA	55.4	500	12.2	8 (W cm^−2^)	Carboxylic	2	3	–	1.6	[44]
PFPeA	49.7	500	12.2	8 (W cm^−2^)	Carboxylic	4	5	–	2.5	[44]
PFHxA	48.2	500	12.2	8 (W cm^−2^)	Carboxylic	5	6	–	3.5	[44]
PFOA	46.8	500	12.2	8 (W cm^−2^)	Carboxylic	7	8	–	3.7	[44]
PFOA	10.0	200	0.06	3,333	Carboxylic	7	8	32	–	[37]
OA*	10.0	200	0.06	3,333	Carboxylic	0	8	220	–	[37]
PFOA & PFOS	(4.11 & 5.00) ×10^−5^	358	0.60	333	Sulphonic & carboxylic	7 & 8	8 & 8	0.057 & 0.040	–	[43]
PFHA & PFHS	0.117 & 0.092	358	0.60	250	Sulphonic & carboxylic	5 & 6	6 & 6	0.039 & 0.030	–	[41]
PFBA & PFBS	0.101 & 0.090	619	0.60	333	Sulphonic & carboxylic	3 & 4	4 & 4	0.021 & 0.021	–	[41]

OS^ − 1-Octane sulphonic acid.

OA* – n-Octanoic acid.

- Indicates data not reported or calculable.

##### 3.5.5.2 Degree of fluorination and telomer groups

Similar truncation mechanisms were observed between PFOX and their hydrocarbons equivalents, n-octanoic acid and 1-octanesulfonic acid, at 200 kHz [37], suggesting that degree of fluorine does not significantly affect sonolytic reaction mechanisms. However, the hydrocarbon alkanoic acids degraded 15 and 32 times faster than their respective PFAS equivalents, PFOA and PFOS, and this was attributed to the high strength of the C-F bond and reduced PFOX volatility due to the presence of fluorine [37] (Table 10). It should also be noted that the smaller hydrogen atoms will provide reduced shielding to the carbon chain, compared to fluorine atoms [20], [21], which may increase susceptibility to radical, electron or thermal attack. Similarly, 6:2 FTS was defluorinated twice as fast as PFOS under 20 + 43 kHz [51]. By considering 6:2 FTS as a PFOS molecule with four fluorine atoms replaced by hydrogens, it is again seen that a reduced degree of fluorination increased the reaction rate. However, this is contradicted by the work summarised in Table 10, in which fluoride release of 6:2 FTS was ≈63% slower than that of PFOS, despite a higher initial 6:2 FTS concentration but comparable fluorine concentration [44]. The worked explained that 6:2 FTS fractures into shorter chain species which are less hydrophobic and slower to degrade, than PFOS [44]. However, in the same work, PFEES, which has an even lower degree of fluorination, was defluorinated slightly faster than PFOS or PFBS. PFBS has the same number of perfluorinated carbons but higher hydrophobicity than PFEES [44]. Both observations were likely due to PFEES’ C—O—C bond being much weaker than C—C and C—F bonds, leading PFEES’s susceptibility to radical attack [44] (Section 3.1). Therefore, radical susceptibility may dominate hydrophobic effects, especially in the presence of high concentration oxidative species. This structural effect may also explain the alternative truncation mechanism seen for PFAS with telomer ether groups [61], discussed in Section 2.4.

##### 3.5.5.3 Chain length

PFAS degradation rates increase with chain length, as observed from C3 - C8 [41], [66], [43], [44]. At 500 kHz, PFHxS and PFBS defluorination rates were 1.3 and 1.9 fold lower than PFOS, and those of PFHxA, PFPA, and PFPrA were 1.1, 1.8, and 2.3 fold lower than PFOA, respectively [44] (Table 10). Similarly, at 358 kHz, the rate of PFBS degradation was 1.6 fold slower than PFOS and PFBA was 3.3 times slower than PFOA [41]. These differences were attributed to the smaller compounds’ reduced affinity for the bubble interface, due to lower hydrophobicity (lower K_eq_) [41], as well as their reduced electron withdrawing effect in the perfluoro chain [20], [44]. This is supported by the need for faster bubble oscillations to degrade shorter chain PFAS, due to rate limitation by diffusion to the bubble surface [41], [45] (Section 3.3). Hydrophobicity was similarly implicated in the explanation for why, at 200 kHz and with the addition of UV light, PFPrA was defluorinated ≈5 faster by UV than by ultrasound, showing the opposite trend to PFOA [66]. However, long chain hydrophobicity may not be the only factor affecting degradation rate. Larger surfactant molecules can enhance acoustic (or micro-) streaming of fluid around ultrasonic bubbles [121]. Hence, the adsorption of the larger PFAS to the bubble interface may be accelerated by enhanced bulk liquid diffusion. Conversely, rate enhancement by persulphate ion addition was greatest for short chains under 28 kHz [61]. This was likely due to their already low reaction rates being enhanced by increased partitioning (salting out) effects [46] (3.3, 3.5.7).

##### 3.5.5.4 Surfactant chemistry

In addition to the relative strengths of C–C and C-S bonds, different surface tension effects of PFCAs and PFSAs may partially explain their different degradation rates [45]. Small increases in PFAS concentration can greatly reduce surface tension, while increasing the sonochemical degradation rate [45]. For PFOS, surfactant effects are observed at lower concentrations than PFOA, so the reduced rate of degradation of PFOS is not due to slow partitioning to the bubble interface [45]. However, it is difficult to de-couple the surface tension effects of a PFAS from its bonds strength effects on reaction rates. Further discussion on surfactant effects is included in Section 3.5.3.

##### 3.5.5.5 Overall structural effects

By comparing the effects of PFAS structural factors on their sonolysis rates, we determine here the relative dominance of each. In addition to PFCAs degrading faster than the PFSAs, PFHA degraded approximately twice as fast as PFOS at frequencies from 200 kHz to 1,060 kHz and 250 W L^−1^ power density [41], suggesting that the effect of chain length on hydrophobicity is less significant for degradation rates than the effect of bond strength. However, in one work, PFBS was degraded ≈69% faster than PFBA at 610 kHz [43] and from 87 to 333 W L^−1^. Hence, the relative effect of the higher partitioning coefficient (K_eq_), attributed to the SO_4_^2–^ headgroup, might outweigh that of its enhanced thermal stability in short chain lengths (≤C4), and vice-versa for longer chains (≥C5) [43]. Another factor is the surfactant surface density of PFCAs and PFSAs at the bubble surface, which differs due to chain length and the relative sizes (steric effects) of the COOH and SO_3_H headgroups (partial molar volumes 24.9 and 35.2 cm^3^ mol^−1^, respectively [160]). Despite having a larger head group, PFOS is a stronger surfactant than PFOA and has a greater surface concentration at the interfacial region, suggesting that hydrophobicity outweighs headgroup size effects on interfacial packing (Fig. 2) [41]. However, PFOA is degraded faster than PFOS [38], [39], [41], [44], suggesting that interfacial density effects (and the effects of PFOS as a stronger surfactant) on reaction rate are outweighed by bond strength attributes. For short chains, however, headgroup repulsion between molecules at the bubble surface dominates over hydrophobicity, hence, maximum bubble interface concentrations are reduced by 55% due to limited interfacial penetration [41]. This may also explain why PFBA was more slowly degraded than PFBS under comparable conditions [41], since it has a lower hydrophobicity and bubble-anchoring ability than PFBS, and cannot pack as densely at the bubble interface due to inter molecule repulsion.

One area lacking in analysis of structural effects on sonolysis rate are the different cations bound in PFAS salts. In works concerning PFOA and PFOS sonolysis, researchers have used PFOA, NH_4_-PFOA, PFOS, K-PFOS and Na-PFOS [38], [45], [48]. While dissolved cations make little difference to reaction rates of PFOX (≈±5%) [46], they may affect PFAS partitioning at low pH values. Further, differences in cations make comparison between works difficult and researchers should collectively unify the choice of cations.

#### 3.5.6 Effect of radical additives and low frequency mechanisms

PFAS degradation is typically slow at lower frequencies (20 – 100 kHz) [36], [65], attributed to the reduced bubble oscillation rate, number of bubbles, and, associated capture of PFAS [41], compared to higher frequencies. However, low frequency degradation rates can be enhanced using chemical or physical agents to generate oxidising radicals [64], [59], [60], [61], [62] (Table 11). Hence, degradation routes at low frequencies are likely dominated by radical-mediated mechanisms, rather than thermal degradation routes, and are specific to the oxidising techniques used. Most low frequency PFAS sonication research to date has focused on PFOA (Table 11), likely because PFOS resists radical attack.

**Table 11:** Summary of parameters used in works utilising radical mediated PFAS degradation.

PFAS	Oxidant	Radicals formed*	ν (kHz)	Power density (W L^-1^)	Degradation rate (min^-1^)	Ref
PFOA	KMnO_4_	OH	40	180	0.015	[47]
PFOA	Na_2_S_2_O_8_	OH &${\mathrm{SO}}_{4}^{-}$	20 & 43	550 & 250	0.370	[51]
PFECs	K_2_S_2_O_8_	${\mathrm{SO}}_{4}^{-}$	28	33.3	0.320	[61]
PFOA	TiO_2_ + 254 nm UV light	OH &$O_{2}^{-}$	40	167	0.350	[60]
PFOA	KIO_4_	${\mathrm{IO}}_{3}$	40	500	0.00022 – 0.0022	[62]
PFOA	NaHCO_3_	$CO_{3}^{-}$	40	150	0.0022	[58]
NH_4_-PFOA	K_2_S_2_O_8_	${\mathrm{SO}}_{4}^{-}$	20	3,000	Not given	[59]
PFOA	Na_2_SO_4_	${\mathrm{SO}}_{4}^{-}$	40	500	Not given	[64]

*Not measured.

##### 3.5.6.1 Radical enhanced sonolysis of PFOA

The radicals formed in the works shown in Table 11 are hypothesised by the authors of these works, since no work has reported direct measurement of radical concentrations. For example, when UV light and TiO_2_ were used to degrade PFOA at 40 kHz, the reaction mechanism was hypothesised to depend on pH, degrading via superoxide radicals (${{O_{2}}^{∙}}_{}^{-}$) at pH 4 and OH radicals at pH 10 [60]. Ultrasound was presumed to enhance TiO_2_ dispersion, by reducing its particle size (increasing total surface area) and enhancing mass transfer rate between the liquid bulk and the catalyst [60]. Permanganate in PFOA sonolysis was presumed to aid reaction by formation of MnO_2_ particles during sonication [47]. Ultrasound alone generated a PFOA rate constant of ≈1.5×10^−3^ min^−1^ and increases in permanganate concentration enhanced this logarithmically [47]. Enhanced PFOA degradation with sulfate ions from Na_2_SO_4_ at 40 kHz was attributed to the increased oxidising capability of sulfate radicals compared to sonolytically-produced ^•^OH radicals [64]. It was proposed that PFOA was degraded via both a sonochemical mechanism at the bubble surfaces and a chemical mechanism in the bulk liquid, since degradation rates followed the order r_sulfate_ < r_ultrasound_ < r_sulfate+ultrasound_ [64]. However, the rate of removal with just sulfate was close to zero. In similar work, PFOA defluorination was enhanced, up to a maximum persulfate concentration, after which the defluorination rate was reduced [59]. This was attributed to increased sulphate radical anion (${{\mathrm{SO}}_{4}}^{{}_{∙2-}}$) scavenging other radicals that could degrade PFOA. It was suggested this reaction also proceeds via both thermal and radical mechanisms, since both methanol (volatile) and benzoic acid (non-volatile) limited the reaction, via presumed quenching of collapse temperature and scavenging of ^•^OH and ${\mathrm{SO}}_{4}^{\cdot 2-}$ radicals, respecively [59], [161].

Sodium bicarbonate combined with sonolysis at 40 kHz and 150 W L^−1^, under argon, achieved much faster PFOA defluorination than previous low-frequency (20, 40 and 43 kHz) works, (97.2 % of 120 µM PFOA in four hours, with a pseudo-first order rate constant of 0.024 min^−1^) [58]. Conversely, bicarbonate ions were detrimental to PFAS destruction at 612 kHz, with up to 15 % reduction in reaction rate, depending on the concentration added [46]. At the lower frequency, PFOA defluorination was attributed to the production of carbonate radicals ($C{O_{3}}^{∙-}$) via the reaction of sonolytically produced hydroxyl radicals with bicarbonate ($\mathrm{HC}O_{3}^{-}$) [58]. However, OH radical concentration was not explicitly measured and the increased reaction rate may also be due to a decrease in pH due to nitric acid formation [55], which enhanced PFOA’s hydrophobicity (3.5.7, 3.5.9). Similar to persulfate [59], carbonate radical addition during sonication showed an optimum concentration (30 mM), beyond which reaction rates decreased [58]. However, no tests were done to show the rate of degradation by solely $CO_{3}^{∙-}$. The authors also concluded that PFOA is degraded in both the liquid (bulk solution) and vapour phase (bubble core) [58]. Finally, periodate and bromide have been used to degrade PFOA at 40 kHz and 500 W L^−1^ power. Formation of iodate radicals (IO^•^_3_^-^ almost completely mineralised 170 µM PFOA in 2 h, with a pseudo-first order rate constant of 0.022 min^−1^. Bromine ions were suggested to react with OH radicals to form dibromine radicals ${\mathrm{Br}}_{2}^{∙-})$ which to react with PFOA at the bubble interface, to enhance degradation, however the effect of bromide without periodate was not measured [62].

##### 3.5.6.2 Short chain formation

In sulfate and persulfate-enhanced sonolysis, shorter chain PFCAs are produced, as observed at dual ultrasonic frequencies (20 and 43 kHz) and evidenced by a low percentage fluoride balance [51]. Thus, we hypothesise that PFAS may initially undergo headgroup removal at the bubble surface, followed by radical attack (∙OH and ${\mathrm{SO}}_{4}^{∙2-}$) in or close to the bubble wall. This mechanism was also proposed for permanganate radical degradation of PFOA, since only ultrashort chain (C2-C3) PFCA by-products were detected [47], although the fluorine balance indicated that undetected longer intermediates were formed. Around 20 % of the initial PFAS was converted into shortened by products under degradation using permanganate [47] while around 7.7 % of initial PFOA was converted to shortened by-products when using UV light [60]. Both these values are much higher than those seen at higher frequencies [41], [62], [36], [37], which suggests a slower truncation mechanism/rate, possibly due to the reduced bubble oscillation rate and hence slower uptake of the low volatility sort chain by-products.

##### 3.5.6.3 Degradation of novel PFECs

**Fig. 5:**
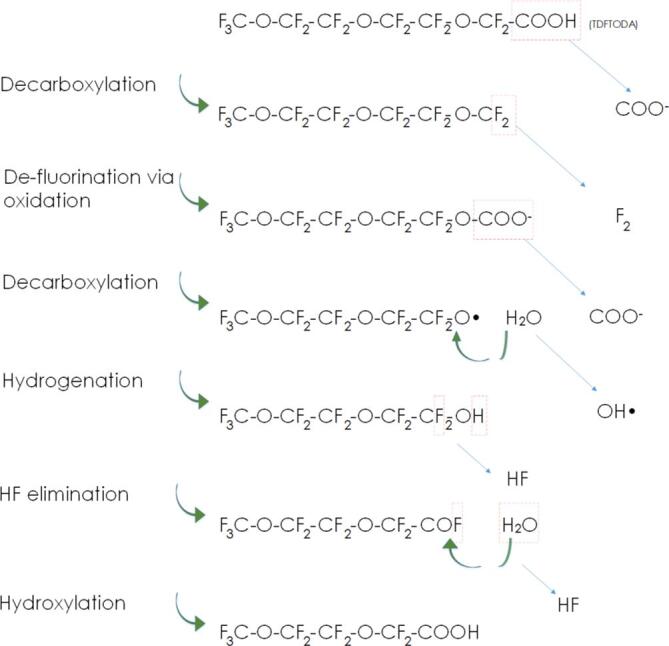
Truncation mechanism for PFECs with removal of C_2_F_4_O group. Plausible and observed reaction given to demonstrate possible repeated truncation from Tridecafluoro-3,6,9-trioxadecanoic acid (TDFTODA) to NFDOHpA.

Persulfate addition also enhanced the sonolysis of five perfluoroalkyl ether carboxylates (PFECs) but not two perfluoroalkyl ether sulfonates (PFESs) [61]. Shortened PFEC formation was observed and ascribed to repetitive truncation, as for PFCAs [37] but with removal of -C_2_F_4_O- groups instead of -CF_2_- groups [61]. The effect of persulfate on PFECs was assumed as the generation of ${\mathrm{SO}}_{4}^{∙2-}$ which encourages decarboxylation [61], similarly proposed for sonolysis of perfluoro(2-ethoxyethane) sulfonic acid (See Section 3.5.6.1) (PFEES) at 500 kHz [44]. The degradation mechanism suggested by the original authors differs significantly from those of PFYXs (Section 3.1) (Fig. 5).

##### 3.5.6.4 Summary of radical effects

Comparison of low frequency works suggests that radical additives may enhance the decarboxylation of PFOA, with different enhancement levels for the different radicals utilised. The size distribution of cavitating bubbles depend on the applied frequency, with lower frequencies generally having a greater variation in bubble size, greater average size, and being fewer in number [138]. This may explain why both thermal and radical degradation mechanisms are seen at these frequencies. More work is needed to prove the mechanistic effects of the radicals proposed by the authors, to compare relative effects of each additive at identical concentrations, and to estimate relative costs for treatments with and without oxidant addition.

#### 3.5.7 Effect of co-contaminants

PFAS pollution is found in soils, lakes and oceans and often stems from AFFFs, landfill leachate and other complex mixtures of organic and inorganic species [11], [48]. Studies on PFAS sonolysis in ground water considered co-organic [40] and inorganic [46] effects, in which both PFAS and co-contaminant concentrations were artificially adjusted (spiked) prior to sonication. Four works have investigated AFFF sonolysis [49], [50], one investigated simulated ground water by addition of cations [47] and another investigated IDW [52]. Recent work also considered low frequency sonolysis of PFAS in sewage sludge but without successful degradation [85]. These works are analysed here.

##### 3.5.7.1 Co-organics in landfill leachate

Compared to degradation of PFOS/PFOA in Milli-Q water (MQ), landfill groundwater degradation rates at 354 kHz were 61 % and 56 % lower for 0.20 µM PFOS and 0.24 µM PFOA, respectively, due to the presence of other organics (TOC 20 mg L^−1^) [40]). Significant effects on sonochemical efficiency were only noted above 10 mM organics concentration, more than 40,000x the concentration of the individual PFAS [40]. At this concentration, the decline in reaction rate was dramatic, however, the reaction was consistently first order [40], indicating an unchanged reaction mechanism. Rate altering effects were specific to the organics tested. Natural dissolved organic matter (DOM), mainly humic and fulvic acid, had no measurable impact on reaction rates, despite representing 75 % of the TOC. Meanwhile, volatile organic compounds (VOCs), comprised of methanol, acetone, ethyl acetate, isopropyl alcohol and methyl-t-butyl-ether, reduced reaction rates by between 50 and 100 %. Thus, it was theorised that volatile compounds competitively adsorbed at the bubble interface to block reaction sites, and/or quenched the bubble collapse temperature via evaporation into the bubble [40], as observed previously [162], [163]. Quenching effects were thought to be proportional to a species’ concentration, heat capacity, hydrophobicity, and heat of dissociation (total bond breakage energy). Hence, large organics had greater quenching effect per mol, due to their greater number of bonds and hence greater heat of dissociation [40]. The negative impact of organic species on PFAS reaction rates was negated with sonozone (O_3_ + ultrasound), which produces OH radicals to degrade competitive organic species, but not PFAS [40], further suggesting that high-frequency PFAS sonolysis is not via a radical means. Competing organics impacted PFOS degradation more than PFOA [40], likely due to PFOA’s lower required dissociation temperature, which mitigated quenching effects. Similarly, 10 mM *tert*-butyl alcohol (TBA), added to 10 mg L^−1^ PFOS, reduced the rate constant from 0.016 to 0.012 min^−1^, although this was attributed to radical scavenging rather than interfacial competition [37] (Section 3.1).

##### 3.5.7.2 Inorganics in landfill leachate

Landfill waste water containing inorganics reduced reaction rates of 0.20 µM PFOS and 0.24 µM PFOA under 612 kHz ultrasound by 20.5 % and 29.7 %, respectively, compared to MQ water [46]). The cations present (Ca^2^^+^, Na^+^, NH_4_^+^ and Mg^2^^+^) were assessed at 10 mM concentration and had little effect (≈±5 %) on degradation rates, compared to anions (added as sodium salts) at the same concentration due to their smaller size and hence lower water molecule gathering abilities [46]. Perchlorate increased the first order rate constant by up to 11 % and 47 % for PFOA and PFOS, respectively; nitrate and chloride had slightly positive effects on rates (1 – 4 % for PFOA, up to 18 % for PFOS); and bicarbonate and sulfate both reduced rates by around 10 – 25 %, accounting for the majority of negative effects seen in the landfill sample [46]. Therefore, sulfate formation from PFSA degradation might be self-limiting. In prior work, sodium chloride, potassium iodide, and carbon tetrachloride similarly enhanced the ultrasonic degradation rates of chlorobenzene [164], phenol [165], p-ethylphenol, and 2,4-dinitrophenol [166] by up to 200 %, suggesting that the large halide ions are beneficial in sonolysis due to attraction of several water molecules and enhanced salting out effects, consistent with the Hofmeister series effects on protein solubility [46]. pH 4 – 11 had little impact on PFOX degradation in landfill wastewater, but below pH 4 rates were enhanced by ≈100 % for PFOS and ≈30 % for PFOA. This is likely due to the high concentration of bicarbonate in the groundwater, which was neutralised during acidification or speciation of the PFAS from dissolved to molecular salt, increasing its attraction to the bubble interface [46] (see Section 3.5.9 for further pH effects discussion). Rate reduction effects were apparent at 1 mM inorganic concentrations and concentrations up to 10 mM made little difference, except in the case of NaHCO_3_ and NaClO_4_ [46]. Sodium bicarbonate similarly reduced the rate of ultrasonic 2,4-dinitrophenol degradation by 66 % in a previous study [166]. The various anion effects were also attributed to (de)stabilisation (salting out/in) of PFOX molecules. However, it is not clear why PFOS rates were more affected than PFOA. The enhancement of degradation rate for PFOS solutions with pH below 4 brought the rate close to that of PFOA at pH 8, while degradation of PFOA (at lower pH) was less affected, suggesting it’s closeness to a maximum intrinsic reaction rate.

Conversely, at low frequencies, cations reduced reaction rates more significantly. In one study, 1 mM cation addition reduced reaction rates in the order r_MQ_ (≈1.30 ×10^−2^ min^−1^) > r_Cu(II)_ (≈1.28 ×10^−2^ min^−1^) > r_Fe(II)_ (≈1.10 ×10^−2^ min^−1^) > r_Fe(III)_ (≈ 1.00 ×10^−2^ min^−1^) when added to PFOA and permanganate, sonicated at 40 kHz. This was attributed to complexation of 5.9 %, 5.7 % and 66.4 % of the starting 132 μM PFOA with Cu(II), Fe(II), and Fe(III) ions, respectively [47]. In a 20 kHz system utilising persulfate oxidation, Co^2^^+^ reduced PFOA degradation by ≈91 %, although the CoCl_2_ concentration added was not given [59]. The difference in response between cationic effects at low and high frequencies is likely due radical degradation mechanisms dominating in the low frequency system, as opposed to bubble dynamics and PFAS surface adsorption dominated mechanisms at mid-high frequencies, which are impacted by changes in ionic strength. One could combine the observations of these three works [59], [46], [47] in attempt to predict the behaviour of ClO_4_^−^, NO_3_^–^, Cl^−^, Na^+^, Ca^2+^, Mg^2+^, NH_4_^+^, HCO_3_^–^ and SO_4_^2–^ relative to Cu^2+^, Fe^2+^ and Fe^2+^ as well as Co^2+^. However, there are vast differences in applied ultrasound parameters, i.e. frequency (612 kHz vs 40 kHz), power density (250 W L^−1^ vs 180 W L^−1^), temperature (10 °C vs 30 °C), and initial pH (7.9 vs 4.0), so this is likely inaccurate.

##### 3.5.7.3 Surfactants

The addition of three different surfactant types modified the rate of PFOA degradation and defluorination rates under sonolysis at 40 kHz [65]. For PFOA and surfactant concentrations of 0.12 mM, relative rates followed the order; Cetrimonium bromide (CTAB, Cationic) ≫ TritonX-100 (Non-ionic) > No surfactant > (SDS, Anionic) [65]. The reduced degradation rate by SDS was thought to be due to competition with PFOA (also an anionic surfactant) at the bubble interface. The addition of other surfactants can, however, reduce resistance to bubble interfacial mass transfer (rectified diffusion) and, hence, hasten bubble growth rates [153]. Further, surfactants can homogenise sonoluminescent bubble distributions by reducing bubble coalescence, bubble size [167], and hence, the attenuation of the standing wave [168], which may enhance PFAS-bubble mass transfer rates. Cationic CTAB was thought to enhance rates by positively charging the bubble surface to enhance the attraction of PFOA anions and reduction of the critical micelle concentration (CMC) of PFOA. At high concentrations, CTAB’s effect on bubble surface charges was also thought to minimise bubble losses due to coalescence. However, fluoride release was low (6 – 13 %) and was theorised to be caused by the solutions’ high ionic strengths and competitive degradation of the co-surfactants [65].

##### 3.5.7.4 AFFFs

AFFFs typically contain water, several PFAS (of varying length and functional group), and other organics, such as butyl carbitol and glycol [48], [49], [50]. Despite their high concentrations, co-organics had little effect on PFAS degradation rates in AFFFs, presumed to be due to PFAS’ competitive surfactant nature at bubble surfaces [48], which is consistent with work on landfill leachate [40]. This is likely due to the high dilution factors used in the particular study (500 – 50,000x), which possibly also negated the viscosity enhancing effects of co-organics, which would reduce bubble collapse temperature. These dilution factors also reduced PFOS concentration from ≈ 7.3 mM in the initial foams [40], to 0.146–14.6 µM. When diluted below the kinetic transition concentration (Section 4.3), it is difficult to decouple the matrix effects from concentration effects, as seen elsewhere at 12,500x dilution [52]. In similar work in a large dual frequency reactor, co-organics such as glycol butyl ether and tolyl-triazole were suggested to be broken down by radical reactions close to bubble interfaces, while PFAS degraded at the interface, and more volatile species degraded in the bubble core [50]. During treatment, F^−^ release was 50 % less than anticipated from the measured PFAS loss, indicating formation of short chain PFAS not accounted for in post-sonolysis analysis or degassing of volatile fluorochemicals [48]. Conversely, SO_4_^2–^ yield was greater than anticipated [48], indicating interference from other sulfur containing species, not seen during tests in MQ water [39] or groundwater [40]. These conclusions were agreed in another AFFF sonolysis work [49]. Finally, competitive degradation between co-PFAS has been observed in AFFFs. For example, during sonication of a 5,000 x dilution FC-600 foam, PFOS rapidly reached pseudo-first order degradation kinetics, despite a significantly higher initial concentration than co-PFAS; PFOA, PFHS, PFHA and PFBS, which all remained in the zero-order regime throughout [48]. Hence, PFAS structural effects seen in pure solutions (Section 3.5.5) remain relevant in more complex systems.

#### 3.5.8 Solution temperature

**Fig. 6:**
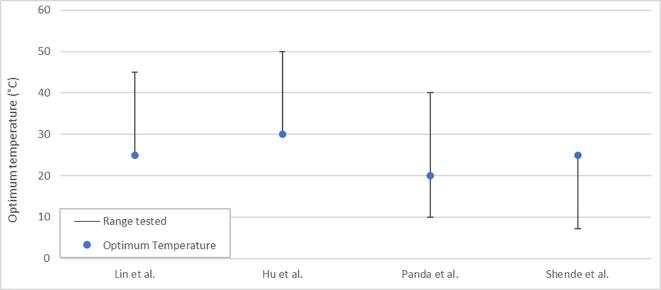
Range and optimum temperatures tested in PFAS sonolytic works (note that the data for Shende et al. indicates the range of final temperatures achieved during the reaction, not the temperature during treatment).

Solution temperature is a critical parameter in chemical kinetics but has been studied little in PFAS sonolysis, compared to other parameters. At 40 kHz, increasing solution temperature from 25 to 45 °C adversely effected the defluorination of PFOA, with or without oxidant addition [64], attributed to reduced surface tension, and subsequent reduced PFAS partitioning to the bubble interface [64]. Similar findings were noted in other low frequency works [47] (Fig. 6 and Table 12).

**Table 12:** Reaction parameters used in studies where temperature effects were assessed.

Reaction Parameters								Ref
PFAS	ν (kHz)	C_0,PFAS_ (μM)	PD (W L^−1^)	Degradation rate (min^−1^)	pH_0_	Oxidant	C_0, Oxidant_ (mM)	
PFOA	40	120	500	Not given	4.3	Na_2_SO_4_	0 & 46	Lin et al. [64]^
PFOA	40	132	180	1.5×10^−2^	4.0	KMnO_4_	6.0	Hu et al. [47]^
PFOA & KPFOS	20	2×10^−4^	3,750	7.5×10^−2^ 6.8×10^−2^	2.0	None	N/A	Panda et al. [63]
PFOA & PFOS	575	0.102 & 0.113	77	2.4×10^−2^ 7.0×10^−3^	Not given	None	N/A	Shende et al.[68]

Dissolved gas assumed to be air for all.

^Mechanical stirring also applied (RPM not detailed in works).

In similar work, an optimum of 20 °C in the range 10 – 40 °C was reported for PFOX sonolysis at 20 kHz, attributed to the formation of greater gas or vapour within the bubble cavity [63]. The higher temperatures tested were suggested to enhance PFAS diffusion to the bubbles (possibly through reduced viscosity [57]) but also to reduce collapse intensity [63]. Conversely, at 575 kHz, increasing solution temperature from 14.5 °C to 30 °C enhanced PFOX degradation [68]. These observations are consistent with the sonochemical mechanisms seen at low frequencies and works outside of PFAS sonolysis. For example, the rate of KI oxidation decreased with increasing temperature (from 40 to 75 °C) at 20 kHz (ultrasonic horn), with negative effects more pronounced at higher calorimetric powers (investigated range 210 – 720 W L^−1^), which shifted the optimum temperatures below 10°C [147]. KI oxidation followed similar trends at 900 kHz [147], suggesting high frequency (plate) sonication mechanisms are enhanced by increased bulk temperature, up to a maximum. This is in agreement with theoretical approximations of a simplified version of the Rayleigh-Plesset equation, which predicts increased bubble collapse temperatures from increased liquid bulk temperatures [146] (Eq. (19)). Further, these results show the connectivity of frequency, temperature, and power. In theory, increasing temperature will 1) increase vaporisation of the bulk liquid into the bubble core, reducing collapse temperatures [146] and 2) decrease gas solubility, reducing nucleation rates, but also 3) decrease the cavitation power threshold by reducing surface tension/viscosity. However, most PFAS are not considered volatile [18] so their vaporisation is not likely affected by bulk temperature. To further understand temperature effects, more work is needed in combination with high frequency, power and, various PFAS structures [146].

(19) $$T_{\mathrm{Max}}=\frac{T_{0}P_{a}\left(\gamma -1\right)}{P_{v}}$$

Where:

$T_{\mathrm{Max}}$ = Maximum collapse temperature

$T_{0}$ = Initial bulk liquid temperature

$P_{a}$ = Acoustic pressure

γ = Polytropic index

$P_{v}$ = Solution vapour pressure

#### 3.5.9 pH

**Fig. 7:**
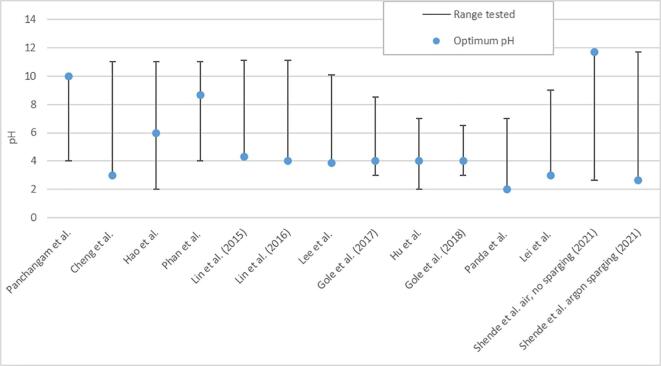
Optimum solution pH and pH ranges tested for all known PFAS sonolytic works.

Since many PFAS are acids, several sonolytic works have observed acidic initial pH values [36], [38], [43], [44], [47], [49], [50], [51], [53], [59], [60], [63], [64], [65], which become more acidic during sonolysis [36], [38], [44], [49], likely due to the formation of radicals and several acid species such as HF, carbonic acid from dissolved CO_2_ and nitrous and nitric acids, formed from air saturated systems [38]. Conversely, some low frequency (40 kHz) studies observed minor increases in pH during PFAS sonication [60], [64], although no explanation was given. As discussed in Section 3.5.7, cations do not have a significant effect on PFAS degradation rates in landfill leachate, except for hydrogen ions [46]. pH ≤ 4 can enhance reaction rates [53], [46], [47], [50], [51], [62], [63], [64], [65], although some studies showed optimum values at pH 6 [59], 8.65 [58] and 10 [60] (see Fig. 7 and Table 13). For several of these works, the “optimum” was found at the lowest pH value tested, thus the true optimum may be lower.

**Table 13:** Reaction parameters used in studies where pH effects were assessed.

Reaction Parameters								Reference
PFAS	C_0,PFAS_ (μM)	ν (kHz)	PD (W L^−1^)	Temperature (°C)	Dissolved gas	Oxidant/Reagents	C_0, Oxidant_ (mM)	
PFOA	120	40	166.7 + 5.3 UV	25	O_2_	RdH or Sol-gel TiO_2_	8.26	Panchangam et al.^ [60]
PFOA + PFOS	0.200 + 0.240	612	250	10	Argon	None	N/A	Cheng et al. [46]
NH_4_PFOA	46.4	20	6000	25	Air	K_2_S_2_O_8_	10	Hao et al. [59]
PFOA	120.75	40	150	25	Air*	Na_2_CO_3_	–	Phan et al.^ [58]
PFOA	120	40	500	25	Air*	Na_2_SO_4_	46	Lin et al. (2015)^ [64]
PFOA	120	40	500	25	Air*	CTAB	0.12	Lin et al. (2016)^ [65]
PFOA	170.1	40	500	25	Air*	KIO_4_	4.5	Lee et al. [62]
PFOS	2600	1 MHz + 500 kHz	131.9	25	Air*	None	N/A	Gole et al. (2017) [53]
PFOA	132	40	180	30	Air*	KMnO_4_	10	Hu et al. [47]
Various in AFFF	1.6	1 MHz + 500 kHz	131.9	–	Air*	None	N/A	Gole et al. (2018) [50]
PFOA + PFOS	2×10^−4^	20	3750	20	Air*	None	N/A	Panda et al. [63]
PFOA	10	20	550	–	Air*	Na_2_SO_4_	7.04	Lei et al. [51]
PFOA + PFOS	0.102 + 0.113	575	77	21.3	Air	None	N/A	Shende et al. (2021)[68]
PFOA + PFOS	0.102 + 0.113	575	77	21.3	Argon sparging	None	N/A	Shende et al. (2021)[68]

- Not discussed in text.

*Assumed as not discussed in text.

^Mechanical stirring also applied (RPM not detailed in works).

For low frequency works with oxidising agents, pH effects on radical formation and destruction [64] are specific to the radical-mediated mechanism. For example, sonolysis in a carbonate solution required an alkaline pH of 8.65 to be effective [58]; little effect of pH was seen with sulfate ions [64]; TiO_2_ and UV light degraded PFOA best at pH 10, followed by pH 4 then pH 7 [60], periodate coupled with sonolysis at pH 3.9 defluorinated PFOA approximately 10 % faster than at pH 10.1 [62] and permanganate coupled with sonolysis was most effective at pH 4, in the range 2 - 7 [47]. Further, a switch from reactions mediated predominanlty via sulfate radicals to hydroxyl radicals, impacted by pH, was reasoned to be behind an optimum of pH 6 for persulfate oxidation [59]. At 612 kHz, altering the pH from pH 8 to 11 had a slightly negative impact on PFOA reaction rate (-6 %) and very slightly positive (+1 %) effect on that of PFOS. However, reducing pH from 8 to pH 3.9 increased the reaction rate of both PFOA and PFOS by 4.4 % and 33.9 % respectively [46]. Similarly, in a large scale dual frequency reactor (500 kHz and 1 MHz), an initial pH of 4 produced a higher release of fluoride than pH 3 and 6.5, with very little fluoride released from PFOS at pH 8.5 [53]. Similar results (optimal pH 4) were reported for treatment of AFFFs in the same reactor, where F^−^ release rates were enhanced with decreasing pH but SO_4_^2–^ formation was negatively affected as pH was reduced from 4 to 3 [50]. Note that below pH 3.2, H^+^ and F^−^ ions speciate to HF, which may explain the reduced F^−^ formation. Preference for lower pH values, independent of oxidative mechanisms, was attributed to PFAS’ low pKa values, meaning that they exist as ionised compounds until low pH causes them to reform with H^+^ ions and adsorb into the bubble interface due to increased hydrophobicity [47], [63], [131]. This may result in alternate degradation mechanisms (such as pyrolysis/plasma reactions) than if the solute was in ionic form and degraded in the bulk solution (oxidative/radical-mediated reactions) [131], [156], [163], [169].

The pKa for PFOA has historically been reported as 3.8 [170], although others argue that it is actually closer to -0.5 [171], and for PFOS is thought to be -3.3 [172]. This may also explain why pH changes had little impact, except at lower values, in landfill leachate sonolysis [46]. Due to these low pka values, partitioning effects are not expected to be significant. Low pH is thought to positively charge the bubble wall [57], [173], reducing coalescence and increasing the attraction of hydrophobic PFAS to the bubble surface [53], [62], [64] (40 kHz – 1 MHz). While only a small number of PFAS have been investigated for pH effects on sonolytic reaction rate, one can expect similar optimal pH ranges for other PFAS chain lengths as it has been shown that the pKa values of carboxylic acids are relatively constant with varied chain length [169]. From a safety perspective, it should be considered whether the increases in rates observed compensate for the risk of forming of hydrofluoric acid at pH ≤ 3.2.

## 4 Comparison of PFAS remediation technologies

To date, at least 20 different processes have been researched for PFAS remediation, and their removal mechanisms are almost as numerous. Several experimental and commercial technologies exist to remove (but not destroy) PFAS from contaminated water and soil, as reviewed elsewhere [75], [76], [77], [78], [79]. These include; sorbents, such as granular activated carbon [78], [174], [175], [176], membrane treatments like reverse osmosis and nanofiltration [24], [75], [78], [177], [178], chemical removal processes such as ion exchange [24], [76], [78], [178], [179], [180], [181] and physical removal methods, thermal desorption for soils [75], [182] and ozofractionation for water [3], [28], [76], [183], [184]. Since these treatments cannot breakdown PFAS, they must be used in conjunction with some form of destructive technology [75]. Similarly, several destructive technologies are most effective at concentrations exceeding environmental levels or those found in diluted AFFFs [11], [46], [48], [50], [54], [57], [77], which necessitates a pre-destructive separation/concentration step [75], [78], [185]. Further, co-contaminants may affect degradation rates (Section 3.5.7) and the effluents from destructive technologies used to breakdown PFAS may require further treatment, prior to water emission to the environment. Hence, large scale remediation of PFAS will likely involve the use of a “treatment train”, as concluded previously [75], [78], [185]. This section will therefore compare the efficiency and practically of sonolysis with competing destructive technologies, not separative ones.

### 4.1 Biological degradation

While common for other aqueous contaminants, biological treatment of PFAS has yet to see significant results [75]. Despite providing a theoretically high density energy source to microbes [186], PFAS and other highly fluorinated compounds do not readily occur naturally, hence living species lack evolutionary need or opportunity to metabolise the compounds [187]. Further, the high C—F bond strength and shielding of C—C bonds present a significant barrier for microbial attack [75]. Complete bio-mineralisation of PFAS is not yet possible [75], however, biotransformation of some poly-fluorinated molecules into shorter perfluoroalkyl acids (PFAAs) via attack of the alpha carbon or non-fluorinated regions has been observed [29], [188], similar to the radical attack susceptibility of PFEECs (Section 3.5.5). Such bio-transformations take several weeks, with only partial degradation achieved [10] (e.g. 28 % fluoride release from PFOA over 157 days, with significant PFPeA formation [189]). The resultant PFAA’s are generally shorter and less bio-accumulative [190] but more stable in water and less easily removed or destroyed by other technologies [41], [178], [191].

### 4.2 Chemical degradation

While typically inert, PFAS show chemical activity in select scenarios. Advanced oxidative processes (AOPs) are commonly used to degrade organic pollutants, but struggle to degrade PFAS, particularly PFSAs [57], [97]. Similarly to low frequency ultrasound treatment (Section 3.5.1), several chemical reagents have been tested for PFAS treatment by AOPs [77]. Oxidation via heated persulfate, shows reactions rates of around 1.12 µM h^−1^, generation of short PFAAs and low effectivity for samples containing organics, sediments and PFSAs. The low pH requirements can generate HF gas and perchlorate, while the high temperatures required, compared to ultrasound (85 °C), limit process efficiency [192], [193]. Lower temperature (≤45 °C) degradation in a combined activated carbon-persulfate system reduced the activation energy of persulfate formation. However, defluorination was slow (<60 % after 12 h), even at relatively high concentrations, 120.6 µM [194]. Alternatively, high temperature iron-catalysed reduction in sub-critical water shows high reaction rates (≈60 µM h^−1^) but at the cost of even higher temperatures (≈350 °C) [195]. The process also has limited effectiveness for short chains and low F^−^ release, suggesting several fluorinated by-products [195].

Electrochemical oxidation (EO) processes achieve enhanced hydroxyl radical (OH) generation, using high voltage electrodes with chemical additives [113], [196]. EO using Na_2_SO_4_ as an electrolyte degraded ≈7 µM h^−1^ PFOX and resisted co-organic interference more than chemical oxidation and AOPs [113]. Recently, the use of boron doped diamond electrodes has augmented this process and has seen electrochemical treatment become an efficient treatment for PFAS [196], [197], [198], [199]. However, the problem of high organic fluoride by-products remains, with 50 % of fluorine not released [196]. EO may completely mineralise PFAS, but only after a sequential defluorination step which may favour short chain formation [57], which is yet to be proven. The OH radicals generated only exist for fractions of a second, which restricts reactions to an area close to the electrode surface and thus may be ineffective for large volumes. Photochemical oxidation, uses UV light to augment oxidation of PFAS [114], [200], [201], [202]. Reaction times vary from several days to several hours, requiring 2x - 30x more energy per PFAS molecule degraded than ultrasound [36] (Table 14) and the process generates significant short chain PFAAs [114], [200], [201], [202].

**Table 14:** Comparison of G-values for various PFOS degradation works and conditions[36].

Technology	Treatment time (h)	Initial Concentration (mg L ^−^^1^)	Efficiency (g kW ^−^^1^ h ^−^^1^)	Short chain formation	Ref
Electrochemical	4	0.0152	0.00033	Up to 50 %	[196]
Photochemical	240	20.0	0.00133	Observed, significant quantity indicated (71 % F ^−^ release)	^218^
Photochemical, ferric ion	60	10.0	0.00290	∼14 % of initial mass	[219]
Electrochemical	2	8.00	0.00566	Not discussed	[198]
Sonication, 618 kHz	3	5.00	0.00801	Almost none implied (∼100 % F ^−^ release)	[39]
Photochemical, persulfate	2	10.0	0.00900	Observed, significant quantity indicated (76 % F ^−^ release)	[220]
Photochemical, propanol	24	20.0	0.01520	Not discussed	[218]
Sonication, 400 kHz	4	9.42	0.01550	1 % of initial mass	[36]
Plasma	4	50.0	0.02600	Not discussed, none implied	[221]
Sonication, 400 kHz	2	9.42	0.02610	13 % of initial mass	[36]
Sonication, 358 kHz	3	59.5	0.04170	Not discussed	[45]
Plasma	0.5	0.0001	0.06900	Observed, 5.65 % of initial mass after 40 min)	[115]
Plasma	1	100	0.62100	Observed, significant quantity indicated (∼30 % F ^−^ release	[205]

An alternative physiochemical treatment is that of aqueous plasma degradation. Similar to EO, plasma treatment generates aqueous O· and ·OH radicals and transient electrons, using high voltages [203]. Plasma treatments shows fast reaction rates (36 µM h^−1^ PFOA) and high energy efficiency (Table 14) compared to sonolysis, even in the presence of co-contaminants [115]. Further, reactions are not limited by electrode surface area, as for EO. However, the high levels of short chain PFAS, small acid molecules and related compounds make plasma less efficient for complete mineralisation. In example works, 97.5 %+ of the fluorine remained as organics in the aqueous phase, with the rest forming in gaseous by-products [115], [203]. Increasing the plasma pulse energy is predicted to reduce formation of short chains [203], which might make ultrasound a more competitive treatment. PFAS plasma degradation has been investigated in several works and has been trialled with volumes up to 4 L [204], [205], [206], [207], suggesting potential for scale up.

### 4.3 Physical/Thermal degradation

Historically, PFAS removed by separation technologies were incinerated, since it theoretically guarantees complete destruction [97]. However, this is not always achieved practically and there is a lack of flue-gas analysis and energy efficiency considerations in related works [208]. In one example, 100 kg h^−1^ of wood pellets was used to treat 300 g h^−1^ of PTFE, here combined gaseous and solid phase fluorine emissions represented 56 – 78 % of fluorine in the initial PTFE, while the remaining 22 – 44% of fluorine was not identified [209]. Elsewhere, PFAS incineration at 1,000 °C led to a 21.6 – 45.1% conversion of fluorine to toxic HF [210]. Unreacted PFAS can also be emitted to the atmosphere given insufficient incinerator temperatures or residence times [208], [211], although, similar work completed by PFAS manufacturers, 3M, showed only C1-C2 fluorinated by-products in the effluent [212]. The fluorinated by-products of incineration typically require even higher temperatures to be degraded than the starting species [57]. Use of cement kilns for CFC, HFC and PFAS incineration has shown advancements in recent years [213]. However, incineration is undesirable for the majority of PFAS pollution, which is aqueous, dilute and disperse. Since incineration is typically carried out at fixed locations [57], this necessitates pre-concentration at the affected site and transportation to the incinerator. A novel and highly experimental physical treatment for solid PFAS is that of ball milling, whereby solid material is churned inside a rotating drum containing several hardened solid spheres, which break down the material via attrition [214]. One group has shown up to 100 % degradation of pure PFOX powders using ball milling, with reportedly zero toxic by-products [215], [216], [217]. However, characterisation of energy consumption and testing of AFFF/environmental samples is yet to be reported.

### 4.4 Comparison with sonolysis

As reviewed, sonolysis combines both physical and chemical degradation methods and is well studied for several different parametric effects. Reaction rates can also be much more rapid than other technologies (up to 61.3 µM h^−1^) and, when compared on the basis of G-value (Eq 20a) [36] (Table 14), sonolysis was shown to be the second most energy efficient technology, behind plasma treatment.

If considered instead in terms of grams of fluoride released per kWh, the gap between plasma’s efficiency and that of ultrasound is significantly reduced [36], [115], [203], [205]. Further, the values presented may not account for all devices used in the experimental methodology (for example heaters, chillers, pumps etc.), just the main instrument of the experiment (ultrasonic amplifier [36], plasma generator [115], [203], [205], electrochemical cell [114], [200], [201], [202] etc.). Sonolysis is also not chain length selective, like some technologies (such as electrochemical oxidation [114], [200], [201], [202], incineration [212], plasma [115], [203], etc.), since removal occurs sequentially from long to short chains and from PFCAs to PFSAs [41], [43]. To this end, we conclude that sonolysis is currently the most pragmatic technology for large scale PFAS treatment, although plasma treatment is a fierce competitor. [222]

(20a) $$\mathrm{SE}=\frac{\left(C_{0}-C_{t}\right)N_{0}V}{\mathrm{tP}}$$

Where:

$\mathrm{SE}=Number\;of\;PFAS\;molecules\;degraded\;per\;unit\;energy\;\left(molecules\;{\mathrm{kJ}}^{-1}\right)$

$C_{t}$ = $\mathrm{Concentration}\;\mathrm{of}\;\mathrm{reactant}\;\mathrm{at}\;\mathrm{time}\;t\;(mol\;L^{-1})$

$C_{0}$ = $\mathrm{Initial}\;\mathrm{concentration}\;\mathrm{of}\;\mathrm{reactant}\;\mathrm{at}\;\mathrm{time}\;t=0\;(mol\;L^{-1})$

$N_{0}$ = $\mathrm{Avogadros}\;\mathrm{Number}\;(6.023\times 10^{23}\;molecules\;mol^{-1})$

V = Liquidvolume(L)

t = Timeoverwhichreactionwascompleted(s)

P = Power(W)  

For comparison between works, where reaction orders may be different due to initial PFAS concentrations (and reaction rates) which vary by orders of magnitude, some authors also propose using energy consumed per order of magnitude concentration reduction (Eq 20a) [52]. Concentration and mass equivalents of Eq 20a and Eq 20b are also used commonly. [52]

(20b) $${\mathrm{SE}}_{O}=\frac{log\left(C_{0}/C_{t}\right)}{\mathrm{tP}}$$

Where:

${\mathrm{SE}}_{O}=Orders\;of\;magnitude\;concentration\;change\;per\;unit\;energy\;\left({\mathrm{kJ}}^{-1}\right)$

## 5 Summary and further hypotheses

### 5.1 Degradation routes

Due to the complexity and speed of sonochemical reactions, PFAS sonolysis mechanisms are convoluted. However, at mid-high frequencies, the initial steps are generally agreed. These are 1) orientation of PFAS at bubble interfaces, with the hydrophobic perfluoro tail entering the gaseous bulk and the hydrophilic headgroup preferring the liquid bulk, followed by 2) headgroup cleavage, upon bubble collapse. Evidence of these initiating steps include bubble saturation kinetics observed over increasing concentrations as well as SO_4_^2–^ and short chain PFCA/PFSA production. The driving force for headgroup cleavage is disputed and may be; high internal/interfacial bubble temperatures, solvated electron release, radical formation, or a combination of the three. The cleaved perfluorinated tail may then degrade within one collapse event, with shortened by-products formed from PFAS fragment recombination in the liquid bulk, or repeated cleavage of the CF_2_-COOH group and subsequent oxidation of the exposed perfluoro tail over several bubble lifetimes. Given strong evidence for both and the different by-products formed under differing conditions, we hypothesise that both routes co-exist and the extent of either depends on applied conditions.

Low frequency reaction mechanisms are more varied and less well understood, due to the numerous oxidants investigated to enhance degradation. However, it is generally agreed that interfacial bubble adsorption and radical addition/generation are significant parameters at low frequencies. The slower reaction rates at low frequencies, despite higher individual bubble collapse temperatures, suggests the sonolysis mechanism is indeed chemically, not thermally, dominated. However, cavity oscillations and collapse events, which attract and degrade PFAS, are reduced at low frequency, making mechanistic comparison between frequencies difficult. Slower bubble oscillations leading to reduced uptake of short chain PFAS may also explain the higher by-product concentration at low frequencies. Further, short chains may be more radical resistant due to a shorter and stuffer C-F backbone.

### 5.2 Rate limiting steps and kinetics

Due to the reaction steps proposed in Section 5.1, PFAS destruction is limited by; 1) The rate of PFAS adsorption by the bubble, 2) available bubble surface area, and 3) the rate of headgroup cleavage. The degree of mineralisation and product release is also limited by diffusion of the sonochemical intermediate or short chain products (both aqueous and gaseous) into the bubble. Modelling of the reaction kinetics is thus far based on the diffusion rate of PFAS from the bulk liquid to the bubble interface, and on bubble surface availability. However, such modelling does not consider factors such as, frequency, power, or PFAS structure and hence does not yet predict sonochemical degradation rates. Further, long chain PFAS reaction rates were not found to be diffusion limited, unlike those of short chains. At low PFAS/bubble concentrations, PFAS may not always be within oscillating distance of the bubbles, hence the passive diffusion toward the bubble may dominate, even for long chains. Until the mechanisms of PFAS sonolysis are agreed upon, accurate kinetic models are unlikely to be developed.

### 5.3 Reaction products, product measurement, and stoichiometric equations

Reported PFAS sonolysis products are predominantly four inorganic species: CO, CO_2_, F^−^ and SO_4_^2–^. Shortened chain PFAS form, both in the bulk aqueous and bulk gaseous phases, which at high and low frequencies typically represent <1 % and up to 20 %, respectively, of the initial PFAS. Analysis of reaction products gives insight into the mechanisms and extent of PFAS treatment in contaminated matrices, however, no metric in isolation can indicate reaction completeness, due to multiple possible fluorinated by-products. Chromatographic techniques for measuring PFAS concentrations, should avoid sample contamination from the device and account for all/significant breakdown products. Complete stoichiometric equations for PFOA and PFOS sonolysis are presented in Section 3.2, which assumed CO was an intermediate, oxidised to CO_2_ and that the PFAS counterion is H^+^ (more commonly a metal cation). The effect of other counterions on the reaction equation was not assessed.

### 5.4 Parametric effects

We demonstrated here the need to understand matrix effects in simpler systems, prior to more complex ones. A *meta*-analysis between studies led to the conclusion that the kinetic transition concentration between zero and first/pseudo first order is near constant at 15 – 40 µM. Optimal PFOX degradation conditions were found to be 300 – 500 kHz frequency, 20 – 30 °C temperature, and pH 3.2 – 4.0. Optimal values for AFFF dilution factor, radical additives, additive concentration, and power/power density could not be derived due to a lack of comparable research. Concentration is likely the most critical factor in PFAS treatment efficiency since it determines the rate order. Following this, frequency controls the bubble count, size distribution, reaction surface area, oscillation rate, collapse temperature and radical products. Frequency effects are also specific to the PFAS structure, hence PFAS structural effects are of comparable importance. Reaction rates change linearly with power until sufficiently high/low-power levels, where a plateau and rapid decrease occur due to decoupling (high power) or insufficient power to form cavitation (low power). However, power studies are lacking for novel PFAS, AFFFs, and ground waters as well as under dual frequency sonication. This is significant, since modern PFAS usage shows preference for replacement of C8+ compounds with shorter, novel, less environmentally persistent and less bioaccumulative alternatives [75], not the typical PFOA and PFOS researched in many papers discussed here.

pH, dissolved gas choice, co-contaminants, and temperature have a less significant effect on the reaction rate, typically varying well within an order of magnitude. Formation of PFAS-metal ion complexes has implications for the treatment of contaminated groundwaters since this may obscure both accurate measurement and complete removal of PFAS. Low frequency sonication (20 – 100 kHz) is largely controlled by the concentration and type of oxidative additives used, however, no work has yet utilised rate enhancement by addition of anions at mid-high frequencies (100 – 1,000 kHz). The findings on the aforementioned parametric effects are summarised in Table 15.

**Table 15:** Summary of parametric findings in this work.

Estimated significance for sonolysis rate	Parameter	Key findings	Value for optimum PFOX sonolysis
1	PFAS concentration/AFFF dilution factor	Kinetic transition concentration for PFOX is consistently 15 – 40 µM Optimum AFFF dilution factor depends on AFFF brand, rate measurement technique and reaction conditions	>15 μM
2	Applied ultrasonic frequency	Lower frequencies less effective for PFAS treatment without oxidising additives Higher frequencies (600 kHz+) are effective for short chain PFAS due to rapid bubble motion	300 – 500 kHz
3	Power density/intensity	Shows linear effect on reaction rates, within certain limits. At low/high values, reaction rates plateau/reduce	Not found
4	PFAS structure	Affects partitioning to the bubble interface and hence degradation rates Long chains (≥C6), low degree of fluorination and PFCAs degraded more easily than short chains, perfluorinated substances and PFSAs	N/A
5	Oxidising additives	Affects the type and concentration of radicals formed and hence sonolysis rate (not yet tested under high-frequency use)	Na_2_S_2_O_8_
6	pH	Low pH speciates PFAS to molecular (not ionic) state which encourages bubble interfacial partitioning Low pH treats bicarbonate in polluted environmental samples	≈4
7	Dissolved gas	Gases with a high polytropic index enhanced collapse temperature and hence sonolysis rates	Argon
8	Co-contaminants: Inorganics Organics	Follow Hofmeister effects of salting in (negative) and salting out (positive) PFAS to bubble interface Quench bubble collapse temperature and competitively degrade at bubble interface	ClO_4_^−^ > Cl^−^ ≈ NO_3_^–^ None
9	Solution temperature	Mildly affects PFAS interfacial partitioning rate and collapse temperature	20 – 30 °C

**Fig. 8:**
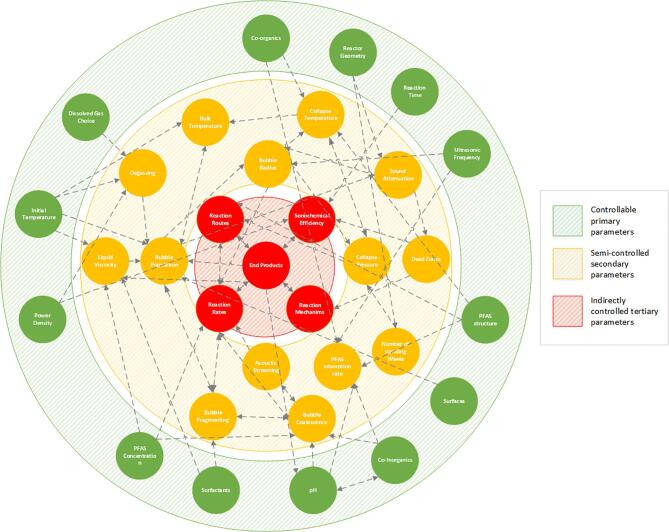
Levels of control and interconnected nature of sonolysis reaction parameters.

Overall, observation of rate/mechanistic effects for any one parameter is difficult, due to the high number of interdependent ultrasonic parameters and the levels of control one may have over any in each experiment (the complexity of which is summarised graphically in Fig. 8). Note that the complexity represented in this figure is not assumed to be definitive and may also apply to other sonochemical reactions, not just PFAS sonolysis. With continued research, it is hoped that the relative importance of each connection in the figure can be clarified and eventually replaced with numerical descriptors.

### 5.5 PFAS destructive technologies and use of a treatment train

As concluded in Section 4.4, high frequency sonolysis poses the greatest potential for remediation of all PFAS destructive technologies, which, unlike other technologies summarised in Table 14, does not generate significant short chains, toxic by-products, or have bias for longer chains. This comes at the cost of increased power usage yet, accounting for complete mineralisation and improvements in understanding parametric effects, sonolysis has high potential. PFAS sonolysis is also comparatively well studied, with more than 30 research works to date on the subject, considering not only the reactor conditions but also the medium to be treated and solutions to problems of viscosity (AFFFs) and high pH (landfill leachate). Development of a treatment train incorporating sonolysis with a concentrating step and post-sonolysis treatment remains the ultimate ambition of this research field. Such technologies will be of increasing importance, as governments around the world move to impose stricter limits on PFAS emissions [223]. For the eventual industrial scale treatment, future work must consider the cost/benefit analysis of any parametric effects as well as the design and operation of reactors for efficient sound transmission (avoiding attenuation) through large liquid volumes.

## 6 Conclusions

30+ works on PFAS sonolysis and a multitude of works which either expand on sonolytic reaction theory or the nature of PFAS chemistry, pollution and their treatment are compared here. At mid-high ultrasonic frequencies (100 – 1,000 kHz), PFAS can be degraded quickly (mg h^−1^) without the addition of oxidative agents, while low frequencies (20 – 100 kHz) typically struggle. The reaction initiating and limiting mechanisms are shown to be PFAS interfacial adsorption at the bubble interface, followed by cleavage between the headgroup and perfluoro-tail. Arguments against this are discredited using experimental observations and consideration of surfactant chemistry. Further, explanations are offered for the differences in observed reaction products of PFOA and PFOS, which leads to the novel derivation of complete stoichiometric equations for both compounds. These equations were proposed based on observations of previous by-products and a select few assumptions regarding reaction completeness, which considers CO as an intermediate. Further, plausible and observed breakdown products are derived for 18 common PFCAs and PFSAs, as well as and 9 more novel perfluoro-ether carboxylates (PFECs). The *meta*-analysis revealed a near-universal concentration at which PFOX sonolysis rate switches between zero and first/pseudo first order (15 – 40 μM) due to the saturation at the site of reaction (the bubble interfacial surface). An optimum ultrasonic frequency range (300 – 500 kHz), pH (≈4), PFAS concentration (≥15 – 40 μM) and temperature (20 – 30 °C) for fast PFOX degradation were derived from experimental data. However, for other PFAS structures these values differ (short chains degraded faster at 600+ kHz). While not yet tested on higher frequencies, the optimum oxidising agent for low frequency PFAS sonolysis appeared to be sodium persulfate (Na_2_S_2_O_8_). Optimal values for AFFF dilution factor, radical concentration and power density remain to be derived but could be in the region of 10 - 900x, 1.0 g L^−1^ and 350 W L^−1^, respectively, based on work reviewed. Areas still lacking in understanding include the latter half of the PFAS sonolytic degradation mechanism at high frequency, reaction rate modelling, the relative effects of some co-contaminant species and parametric effects on novel PFAS compounds, such as perfluorinated- sulphonamides and ether carboxylates. Sonolysis research is advanced when compared with other destructive PFAS treatment technologies, with research not only into effective reaction conditions but also the treatment of several matrices (pure solutions, landfill leachates, firefighting foams and investigation derived waste). Further, sonolysis offers complete mineralisation, while other treatments generate significant fluorinated by-products. There is, however, a trade off in the cost of electricity consumed for complete mineralisation. Sonolysis also offers potential as a large-scale remediation process, however with pending areas of research to ensure large scale efficiency.

## Declaration of Competing Interest

The authors declare the following financial interests/personal relationships which may be considered as potential competing interests: Madeleine Bussemaker reports financial support was provided by Arcadis.

## References

[1] Rayne S., Forest K. (2009). Perfluoroalkyl sulfonic and carboxylic acids: A critical review of physicochemical properties, levels and patterns in waters and wastewaters, and treatment methods. J Environ Sci Heal – Part A Toxic/Hazardous Subst Environ Eng..

[2] Martin J.W., Kannan K., Berger U. (2004). Peer Reviewed: Analytical Challenges Hamper Perfluoroalkyl Research. Environ Sci Technol..

[3] KEMI (Swedish Chemicals Agency). Occurrence and use of highly fluorinated substances and alternatives. Report from a government assignment. 2015:112. https://www.kemi.se/global/rapporter/2015/report-7-15-occurrence-and-use-of-highly-fluorinated-substances-and-alternatives.pdf.

[4] Smith J.W.N., Beuthe B., Dunk M. (2016). Environmental fate and effects of polyand perfluoroalkyl substances (PFAS). CONCAWE Reports..

[5] Houtz E.F., Higgins C.P., Field J.A., Sedlak D.L. (2013). Persistence of perfluoroalkyl acid precursors in AFFF-impacted groundwater and soil. Environ Sci Technol..

[6] Oliaei F., Kriens D., Weber R., Watson A. (2013). PFOS and PFC releases and associated pollution from a PFC production plant in Minnesota (USA). Environ Sci Pollut Res..

[7] Gallen C., Drage D., Kaserzon S. (2016). Occurrence and distribution of brominated flame retardants and perfluoroalkyl substances in Australian landfill leachate and biosolids. J Hazard Mater..

[8] Bell C, Gentile M, Kalve E, et al. *Emerging Contaminants Handbook*. (Boca Raton FL, ed.). Boca Ranton: Taylor abd Francis; 2019.

[9] Harding-Marjanovic K.C., Houtz E.F., Yi S., Field J.A., Sedlak D.L., Alvarez-Cohen L. (2015). Aerobic Biotransformation of Fluorotelomer Thioether Amido Sulfonate (Lodyne) in AFFF-Amended Microcosms. Environ Sci Technol..

[10] Tseng N., Wang N., Szostek B., Mahendra S. (2014). Biotransformation of 6:2 Fluorotelomer alcohol (6:2 FTOH) by a wood-rotting fungus. Environ Sci Technol..

[11] Ahrens L. (2011). Polyfluoroalkyl compounds in the aquatic environment: A review of their occurrence and fate. J Environ Monit..

[12] Jahnke A., Berger U., Ebinghaus R., Temme C. (2007). Latitudinal gradient of airborne polyfluorinated alkyl substances in the marine atmosphere between Germany and South Africa (53° N-33° S). Environ Sci Technol..

[13] Liu C., Gin K.Y.H., Chang V.W.C., Goh B.P.L., Reinhard M. (2011). Novel perspectives on the bioaccumulation of PFCs – The concentration dependency. Environ Sci Technol..

[14] Gorrochategui E., Pérez-Albaladejo E., Casas J., Lacorte S., Porte C. (2014). Perfluorinated chemicals: Differential toxicity, inhibition of aromatase activity and alteration of cellular lipids in human placental cells. Toxicol Appl Pharmacol..

[15] L. Wilder, R. Worley and Breysse P. Community Exposures to Per- and Polyfluoroalkyl Substances in Drinking Water: A National Issue. J Environ Health. 2016;80(2):38-41. https://www.questia.com/library/journal/1G1-504340179/community-exposures-to-per-and-polyfluoroalkyl-substances.

[16] Stockholm Convention. Press Release – COP4 – Geneva, 8 May 2009. Stockholm: Stockholm Convention; 2009. http://chm.pops.int/Implementation/PublicAwareness/PressReleases/COP4Geneva,9May2009/tabid/542/Default.aspx.

[17] Stockholm Convention. Call for information and follow-up to the decisions adopted by the Conference of the Parties to the Stockholm Convention at its ninth meeting (Geneva, Switzerland from 29 April to 10 May 2019). 2019;(1):1-15. http://chm.pops.int/TheConvention/ConferenceoftheParties/Meetings/COP9/FollowuptoCOP9/tabid/8043/Default.aspx.

[18] Buck R.C., Franklin J., Berger U. (2011). Perfluoroalkyl and polyfluoroalkyl substances in the environment: Terminology, classification, and origins. Integr Environ Assess Manag..

[19] OECD. ENVIRONMENT DIRECTORATE JOINT MEETING OF THE CHEMICALS COMMITTEE AND THE WORKING PARTY ON CHEMICALS, PESTICIDES AND BIOTECHNOLOGY: TOWARD A NEW COMPREHENSIVE GLOBAL DATABASE OF PER- AND POLYFLUOROALKYL SUBSTANCES (PFASs): SUMMARY REPORT ON UPDATING THE OE. *Ser Risk Manag*. 2018;No. 39.(39):1-24. http://www.oecd.org/officialdocuments/publicdisplaydocumentpdf/?cote=ENV-JM-MONO(2018)7&doclanguage=en.

[20] 3M. *The Science of Organic Fluorochemistry*.; 1999. http://www.fluoridealert.org/wp-content/pesticides/pfos.fr.final.docket.0006.pdf.

[21] O’Hagan D. (2008). Understanding organofluorine chemistry. An introduction to the C-F bond. Chem Soc Rev..

[22] US EPA. PFAS Master List of PFAS Substances. USEPA Comptox Chemicals List. https://comptox.epa.gov/dashboard/chemical_lists/pfasmaster. Published 2020.

[23] Air Force Civil Engineer Center 2261 Hughes Avenue, Suite 155 Lackland AFB T 78236-9853. FINAL PRELIMINARY ASSESSMENT REPORT FOR PERFLUORINATED COMPOUNDS AT VOLK FIELD COMBAT READINESS TRAINING CENTER CAMP DOUGLAS, WISCONSIN.; 2015. https://cswab.org/wp-content/uploads/2018/05/Volk-Field-Camp-Douglas-PFAS-Preliminary-Assessment-June-2015.pdf.

[24] Rahman M.F., Peldszus S., Anderson W.B. (2014). Behaviour and fate of perfluoroalkyl and polyfluoroalkyl substances (PFASs) in drinking water treatment: A review. Water Res..

[25] Trier X., Granby K., Christensen J.H. (2011). Polyfluorinated surfactants (PFS) in paper and board coatings for food packaging. Environ Sci Pollut Res..

[26] IPEN Expert Panel (2019).

[27] Zushi Y., Yamamoto A., Tsunemi K., Masunaga S. (2017). Revaluation of stockpile amount of PFOS-containing aqueous film-forming foam in Japan: gaps and pitfalls in the stockpile survey. Environ Sci Pollut Res..

[28] Thompson J., Eaglesham G., Reungoat J. (2011). Removal of PFOS, PFOA and other perfluoroalkyl acids at water reclamation plants in South East Queensland Australia. Chemosphere..

[29] Liu J., Mejia Avendaño S. (2013). Microbial degradation of polyfluoroalkyl chemicals in the environment: A review. Environ Int..

[30] Chen H., Zhang C., Han J., Yu Y., Zhang P. (2012). PFOS and PFOA in influents, effluents, and biosolids of Chinese wastewater treatment plants and effluent-receiving marine environments. Environ Pollut..

[31] Sunderland E.M., Hu X.C., Dassuncao C., Tokranov A.K., Wagner C.C., Allen J.G. (2019). A review of the pathways of human exposure to poly- and perfluoroalkyl substances (PFASs) and present understanding of health effects. J Expo Sci Environ Epidemiol..

[32] Kempisty DM, Racz L. Forever Chemicals: Environmental, Economic, and Social Equity Concerns with PFAS in the Environment. 1st ed. Boca Raton: CRC Press; 2021. 10.1201/9781003024521.

[33] Nam S.N., Han S.K., Kang J.W., Choi H. (2003). Kinetics and mechanisms of the sonolytic destruction of non-volatile organic compounds: Investigation of the sonochemical reaction zone using several OH · monitoring techniques. Ultrason Sonochem..

[34] Hoffmann MRA of ultrasonic irradiation for the degradation of chemical contaminants in water, Hua I, Höchemer R. Application of ultrasonic irradiation for the degradation of chemical contaminants in water. Ultrason Sonochem. 1996;3(3). 10.1016/S1350-4177(96)00022-3.

[35] Lu Y., Weavers L.K. (2002). Sonochemical desorption and destruction of 4-chlorobiphenyl from synthetic sediments. Environ Sci Technol..

[36] Wood R.J., Sidnell T., Ross I. (August 2019). Ultrasonic degradation of perfluorooctane sulfonic acid (PFOS) correlated with sonochemical and sonoluminescence characterisation. Ultrason Sonochem..

[37] Moriwaki H., Takagi Y., Tanaka M., Tsuruho K., Okitsu K., Maeda Y. (2005). Sonochemical decomposition of perfluorooctane sulfonate and perfluorooctanoic acid. Environ Sci Technol..

[38] Shende T., Andaluri G., Suri R.P.S. (2019). Kinetic model for sonolytic degradation of non-volatile surfactants: Perfluoroalkyl substances. Ultrason Sonochem..

[39] Vecitis C.D., Park H., Cheng J., Mader B.T., Hoffmann M.R. (2008). Kinetics and Mechanism of the Sonolytic Conversion of the Aqueous Perfluorinated Surfactants, Perfiuorooctanoate (PFOA), and Perfluorooctane Sulfonate (PFOS) into Inorganic Products. J Phys Chem A..

[40] Cheng J., Vecitis C.D., Park H., Mader B.T., Hoffmann M.R. (2008). Sonochemical degradation of perfluorooctane sulfonate (PFOS) and perfluorooctanoate (PFOA) in landfill groundwater: Environmental matrix effects. Environ Sci Technol..

[41] Campbell T.Y., Vecitis C.D., Mader B.T., Hoffmann M.R. (2009). Perfluorinated surfactant chain-length effects on sonochemical kinetics. J Phys Chem A..

[42] Rodriguez-Freire L., Balachandran R., Sierra-Alvarez R., Keswani M. (2015). Effect of sound frequency and initial concentration on the sonochemical degradation of perfluorooctane sulfonate (PFOS). J Hazard Mater..

[43] Campbell T., Hoffmann M.R. (2015). Sonochemical degradation of perfluorinated surfactants: Power and multiple frequency effects. Sep Purif Technol..

[44] Fernandez N.A., Rodriguez-Freire L., Keswani M., Sierra-Alvarez R. (2016). Effect of chemical structure on the sonochemical degradation of perfluoroalkyl and polyfluoroalkyl substances (PFASs). Environ Sci Water Res Technol..

[45] Vecitis C.D., Park H., Cheng J., Mader B.T., Hoffmann M.R. (2008). Enhancement of perfluorooctanoate and perfluorooctanesulfonate activity at acoustic cavitation bubble interfaces. J Phys Chem C..

[46] Cheng Jie, Vecitis ChadD., Park Hyunwoong, Mader MRH Brian T., Cheng JIE, Vecitis CD, (2010). Sonochemical Degradation of Perfluorooctane Sulfonate (PFOS) and Perfluorooctanoate (PFOA) in Groundwater: Kinetic Effects of Matrix Inorganics. Environ Sci Technol..

[47] Hu Y bo, Lo SL, Li YF, Lee YC, Chen MJ, Lin JC. Autocatalytic degradation of perfluorooctanoic acid in a permanganate-ultrasonic system. Water Res. 2018;140:148-157. 10.1016/j.watres.2018.04.044. 10.1016/j.watres.2018.04.044 29704759

[48] Vecitis C.D., Wang Y.J., Cheng J., Park H., Mader B.T., Hoffmann M.R. (2010). Sonochemical Degradation of Perfluorooctanesulfonate in Aqueous Film-Forming Foams. Environ Sci Technol..

[49] Rodriguez-Freire L., Abad-Fernández N., Sierra-Alvarez R. (2016). Sonochemical degradation of perfluorinated chemicals in aqueous film-forming foams. J Hazard Mater..

[50] Gole V.L., Sierra-Alvarez R., Peng H., Giesy J.P., Deymier P., Keswani M. (2018). Sono-chemical treatment of per- and poly-fluoroalkyl compounds in aqueous film-forming foams by use of a large-scale multi-transducer dual-frequency based acoustic reactor. Ultrason Sonochem..

[51] Lei Y., Tian Y., Sobhani Z., Naidu R., Fang C. (2020). Synergistic degradation of PFAS in water and soil by dual-frequency ultrasonic activated persulfate. Chem Eng J..

[52] Singh Kalra S., Cranmer B., Dooley G. (2021). Sonolytic destruction of Per- and polyfluoroalkyl substances in groundwater, aqueous Film-Forming Foams, and investigation derived waste. Chem Eng J..

[53] Gole V.L., Fishgold A., Sierra-Alvarez R., Deymier P., Keswani M. (November 2017). Treatment of perfluorooctane sulfonic acid (PFOS) using a large-scale sonochemical reactor. Sep Purif Technol..

[54] Cao H., Zhang W., Wang C., Liang Y. (2020). Sonochemical degradation of poly- and perfluoroalkyl substances – A review. Ultrason Sonochem..

[55] Pflieger R, Nikitenko SI, Cairós C, Mettin R. Characterization of Cavitation Bubbles and Sonoluminescence. (Springer, ed.). Springer; 2019. 10.1007/978-3-030-11717-7.

[56] Mason T.J., Lorimer J.P. (April 2002). Applied Sonochemistry. Appl Sonochemistry..

[57] Wood R.J., Lee J., Bussemaker M.J. (2017). A parametric review of sonochemistry: Control and augmentation of sonochemical activity in aqueous solutions. Ultrason Sonochem..

[58] Phan Thi L.-A., Do H.-T., Lo S.-L. (2014). Enhancing decomposition rate of perfluorooctanoic acid by carbonate radical assisted sonochemical treatment. Ultrason Sonochem..

[59] Hao F., Guo W., Wang A., Leng Y., Li H. (2014). Intensification of sonochemical degradation of ammonium perfluorooctanoate by persulfate oxidant. Ultrason Sonochem..

[60] Panchangam S.C., Lin A.Y.C., Tsai J.H., Lin C.F. (2009). Sonication-assisted photocatalytic decomposition of perfluorooctanoic acid. Chemosphere..

[61] Hori H., Nagano Y., Murayama M., Koike K., Kutsuna S. (2012). Efficient decomposition of perfluoroether carboxylic acids in water with a combination of persulfate oxidant and ultrasonic irradiation. J Fluor Chem..

[62] Lee Y.C., Chen M.J., Huang C.P., Kuo J., Lo S.L. (2016). Efficient sonochemical degradation of perfluorooctanoic acid using periodate. Ultrason Sonochem..

[63] Panda D., Sethu V., Manickam S. (2019). Kinetics and mechanism of low-frequency ultrasound driven elimination of trace level aqueous perfluorooctanesulfonic acid and perfluorooctanoic acid. Chem Eng Process – Process Intensif..

[64] Lin J.C., Lo S.L., Hu C.Y., Lee Y.C., Kuo J. (2015). Enhanced sonochemical degradation of perfluorooctanoic acid by sulfate ions. Ultrason Sonochem..

[65] Lin J.C., Hu C.Y., Lo S.L. (2016). Effect of surfactants on the degradation of perfluorooctanoic acid (PFOA) by ultrasonic (US) treatment. Ultrason Sonochem..

[66] Sekiguchi K., Kudo T., Sankoda K. (2017). Combined sonochemical and short-wavelength UV degradation of hydrophobic perfluorinated compounds. Ultrason Sonochem..

[67] Henglein A. (1985;40b(1):100–107.). Sonolysis of Carbon Dioxide, Nitrous Oxide and Methane in Aqueous Solution. Zeitschrift für Naturforsch B..

[68] Shende T., Andaluri G., Suri R., Gangadhar A., Suri R. (2021). Frequency-dependent sonochemical degradation of perfluoroalkyl substances. Sep Purif Technol..

[69] Phan Thi L.A., Do H.T., Lo S.L. (2014). Enhancing decomposition rate of perfluorooctanoic acid by carbonate radical assisted sonochemical treatment. Ultrason Sonochem..

[70] Campbell T., Hoffmann M.R. (2015). Sonochemical degradation of perfluorinated surfactants: Power and multiple frequency effects. Sep Purif Technol..

[71] Lee P.J., Bernier E.T., Fujimoto G.T., Shia J., Young M.S., Di Gioia A.J. (2008). Application Note: Acquity UPLC System Solution for Quantifying Trace Levels of Perfluorinated Compounds with an Acquity PFC. Analysis Kit..

[72] Mulabagal V., Liu L., Qi J., Wilson C., Hayworth J.S. (2018). A rapid UHPLC-MS/MS method for simultaneous quantitation of 23 perfluoroalkyl substances (PFAS) in estuarine water. Talanta..

[73] Horst J., McDonough J., Ross I., Houtz E. (2020). Understanding and Managing the Potential By-Products of PFAS Destruction. Groundw Monit Remediat..

[74] Gogate P.R. (2002). Cavitation: An auxiliary technique in wastewater treatment schemes. Adv Environ Res..

[75] Ross McDonough J., Miles J. (2018). A review of emerging technologies for remediation of PFASs. Remediation..

[76] Horst J., McDonough J., Ross I. (2018). Water Treatment Technologies for PFAS: The Next Generation. Groundw Monit Remediat..

[77] Arias Espana V.A., Mallavarapu M., Naidu R. (2015). Treatment technologies for aqueous perfluorooctanesulfonate (PFOS) and perfluorooctanoate (PFOA): A critical review with an emphasis on field testing. Environ Technol Innov..

[78] Merino N., Qu Y., Deeb R.A., Hawley E.L., Hoffmann M.R., Mahendra S. (2016). Degradation and Removal Methods for Perfluoroalkyl and Polyfluoroalkyl Substances in Water. Environ Eng Sci..

[79] Banks D., Jun B., Heo J., Her N., Min C. (August 2019). Selected advanced water treatment technologies for perfluoroalkyl and polyfluoroalkyl substances : A review. Sep Purif Technol..

[80] Pétrier C., Francony A. (1997). Ultrasonic waste-water treatment: Incidence of ultrasonic frequency on the rate of phenol and carbon tetrachloride degradation. Ultrason Sonochem..

[81] Torres-Palma RA, Serna-Galvis EA. Sonolysis. In: *Advanced Oxidation Processes for Wastewater Treatment: Emerging Green Chemical Technology*. Elsevier Inc.; 2018:177-213. 10.1016/B978-0-12-810499-6.00007-3.

[82] Torres R.A., Pétrier C., Combet E., Carrier M., Pulgarin C. (2008). Ultrasonic cavitation applied to the treatment of bisphenol A. Effect of sonochemical parameters and analysis of BPA by-products. Ultrason Sonochem..

[83] Wayment D.G., Casadonte D.J. (2002). Frequency effect on the sonochemical remediation of alachlor. Ultrason Sonochem..

[84] Kang J.W., Hung H.M., Lin A., Hoffmann M.R. (1999). Sonolytic destruction of methyl tert-butyl ether by ultrasonic irradiation: The role of O3, H2O2, frequency, and power density. Environ Sci Technol..

[85] Zhang W., Zhang Q., Liang Y. (2022). Ineffectiveness of ultrasound at low frequency for treating per- and polyfluoroalkyl substances in sewage sludge. Chemosphere..

[86] Chen W., Zhang X., Mamadiev M., Wang Z. (2017). Sorption of perfluorooctane sulfonate and perfluorooctanoate on polyacrylonitrile fiber-derived activated carbon fibers: In comparison with activated carbon. RSC Adv..

[87] Brusseau M.L. (2019). The influence of molecular structure on the adsorption of PFAS to fluid-fluid interfaces: Using QSPR to predict interfacial adsorption coefficients. Water Res..

[88] Yasui K., Tuziuti T., Lee J., Kozuka T., Towata A., Iida Y. (2008). The range of ambient radius for an active bubble in sonoluminescence and sonochemical reactions. J Chem Phys..

[89] Dehane A., Merouani S., Hamdaoui O., Alghyamah A. (2021). A comprehensive numerical analysis of heat and mass transfer phenomenons during cavitation sono-process. Ultrason Sonochem..

[90] Kim M., Li L.Y., Grace J.R., Yue C. (May 2009). Selecting reliable physicochemical properties of perfluoroalkyl and polyfluoroalkyl substances (PFASs) based on molecular descriptors. Environ Pollut..

[91] Love A.H., Hanna M.L., Reynolds J.G. (2005). Engineering surface functional groups on silica aerogel for enhanced cleanup of organics from produced water. Sep Sci Technol..

[92] Kotronarou A., Mills G., Hoffmann M.R. (1991). Ultrasonic irradiation of p-nitrophenol in aqueous solution. J Phys Chem..

[93] Sostaric J.Z., Riesz P. (2001). Sonochemistry of surfactants in aqueous solutions: An EPR spin-trapping study. J Am Chem Soc..

[94] Shende T., Andaluri G., Suri R. (2021). Power density modulated ultrasonic degradation of perfluoroalkyl substances with and without sparging Argon. Ultrason Sonochem..

[95] Hadad C.M., Rablen P.R., Wiberg K.B. (1998). C-O and C-S bonds: Stability, bond dissociation energies, and resonance stabilization. J Org Chem..

[96] Franklin J.L., Lumpkin H.E. (1952). Some C-S, H-S and S-S Bond Strengths by the Electron Impact Method. J Am Chem Soc..

[97] Khan M.Y., So S., da Silva G. (2020). Decomposition kinetics of perfluorinated sulfonic acids. Chemosphere..

[98] Vecitis C.D., Park H., Cheng J., Mader B.T., Hoffmann M.R. (2009). Treatment technologies for aqueous perfluorooctanesulfonate (PFOS) and perfluorooctanoate (PFOA). Front Environ Sci Eng China..

[99] Okitsu K., Suzuki T., Takenaka N., Bandow H., Nishimura R., Maeda Y. (2006). Acoustic multibubble cavitation in water: A new aspect of the effect of a rare gas atmosphere on bubble temperature and its relevance to sonochemistry. J Phys Chem B..

[100] Ciawi E., Rae J., Ashokkumar M., Grieser F. (2006). Determination of temperatures within acoustically generated bubbles in aqueous solutions at different ultrasound frequencies. J Phys Chem B..

[101] Yasui K., Tuziuti T., Kozuka T., Towata A., Iida Y. (2007). Relationship between the bubble temperature and main oxidant created inside an air bubble under ultrasound. J Chem Phys..

[102] Lundquist N.A., Sweetman M.J., Scroggie K.R. (2019). Polymer Supported Carbon for Safe and Effective Remediation of PFOA- and PFOS-Contaminated Water. ACS Sustain Chem Eng..

[103] Shih K., Wang F. (2013). Adsorption Behavior of Perfluorochemicals (PFCs) on Boehmite: Influence of Solution Chemistry. Procedia Environ Sci..

[104] Alegria A.E., Lion Y., Kondo T., Riesz P. (1989). Sonolysis of aqueous surfactant solutions. Probing the interfacial region of cavitation bubbles by spin trapping. J Phys Chem..

[105] McNamara W.B., Didenko Y.T., Suslick K.S. (1999). Sonoluminescence temperatures during multi-bubble cavitation. Nature..

[106] Hoffmann M.R., Hua I., Höchemer R. (1996). Application of ultrasonic irradiation for the degradation of chemical contaminants in water. Ultrason Sonochem..

[107] Cravotto G., Cintas P. (2006). Power ultrasound in organic synthesis: Moving cavitational chemistry from academia to innovative and large-scale applications. Chem Soc Rev..

[108] Mišík V., Riesz P. (1997). Effect of Cd2+ on the H atom yield in the sonolysis of water. Evidence against the formation of hydrated electrons. J Phys Chem A..

[109] Gutierrez M., Henglein A., Dohrmann J.K. (2005). Hydrogen atom reactions in the sonolysis of aqueous solutions. J Phys Chem..

[110] Nikitenko S.I., Di Pasquale T., Chave T., Pflieger R., Pfliege R. (2020). Hypothesis about electron quantum tunneling during sonochemical splitting of water molecule. Ultrason Sonochem..

[111] Sharipov G.L., Gareev B.M., Abdrakhmanov A.M. (2020). Confirmation of hydrated electrons formation during the moving single-bubble sonolysis: Activation of Tb3+ ion sonoluminescence by eaq- acceptors in an aqueous solution. J Photochem Photobiol A Chem..

[112] Dharmarathne L., Ashokkumar M., Grieser F. (2013). On the generation of the hydrated electron during the sonolysis of aqueous solutions. J Phys Chem A..

[113] Liang S, Pierce R “David,” Lin H, Chiang SYD, Huang Q “Jack.” Electrochemical oxidation of PFOA and PFOS in concentrated waste streams. Remediation. 2018;28(2):127-134. 10.1002/rem.21554.

[114] Hori H., Yamamoto A., Hayakawa E. (2005). Efficient decomposition of environmentally persistent perfluorocarboxylic acids by use of persulfate as a photochemical oxidant. Environ Sci Technol..

[115] Stratton G.R., Dai F., Bellona C.L., Holsen T.M., Dickenson E.R.V., Mededovic Thagard S. (2017). Plasma-Based Water Treatment: Efficient Transformation of Perfluoroalkyl Substances in Prepared Solutions and Contaminated Groundwater. Environ Sci Technol..

[116] Bentel M.J., Yu Y., Xu L. (2019). Defluorination of Per- and Polyfluoroalkyl Substances (PFASs) with Hydrated Electrons: Structural Dependence and Implications to PFAS Remediation and Management. Environ Sci Technol..

[117] Siefermann K.R., Liu Y., Lugovoy E. (2010). Binding energies, lifetimes and implications of bulk and interface solvated electrons in water. Nat Chem..

[118] Margulis N.A., Mal’tsev. (1968). The generation of hydrated electrons in an ultrasonic field. RussJPhysChem..

[119] Bussemaker M.J., Xu F., Zhang D. (2013). Manipulation of ultrasonic effects on lignocellulose by varying the frequency, particle size, loading and stirring. Bioresour Technol..

[120] Hart E.J., Fischer C.H., Henglein A. (1990). Sonolysis of hydrocarbons in aqueous solution. Int J Radiat Appl Instrumentation Part..

[121] Leong T., Collis J., Manasseh R. (2011). The role of surfactant headgroup, chain length, and cavitation microstreaming on the growth of bubbles by rectified diffusion. J Phys Chem C..

[122] O’Shea K.E., Aguila A., Vinodgopal K., Kamat P.V. (1998). Reaction pathways and kinetic parameters of sonolytically induced oxidation of dimethyl methyl-phosphonate in air saturated aqueous solutions. Res Chem Intermed..

[123] Fyrillas M.M., Szeri A.J. (1996). Surfactant dynamics and rectified diffusion of microbubbles. J Fluid Mech..

[124] Wu H., Zhou C., Pu Z., Yu H., Li D. (2019). Effect of low-frequency ultrasonic field at different power on the dynamics of a single bubble near a rigid wall. Ultrason Sonochem..

[125] Beckett M.A., Hua I. (2001). Impact of ultrasonic frequency on aqueous sonoluminescence and sonochemistry. J Phys Chem A..

[126] Storey BD, Szeri AJ. Water vapour , sonoluminescence and sonochemistry. 1999;456:1685-1709. 10.1098/rspa.2000.0582 Downloaded.

[127] Crum LA. Rectified diffusion. *Acoust cavitation Ser five*. 1984;(September):215-223.

[128] Hofmeister F. (1888). Zur Lehre von der Wirkung der Salze. Arch für Exp Pathol und Pharmakologie..

[129] Yang Z. (2009). Hofmeister effects: an explanation for the impact of ionic liquids on biocatalysis. J Biotechnol..

[130] Storey B.D., Szeri A.J. (2011). A reduced model of cavitation physics for use in sonochemistry. Proc R Soc A Math Phys Eng Sci..

[131] Price G.J., Ashokkumar M., Grieser F. (2004). Sonoluminescence Quenching of Organic Compounds in Aqueous Solution: Frequency Effects and Implications for Sonochemistry. J Am Chem Soc..

[132] Son Y., Lim M., Khim J. (2009). Investigation of acoustic cavitation energy in a large-scale sonoreactor. Ultrason Sonochem..

[133] Sunartio D., Ashokkumar M., Grieser F. (2007). Study of the coalescence of acoustic bubbles as a function of frequency, power, and water-soluble additives. J Am Chem Soc..

[134] Tronson R., Ashokkumar M., Grieser F. (2003). Multibubble sonoluminescence from aqueous solutions containing mixtures of surface active solutes. J Phys Chem B..

[135] Nanzai B., Okitsu K., Takenaka N., Bandow H. (2009). Sonochemical degradation of alkylbenzene sulfonates and kinetics analysis with a langmuir type mechanism. J Phys Chem C..

[136] Chiha M., Merouani S., Hamdaoui O., Baup S., Gondrexon N., Pétrier C. (2010). Modeling of ultrasonic degradation of non-volatile organic compounds by Langmuir-type kinetics. Ultrason Sonochem..

[137] Kanthale P., Ashokkumar M., Grieser F. (2008). Sonoluminescence, sonochemistry (H2O2 yield) and bubble dynamics: Frequency and power effects. Ultrason Sonochem..

[138] Brotchie A., Grieser F., Ashokkumar M. (2009). Effect of power and frequency on bubble-size distributions in acoustic cavitation. Phys Rev Lett..

[139] Asakura Y., Nishida T., Matsuoka T., Koda S. (2008). Effects of ultrasonic frequency and liquid height on sonochemical efficiency of large-scale sonochemical reactors. Ultrason Sonochem..

[140] Gogate P.R., Sutkar V.S., Pandit A.B. (2011). Sonochemical reactors: Important design and scale up considerations with a special emphasis on heterogeneous systems. Chem Eng J..

[141] Hua I., Höchemer H.H., Hoffmann M.R. (1995). Sonochemical Degradation of p-Nitrophenol in a ParaHel-Plate Near-Field Acoustical Processor. Environ Sci Technol..

[142] Prabhu A.V., Gogate P.R., Pandit A.B. (2004). Optimization of multiple-frequency sonochemical reactors. Chem Eng Sci..

[143] Zhou M., Yusof N.S.M., Ashokkumar M. (2013). Correlation between sonochemistry and sonoluminescence at various frequencies. RSC Adv..

[144] Mason T.J. (2000). Large scale sonochemical processing: Aspiration and actuality. Ultrason Sonochem..

[145] Matula T.J. (1999). Inertial cavitation and single-bubble sonoluminescence. Philos Trans R Soc A Math Phys Eng Sci..

[146] Adewuyi Y.G. (2001). Sonochemistry: Environmental Science and Engineering Applications. Ind Eng Chem Res..

[147] Entezari M.H., Kruus P. (1996). Effect of frequency on sonochemical reactions II. Temperature and intensity effects. Ultrason Sonochem..

[148] Van Iersel M.M., Benes N.E., Keurentjes J.T.F. (2008). Importance of acoustic shielding in sonochemistry. Ultrason Sonochem..

[149] Hatanaka S.I., Yasui K., Tuziuti T., Kozuka T., Mitome H. (2001;40(5 B):3856–3860.). Quenching mechanism of multibubble sonoluminescence at excessive sound pressure. Japanese J Appl Physics, Part 1 Regul Pap Short Notes Rev Pap..

[150] Gutiérrez M., Henglein A. (1990). Chemical action of pulsed ultrasound: Observation of an unprecedented intensity effect. J Phys Chem..

[151] Henglein A., Gutierrez M. (1990). Chemical effects of continuous and pulsed ultrasound: A comparative study of polymer degradation and iodide oxidation. J Phys Chem..

[152] Crum L.A., Gallemore J.B., Nordling D.A. (1972). Effect of Surface Tension on the Cavitation Threshold of Water. J Acoust Soc Am..

[153] Lee J., Kentish S., Ashokkumar M. (2005). Effect of surfactants on the rate of growth of an air bubble by rectified diffusion. J Phys Chem B..

[154] Petrier C., Jeunet A., Luche J.L., Reverdy G. (1992). Unexpected Frequency Effects on the Rate of Oxidative Processes Induced by Ultrasound. J Am Chem Soc..

[155] Brotchie A., Statham T., Zhou M., Dharmarathne L., Grieser F., Ashokkumar M. (2010). Acoustic bubble sizes, coalescence, and sonochemical activity in aqueous electrolyte solutions saturated with different gases. Langmuir..

[156] Ashokkumar M., Hall R., Mulvaney P., Grieser F. (2002). Sonoluminescence from Aqueous Alcohol and Surfactant Solutions. J Phys Chem B..

[157] Gogate P.R., Wilhelm A.M., Pandit A.B. (2003). Some aspects of the design of sonochemical reactors. Ultrason Sonochem..

[158] Ben Salem I., Guillermic R.M., Sample C., Leroy V., Saint-Jalmes A., Dollet B. (2013). Propagation of ultrasound in aqueous foams: Bubble size dependence and resonance effects. Soft Matter..

[159] Ouerhani T., Pflieger R., Ben Messaoud W., Nikitenko S.I. (2015). Spectroscopy of Sonoluminescence and Sonochemistry in Water Saturated with N2-Ar Mixtures. J Phys Chem B..

[160] Manin A.N., Shmukler L.E., Safonova L.P., Perlovich G.L. (2010). Partial molar volumes of some drug and pro-drug substances in 1-octanol at T = 298.15 K. J Chem Thermodyn..

[161] Singla R., Ashokkumar M., Grieser F. (2004). The mechanism of the sonochemical degradation of benzoic acid in aqueous solutions. Res Chem Intermed..

[162] Ashokkumar M., Vinodgopal K., Grieser F. (2000). Sonoluminescence quenching in aqueous solutions containing weak organic acids and bases and its relevance to sonochemistry. J Phys Chem B..

[163] Price G.J., Ashokkumar M., Cowan T.D., Grieser F. (2002). Sonoluminescence quenching by organic acids in aqueous solution: pH and frequency effects. Chem Commun..

[164] Seymour J.D., Gupta R.B. (1997). Oxidation of Aqueous Pollutants Using Ultrasound: Salt-Induced Enhancement. Ind Eng Chem Res..

[165] Mahamuni N.N., Pandit A.B. (2006). Effect of additives on ultrasonic degradation of phenol. Ultrason Sonochem..

[166] Guo Z., Feng R., Li J., Zheng Z., Zheng Y. (2008). Degradation of 2,4-dinitrophenol by combining sonolysis and different additives. J Hazard Mater..

[167] Lee J., Ashokkumar M., Kentish S., Grieser F. (2005). Determination of the size distribution of sonoluminescence bubbles in a pulsed acoustic field. J Am Chem Soc..

[168] Lee J., Vakarelski I.U., Yasiui K. (2010). Variations in the spatial distribution of sonoluminescing bubbles in the presence of an ionic surfactant and electrolyte. J Phys Chem B..

[169] Ashokkumar M., Mulvaney P., Grieser F. (1999). The effect of pH on multibubble sonoluminescence from aqueous solutions containing simple organic weak acids and bases. J Am Chem Soc..

[170] Goss K.U., Arp H.P.H. (2009). Comment on “Experimental pKa determination for perfluorooctanoic acid (PFOA) and the potential impact of pKa concentration dependence on laboratory-measured partitioning phenomena and envrionmental modeling”. Environ Sci Technol..

[171] Goss K.U. (2008). The pKa values of PFOA and other highly fluorinated carboxylic acids. Environ Sci Technol..

[172] Brooke D, Footitt A, Nwaogu TA. Environmental Risk Evaluation Report : 4-Tert-Octylphenol.; 2004.

[173] Takahashi M. (2005). ζ Potential of microbubbles in aqueous solutions: Electrical properties of the gas – Water interface. J Phys Chem B..

[174] Hansen M.C., Børresen M.H., Schlabach M., Cornelissen G. (2010). Sorption of perfluorinated compounds from contaminated water to activated carbon. J Soils Sediments..

[175] Calgon Carbon. *EMERGING CONTAMINANTS: PFOS AND PFOA*. https://www.calgoncarbon.com/app/uploads/ccc_pfoa_pfos_pfc_specsheet2018.pdf.

[176] Regenesis. *BREAKTHROUGH TREATMENT FOR PFAS CASE STUDY :* https://regenesis.com/en/treatment-of-pfas/.

[177] Steinle-Darling E., Reinhard M. (2008). Nanofiltration for trace organic contaminant removal: Structure, solution, and membrane fouling effects on the rejection of perfluorochemicals. Environ Sci Technol..

[178] Dickenson E., Higgins C. (2016). Treatment Mitigation Strategies for Poly-. and Perfluorinated Chemicals..

[179] Rusinek CA, Michigan. PFAS Remediation at MSU-Fraunhofer: Electrochemical Destruction Using Boron-Doped Diamond Electrodes. Urbana; 2019.

[180] Woodard S., Berry J., Newman B. (2017). Ion exchange resin for PFAS removal and pilot test comparison to GAC. Remediation..

[181] Dudley L.-A. (2012). Removal of Perfluorinated Compounds by Powdered Activated Carbon, Superfine Powdered Activated Carbon. and Anion Exchange Resins..

[182] Norris G., Al-Dhahir Z., Birnstingl J., Plant S.J., Cui S., Mayell P. (1999). A case study of the management and remediation of soil contaminated with polychlorinated biphenyls. Eng Geol..

[183] Dickson MD. United States Patent Application Publication Pub . No . : US 2018/0353542 A1. 2018;1.

[184] Dickson MD. United States Patent Application Publication Pub . No .: US 2014/0332121. 2014.

[185] Ross I., Hurst J., Burdick J., Kalve E., Houtz E., Pancras T. (2016). Remediation of Poly- and Perfluoro Alkyl Substances: New Remediation Technologies for Emerging. Challenges..

[186] Parsons J.R., Sáez M., Dolfing J., De Voogt P. (2002). Biodegradation of perfluorinated compounds. Food Chem Toxicol..

[187] Harper DB, O’Hagan D, Murphy CD. Fluorinated Natural Products: Occurrence and Biosynthesis. In: Gribble G, ed. *Natural Production of Organohalogen Compounds*. Berlin, Heidelberg, Heidelberg: Springer Berlin Heidelberg; 2003:141-169. 10.1007/b10454.

[188] Key B.D., Howell R.D., Criddle C.S. (1998). Defluorination of organofluorine sulfur compounds by Pseudomonas sp. strain D2. Environ Sci Technol..

[189] Luo Q., Lu J., Zhang H. (2015). Laccase-Catalyzed Degradation of Perfluorooctanoic Acid. Environ Sci Technol Lett..

[190] Conder J.M., Hoke R.A., De Wolf W., Russell M.H., Buck R.C. (2008). Are PFCAs bioaccumulative? A critical review and comparison with regulatory criteria and persistent lipophilic compounds. Environ Sci Technol..

[191] Eschauzier C., Beerendonk E., Scholte-Veenendaal P., De Voogt P. (2012). Impact of treatment processes on the removal of perfluoroalkyl acids from the drinking water production chain. Environ Sci Technol..

[192] Lee Y.C., Lo S.L., Kuo J., Lin Y.L. (2012). Persulfate oxidation of perfluorooctanoic acid under the temperatures of 20–40°C. Chem Eng J..

[193] Bruton T.A., Sedlak D.L. (2017). Treatment of Aqueous Film-Forming Foam by Heat-Activated Persulfate under Conditions Representative of in Situ Chemical Oxidation. Environ Sci Technol..

[194] Lee Y.C., Lo S.L., Kuo J., Huang C.P. (2013). Promoted degradation of perfluorooctanic acid by persulfate when adding activated carbon. J Hazard Mater..

[195] Hori H., Nagaoka Y., Yamamoto A. (2006). Efficient decomposition of environmentally persistent perfluorooctanesulfonate and related fluorochemicals using zerovalent iron in subcritical water. Environ Sci Technol..

[196] Uwayezu J.N., Carabante I., Lejon T. (2021). Electrochemical degradation of per- and poly-fluoroalkyl substances using boron-doped diamond electrodes. J Environ Manage..

[197] Liao Z., Farrell J. (2009). Electrochemical oxidation of perfluorobutane sulfonate using boron-doped diamond film electrodes. J Appl Electrochem..

[198] Sukeesan S., Boontanon N., Boontanon S.K. (2021). Improved electrical driving current of electrochemical treatment of Per- and Polyfluoroalkyl Substances (PFAS) in water using Boron-Doped Diamond anode. Environ Technol Innov..

[199] Nienhauser A.B., Ersan M.S., Lin Z., Perreault F., Westerhoff P., Garcia-Segura S. (2022). Boron-Doped Diamond Electrodes Degrade Short- and Long-Chain Per- and Polyfluorinated Alkyl Substances in Real Industrial Wastewaters. J Environ Chem Eng..

[200] Hori H., Hayakawa E., Einaga H. (2004). Decomposition of environmentally persistent perfluorooctanoic acid in water by photochemical approaches. Environ Sci Technol..

[201] Wang Y., Zhang P., Pan G., Chen H. (2008). Ferric ion mediated photochemical decomposition of perfluorooctanoic acid (PFOA) by 254 nm UV light. J Hazard Mater..

[202] Song Z., Tang H., Wang N., Zhu L. (2013). Reductive defluorination of perfluorooctanoic acid by hydrated electrons in a sulfite-mediated UV photochemical system. J Hazard Mater..

[203] Singh RK, Fernando S, Fakouri Baygi S, Multari N, Mededovic Thagard S, Holsen TM. Breakdown products from perfluorinated alkyl substances (PFAS) degradation in a plasma-based water treatment process. Environ Sci Technol. 2019;53:acs.est.8b07031. 10.1021/acs.est.8b07031. 10.1021/acs.est.8b07031 30768259

[204] Singh R.K., Brown E., Mededovic Thagard S., Holsen T.M. (November 2020). Treatment of PFAS-containing landfill leachate using an enhanced contact plasma reactor. J Hazard Mater..

[205] Lewis A.J., Joyce T., Hadaya M. (2020). Rapid degradation of PFAS in aqueous solutions by reverse vortex flow gliding arc plasma. Environ Sci Water Res Technol..

[206] Singh R.K., Multari N., Nau-Hix C. (2019). Rapid Removal of Poly- and Perfluorinated Compounds from Investigation-Derived Waste (IDW) in a Pilot-Scale Plasma Reactor. Environ Sci Technol..

[207] Singh R.K., Multari N., Nau-Hix C. (2020). Removal of Poly- And Per-Fluorinated Compounds from Ion Exchange Regenerant Still Bottom Samples in a Plasma Reactor. Environ Sci Technol..

[208] Toxico Watch. Hidden Emissions : A Story from the Netherlands.; 2018. https://zerowasteeurope.eu/wp-content/uploads/2018/11/NetherlandsCS-FNL.pdf.

[209] Aleksandrov K., Gehrmann H.J., Hauser M. (2019). Waste incineration of Polytetrafluoroethylene (PTFE) to evaluate potential formation of per- and Poly-Fluorinated Alkyl Substances (PFAS) in flue gas. Chemosphere..

[210] Taylor P.H., Yamada T., Striebich R.C., Graham J.L., Giraud R.J. (2014). Investigation of waste incineration of fluorotelomer-based polymers as a potential source of PFOA in the environment. Chemosphere..

[211] Meng J., Lu Y., Wang T. (2017). Life cycle analysis of perfluorooctanoic acid (PFOA) and its salts in China. Environ Sci Pollut Res..

[212] 3M. Final Report: Laboratory-Scale Thermal Degradation of Perfluoro-Octanyl Sulfonate and Related. 2003;(May). https://cswab.org/wp-content/uploads/2019/02/Taylor-PFAS-3M-Study-Incineration-PFAS-Degradation2000-Degrees-Fahrenheit-40-seconds-2003.pdf.

[213] Winchell L.J., Ross J.J., Wells M.J.M., Fonoll X., Norton J.W., Bell K.Y. (2021). Per- and polyfluoroalkyl substances thermal destruction at water resource recovery facilities: A state of the science review. Water Environ Res..

[214] Turner LP, Kueper BH, Weber KP. Remediation of Perfluorooctane Sulfonate (PFOS) and Perfluorooctanoic Acid (PFOA) Spiked Sands and Aqueous Film Forming Foam (AFFF) Impacted Soils by Ball Milling. 2019;21:10908.

[215] Yu G., Huang J., Lu M., Cagnetta G., Zhang K. (2017). Mechanochemical mineralization of “very persistent” fluorocarbon surfactants – 6:2 fluorotelomer sulfonate (6:2FTS) as an example. Sci Rep..

[216] Zhang K., Cao Z., Huang J., Deng S., Wang B., Yu G. (2016). Mechanochemical destruction of Chinese PFOS alternative F-53B. Chem Eng J..

[217] Zhang K., Huang J., Yu G., Zhang Q., Deng S., Wang B. (2013). Destruction of perfluorooctane sulfonate (PFOS) and perfluorooctanoic acid (PFOA) by ball milling. Environ Sci Technol..

[218] Yamamoto T., Noma Y., Sakai S.I., Shibata Y. (2007). Photodegradation of perfluorooctane sulfonate by UV irradiation in water and alkaline 2-propanol. Environ Sci Technol..

[219] Jin L., Zhang P., Shao T., Zhao S. (2014). Ferric ion mediated photodecomposition of aqueous perfluorooctane sulfonate (PFOS) under UV irradiation and its mechanism. J Hazard Mater..

[220] Park H., Vecitis C.D., Cheng J., Dalleska N.F., Mader B.T., Hoffmann M.R. (2011). Reductive degradation of perfluoroalkyl compounds with aquated electrons generated from iodide photolysis at 254 nm. Photochem Photobiol Sci..

[221] Yasuoka K., Sasaki K., Hayashi R. (2011). An energy-efficient process for decomposing perfluorooctanoic and perfluorooctane sulfonic acids using dc plasmas generated within gas bubbles. Plasma Sources Sci Technol..

[222] Thangavadivel K., Okitsu K., Owens G., Lesniewski P.J., Nishimura R. (2013). Influence of sonochemical reactor diameter and liquid height on methyl orange degradation under 200 kHz indirect sonication. J Environ Chem Eng..

[223] Committee DN. The Biden Plan to Secure Environmental Justive and Equitable Economic Opportunity. https://joebiden.com/environmental-justice-plan/. Published 2021. Accessed April 5, 2021.

