# Supplementary material for: Sonolysis of per- and poly fluoroalkyl substances (PFAS): A meta-analysis
Source: Ultrason Sonochem. 2022 Feb 7;87:105944. doi: 10.1016/j.ultsonch.2022.105944 (PMC9184745; doi:10.1016/j.ultsonch.2022.105944)
Supplement: Supplementary Data 1 [file mmc1.docx]

Supplementary information

Sonolysis of Per- And Poly Fluoroalkyl Substances (PFAS): A meta-analysis

Tim Sidnell^a^, Richard James Wood^b^, Jake Hurst^c^, Judy Lee^a^, Madeleine J. Bussemaker*^a^

^a^ Department of Chemical and Process Engineering, University of Surrey, Guildford, Surrey GU2 7XH, United Kingdom.

^b^ Institute of Biomedical Engineering, Department of Engineering Science, University of Oxford, Oxford, UK.

^c^ ARCADIS, 1 Whitehall Riverside, Leeds, LS1 4BN, UK, United Kingdom.

*e-mail: [m.bussemaker@surrey.ac.uk](mailto:m.bussemaker@surrey.ac.uk)

Table S1: List of Acronyms used throughout this work and their meanings

| Chemical Name Acronyms | | | |
| --- | --- | --- | --- |
| Acronym | Full name | Structure | |
| CTAB | Cetrimonium bromide | CH_3_(CH_2_)_15_N(CH_3_)_3_Br | |
| FASAs | Perfluoroalkane sulfonamides | C_n_H_2(n-7)_F_17_NO_2-4_S | |
| FTOHs | Fluorotelomer alcohols | CF_3_C_n_F_2n_(CH_2_)_2_OH | |
| HpFOPA | Heptafluoro-3-pentanoic acid | CF_3_F_2_OCF_2_COOH | |
| NDFTOTDA | Nonadecafluoro-3,6,9-trioxatridecanoic acid | CF_3_(CF_2_)_3_O(CF_2_)_2_O(CF_2_)_2_OCF_2_COOH | |
| NFDOHpA | Nanofluoro-3,6-dioxaheptanoic acid | CF_3_O(CF_2_)_2_OCF_2_COOH | |
| NFOHxA | Nanofluoro-5-oxahexanoic acid | CF_3_O(CF_2_)_3_COOH | |
| PDFTODA | Pentadecafluoro-3,6-dioxadecanoic acid | CF_3_(CF_2_)_3_O(CF_2_)_2_OCF_2_COOH | |
| PFAA | Perfluoroalkyl acids | CF_3_(CF_2_)_n_XH | |
| PFAS | Per- and polyfluoroalkyl substance | CF_3_(CF_2_)_n_X | |
| PFBA | Perfluorobutanoic acid | CF_3_(CF_2_)_2_COOH | |
| PFBS | Perfluorobutane sulfonic acid | CF_3_(CF_2_)_3_SO_3_H | |
| PFBX | Perfluorobutan(oate/e sulfonic acid) | CF_3_(CF_2_)_2/3_(COOH/SO_3_H) | |
| PFCA | Perfluorocarboxylic acid | CF_3_(CF_2_)_n_COOH | |
| PFDA | Perfluorodecanoic acid | CF_3_(CF_2_)_8_COOH | |
| PFDoA | Perfluorododecanoic acid | CF_3_(CF_2_)_10_COOH | |
| PFEC | Perfluoroalkyl ether carboxylate | CF_3_(CF_2_)_α_[O(CF_2_)_β_][O(CF_2_)_γ_]…COOH | |
| PFEES | Perfluoro(2-ethoxyethane)sulfonic acid |  | |
| PFES | Perfluoroalkyl ether sulfonate | CF_3_(CF_2_)_α_[O(CF_2_)_β_][O(CF_2_)_γ_]…SO_3_H | |
| PFHpA | Perfluoroheptanoic acid | CF_3_(CF_2_)_5_COOH | |
| PFHS | Perfluorohexane sulfonic acid | CF_3_(CF_2_)_5_SO_3_H | |
| PFHX | Perfluorohexan(oate/e sulfonic acid) | CF_3_(CF_2_)_4/5_(COOH/SO_3_H) | |
| PFHxA | Perfluorohexanoic acid | CF_3_(CF_2_)_4_COOH | |
| PFNA | Perfluorononanoic acid | CF_3_(CF_2_)_7_COOH | |
| PFOA | Perfluorooctanoic acid | CF_3_(CF_2_)_6_COOH | |
| PFOBA | Pentafluoro-3-butanoic acid | CF_3_OCF_2_COOH | |
| PFOS | Perfluorooctane sulfonic acid | CF_3_(CF_2_)_7_SO_3_H | |
| PFOSA | Perfluorooctane sulphonamide | CF_3_(CF_2_)_7_SO_2_NH_2_ | |
| PFOX | Perfluorooctan(oate/e sulfonic acid) | CF_3_(CF_2_)_6/7_(COOH/SO_3_H) | |
| PFPrA | Perfluoropropionic acid | CF_3_CF_2_COOH | |
| PFPeA | Perfluoropentanoic acid | CF_3_(CF_2_)_3_COOH | |
| PFSA | Perfluoroalkyl sulfonic acid | CF_3_(CF_2_)_n_SO_3_H | |
| PTFE | Polytetrafluoroethylene | CF_3_(C_2_F_4_)_n_CF_3_ | |
| SDS | Sodium dodecyl sulfate | CH_3_(CH_2_)_11_SO_4_Na | |
| TDFTODA | Tridecafluoro-3,6,9-trioxadecanoic acid | CF_3_O(CF_2_)_2_O(CF_2_)_2_OCF_2_COOH | |
| UDFDOOA | Undecafluoro-3,6-dioxaoctanoic acid | CF_3_CF_2_O(CF_2_)_2_OCF_2_COOH | |
| UDFOHpA | Undecafluoro-3-oxaheptanoic acid | CF_3_(CF_2_)_3_OCF_2_COOH | |
| Treatment Process Acronyms | | | |
| Acronym | Meaning | | |
| AOP | Advanced Oxidation Process | | |
| EO | Electrochemical Oxidation | | |
| UV | Ultraviolet (light) | | |
| Other Acronyms | | | |
| Acronym | Meaning | | |
| AFFF | Aqueous fire-fighting foam | | |
| CMC | Critical micelle concentration | | |
| FTS | Fluorotelomer Sulfonate | | |
| HPLC-MS | High performance liquid chromatography – mass spectrometry | | |
| IDW | Investigation derived waste | | |
| LH | Langmuir-Hinshelwood (kinetics) | | |
| MM | Michaelis-Menten (kinetics) | | |
| MQ | Milli-Q (water) | | |
| PD | Power Density (Watts L^-1^) | | |
| pKa | Acid dissociation constant | | |
| pg | Picograms | | |
| PS | Picoseconds | | |
| R- | Attached organic region, e.g. R-COOH where R = CF_2_(CF_2_)_6_ in PFOA | | |
| TOC | Total organic Carbon | | |
| Symbols | | | |
| Symbol | Meaning | | Units |
| • | Radical species | | (-) |
| $\propto$ | Proportional to | | (-) |
| C_P_ | Specific heat capacity at constant pressure | | J K^-1^ mol^-1^ |
| C_v_ | Specific heat capacity at constant volume | | J K^-1^ mol^-1^ |
| K_eq_ | Equilibrium partitioning coefficient | | (-) |
| ν | Nu (Frequency) | | kHz |
| P | Power | | Watts |

Table S2: Summary of Papers on the Sonochemical Degradation of PFAS

| Title (Year) | Key Findings | Ref |
| --- | --- | --- |
| Sonochemical Decomposition of Perfluorooctane Sulfonate and Perfluorooctanoic Acid (2005) | 1. Proof of concept: 60% and 98% degradation of 10 mg L^-1^ PFOS (20.0 µM) and PFOA (24.3 µM), respectively, in one hour 2. PFOA degraded faster than PFOS 3. Proposed degradation mechanism:    1. Pyrolytic head group removal at the bubble interface    2. Repeated oxidation and truncation of perfluoro-chain in the bulk liquid 4. Argon is a more effective dissolved gas (faster reaction rate) than air 5. PFAS degradation shows pseudo-first order rate kinetics and half-lives of several minutes 6. Radicals less significant in PFOS degradation than bubble collapse temperature and pressure. No such conclusion could be made for PFOA. | ^1^ |
| Kinetics and Mechanism of the Sonolytic Conversion of the Aqueous Perfluorinated Surfactants, Perfluorooctanoate (PFOA), and Perfluorooctane Sulfonate (PFOS) into Inorganic Products (2008) | 1. Confirms findings 2, 3.1, 5, and 6 in ^1^ 2. Disputes finding 3.2 in ^1^;    1. CO/CO_2_ formation correlates with C_n_F_2n+1_ destruction in the bubble vapour (not the liquid)    2. Degradation generates CF_2_ radicals which then oxidise in the liquid    3. No detection of aqueous short chain production (some in gas phase, <0.1% of initial fluorine) 3. Gaseous short chain PFASs passively diffuse back into the liquid and are destroyed with continued sonolysis 4. Near complete mineralisation of PFOA/S to CO_2_, CO, SO_4_^2-^ and F^-^ 5. PFOA/S well degraded at concentration from 10 nM to 10 µM 6. Suggests the rate is limited (after sorption to the bubble) by intermediate product diffusion into the bubble vapour | ^2^ |
| Enhancement of Perfluorooctanoate and Perfluorooctanesulfonate Activity at Acoustic Cavitation Bubble Interfaces (2008) | 1. Confirms findings 2 and 5 given in ^1^ (finding 1. in ^2^) 2. Disputes finding 6 in ^2^ - Considers that pyrolytic head group cleavage is the rate limiting step, after adsorption 3. A switch between first and zero order kinetics occurs at around 13 µM for PFOA and 25 µM for PFOS 4. Maximum PFAS air-water interfacial concentrations derived 5. PFOS has a greater partitioning coefficient (K_eq_) than PFOA and dominates surface tension in combined systems 6. PFOS is a stronger surfactant and better fits with LH kinetics than PFOA 7. Bubble oscillations enhance PFAS sorption to the bubble | ^3^ |
| Sonochemical Degradation of Perfluorooctane Sulfonate (PFOS) and Perfluorooctanoate (PFOA) in Landfill Groundwater: Environmental Matrix Effects (2008) | 1. Confirms finding 6 given in ^1^ 2. Similarly to ^1^, ^2^, and ^3^, pseudo-first order kinetics occur for combined PFAS/landfill leachate mixtures 3. Organics’ effect on reaction rate $\propto$ concentration, heat capacity, volatility, heat of dissociation and product (e.g., CO_2_, H_2_) dissociation energies 4. Larger organic contaminants have more impact on reaction rate, per mol sorbed to the bubble, however, smaller organics have a greater impact on sonolysis per mol in solution 5. PFOS rate constant is more easily affected by other contaminants than that of PFOA 6. Sonozone treatment aids degradation of PFOA/S in landfill leachate by removing co-organics 7. The Langmuir adsorption constant is a better indicator of surface activity than maximum surface concentration | ^4^ |
| Sonication-assisted photocatalytic decomposition of perfluorooctanoic acid (2009) | 1. Similarly to ^1–4^, degradation follows pseudo first order kinetics 2. TiO_2_ generates PFOA radicals which degrade via O_2_• at pH 4 and OH• at pH 10. 3. F^-^ release is low (5.3% of initial in eight hours) and significant concentrations of short chain PFCAs are generated 4. pH 10 showed the greatest decomposition and pH 7 showed the worst due to catalyst agglomeration 5. Simultaneous sonication alongside UV light is more effective than sequential combinations | ^5^ |
| Perfluorinated Surfactant Chain-Length Effects on Sonochemical Kinetics (2009) | 1. Confirms findings in ^1^, ^2^ and ^3^ - PFCAs have a lower thermal activation energy than PFSAs (which is not greatly affected by chain length) 2. Somewhat disputes finding 6 in ^2^, agrees with finding 7 in ^3^ – Bubble interfacial equilibrium partitioning may only be rate limiting for shorter (<C6) chains 3. Similarly to findings in ^1^-^5^, pseudo-first order kinetics also apply to short chains, however, they are degraded slower 4. An exponential relationship is seen between PFAS chain length and K_eq_. A linear relationship is seen for hydrocarbons 5. Long chains are degraded best at 358 kHz while short chains are degraded best at 610 kHz 6. Long perfluoro chains offer leverage and closely anchor PFAS to the bubble interface. 7. In short chains, head group repulsion dominates over hydrophobicity, hence, maximum bubble interface concentrations are reduced 8. Short chain sulfonates are more easily degraded than carboxylates, while the reverse is true for long chains 9. Stable cavitations encourage faster rectified diffusion and hence quicker collapse | ^6^ |
| Sonochemical Degradation of Perfluorooctanesulfonate in Aqueous Film-Forming Foams (2010) | 1. Confirms findings in ^1^, ^2^ and ^3^;    1. Head group cleavage is the first step in PFOS sonolysis    2. Kinetic order is dependent on concentration and switches from zero to first order with during treatment 2. Similarly to ^4^, high organic concentration has little effect on degradation rate 3. F^-^ release is less than anticipated, while SO_4_^2-^ yield is greater ^2^, indicating a different degradation mechanism for AFFFs, compared to simple aqueous systems 4. Other compounds in the AFFF (including other PFASs) are also decomposed 5. PFOS degradation rate vs dilution follows 5,000x > 500x > 250x > 50,000x, showing competing physical effects 6. Two possible mechanisms for initial head group removal are provided: i) molecular rearrangement and breakage and ii) interaction with protons in solution | ^7^ |
| Sonochemical Degradation of Perfluorooctane Sulfonate (PFOS) and Perfluorooctanoate (PFOA) in Groundwater: Kinetic Effects of Matrix Inorganics (2010) | 1. Agrees with ^3^ - Sonochemical activity is greater than equilibrium surface activity 2. Similarly to finding 5 in ^4^ – co-inorganics reduce PFOS’ pseudo-first order rate more than PFOA’s at 354 and 612 kHz 3. Anions and low pH are suggested to cause inter-bubble repulsion and thus maintain many small cavities of high surface area 4. Anions may impact interfacial water structure and thus water vapour/heat transport to and from the bubble core and thus the core collapse temperature 5. HCO_3_^-^ is the key contributor to negative groundwater effects on sonolysis and can be mitigated by acidification 6. Cations have little effect on degradation rate compared to anions 7. Anion effects are consistent with the Hofmeister series; ClO_4_^-^ significantly enhanced PFOA rate, more so for PFOS, Cl^-^ and NO_3_^-^ gave some improvements and SO_4_^2-^ and HCO_3_^-^ reduced rate constants (SO_4_^2-^ more so for PFOA) 8. pH significant below 3, at which rates were enhanced, more so for PFOS than PFOA, possibly due to enhancement of PFAS attraction to the interface | ^8^ |
| Efficient decomposition of perfluoroether carboxylic acids in water with a combination of persulfate oxidant and ultrasonic irradiation (2012) | 1. 93.5% degradation was achieved for NFDOHA in 24 hours 2. PFEC degradation route suggested to follow a similar path to those of PFCAs and PFSAs proposed in ^1^ - ^8^ 3. Rate enhancement by persulfate, of up 3.9 times, was thought to be due to enhanced de-carboxylation by S_2_O_8_^2-^ and was greatest for short chains 4. S_2_O_8_^2-^ showed almost no enhancement for perfluoroether sulfonates 5. Similarly to finding 4 in ^1^ - sparged argon enhances degradation of NFDOHA (not tested for other species) but reduces fluoride yield 6. Without S_2_O_8_^2-^, kinetics are zero order with 55.8% degraded in 24 hours and 27.7% F^-^ formed, indicating formation of other FCs. Adding S_2_O_8_^2-^ switches reaction to first order NFDOHA degradation and F^-^ release | ^9^ |
| Intensification of sonochemical degradation of ammonium perfluorooctanoate by persulfate oxidant (2014) | 1. Similarly to ^2^ - Degradation is via interfacial head group cleavage followed by multiple pyrolysis reactions in the vapour 2. Similarly to finding 4 in ^9^ - Sulfate radicals (SO_4_•^-^) enhance PFOA sono-intermediate head group removal and thus F^-^ release 3. PFOA shows much faster degradation than PFECs under similar conditions in ^9^, albeit at much higher power 4. High collapse temperatures are critical to enhancing the effects of sulfate radicals 5. Increasing PFOA concentration, while maintaining persulfate concentration, decreases defluorination % 6. Increasing power aids degradation up to a point, after which increased power becomes detrimental 7. MeOH limits the reaction rate by assumed collapse quenching while benzoic acid competes for bubble surface | ^10^ |
| Enhancing decomposition rate of perfluorooctanoic acid by carbonate radical assisted sonochemical treatment (2014) | 1. Agrees with finding 2.2 in ^2^ - degradation is via CF_2_ radical formation (despite different frequency regime and lower destruction) 2. Agrees with finding 4 in ^5^ and conflicts finding 5 in ^8^ - NaHCO_3_ addition and high pH show improved degradation (at much lower frequency) 3. Degradation is improved up to four-fold by carbonate radicals (CO_3_•) up to an maximum NaHCO_3_ concentration, above which the rate is reduced 4. N_2_ sparging has little effect with or without CO_3_• 5. No short chain species detected | ^11^ |
| Effect of sound frequency and initial concentration on the sonochemical degradation of perfluorooctane sulfonate (PFOS) (2015) | 1. Confirms findings in ^1^, ^2^, ^3^, ^6^ - kinetic order switches from pseudo-first to zero order with increased concentration via bubble saturation 2. Confirms findings in ^1^ and ^7^, disputes finding 4 in ^2^ - complete mineralisation not achieved and short species formed as with 3. Disputes findings in ^1^, ^2^, ^3^, ^7^ - head group removal is not the initial step in degradation 4. Disputes findings in ^1^ and ^2^ - OH radical generation correlates well with degradation and high frequency 5. Disputes finding 5 in ^6^ - 1,000 kHz shows highest reaction rate as opposed to around 350 kHz 6. 500 kHz shows better defluorination at high PFOS concentrations whereas 1Mhz works best at low concentrations 7. Suggests that increasing PFAS concentration increases electrostatic inter-bubble repulsion and maintains high bubble populations and reduces coalescence | ^12^ |
| Sonochemical degradation of perfluorinated surfactants: Power and multiple frequency effects (2015) | 1. Similarly to ^1^, ^2^, ^3^, and ^6^    1. PFHA and PFOA show faster degradation than PFHS and PFOS both frequencies tested (358 and 610 kHz)    2. Shorter chains are less well degraded due their reduced K_eq_ but rates enhanced by higher frequency (610 kHz) 2. PFBS degraded faster than PFBA at both frequencies 3. For PFOA/S, a 20 kHz horn augmented the rate of a lone transducer at 202 kHz, but not at 610 kHz 4. Agrees with finding 6 in ^10^ - increasing power linearly enhanced degradation rates, up to a point, after which it had a negative effect on rate 5. Increased power density enhanced degradation of PFHA and PFOA more than PFHS and PFOS, and enhanced of PFBA/S degradation best at 610 kHz 6. Increasing PFAS concentration improved efficiency, possibly by reduced coalescence of charged bubble surfaces 7. PFHA/S were degraded better at 610 kHz than 202 kHz or any combined frequencies 8. PFOA/S degraded better at 358 kHz than at 610 kHz or combined frequencies, at all power densities. 9. Secondary Bjerknes forces increase with power, hence different size bubbles aggregate at different regions in the pressure wave. | ^13^ |
| Enhanced sonochemical degradation of perfluorooctanoic acid by sulfate ions (2015) | 1. Confirms findings in ^1–6^ - PFOA follows pseudo-first order degradation 2. Similarly to ^9^ and ^10^    1. Degradation was enhanced by increasing SO_4_^2-^ concentration    2. Smaller chains may be formed without presence of sulphate 3. Similarly to ^8^ - pH has little effect on degradation, but is slightly reduced at high pH 4. Degradation is completed via sonochemical (at the bubble) and chemical (in the liquid) processes simultaneously 5. Increasing temperature from 25°C to 45°C decreases degradation rate – attributed to reduced surface tension and hence reduced adsorption to the bubble interface | ^14^ |
| Effect of surfactants on the degradation of perfluorooctanoic acid (PFOA) by ultrasonic (US) treatment (2016) | 1. Similarly to ^8^ and ^14^ - high pH had a negligibly negative impact on degradation, since the pH exceeded pKa values, while low pH enhanced the reaction rate 2. Degradation and defluorination rates were modified by the surfactants in the order CTAB (Cationic) > TritonX-100 (Non-ionic) > No surfactant > SDS (Anionic) 3. High CTAB concentration increased PFOA degradation | ^15^ |
| Efficient sonochemical degradation of perfluorooctanoic acid using periodate (2016) | 1. Confirm findings in ^8^, ^14^and ^15^, disputes ^5^ - Low pH enhances degradation and F^-^ release 2. Agrees similar reaction mechanism as in ^2^ and ^10^ 3. Somewhat agrees with ^8^ - Effect of anions on degradation rate follows the Hofmeister series, except Cl^-^ 4. Similarly to ^1^, ^9^ and ^11^ - Gas sparging altered the reaction rate in the order N_2_> Air > O_2_, due to their specific heat ratios 5. Degradation rate changes when adding salts were of the order Br^-^ > no ions added > Cl^-^ > SO_4_^2-^ 6. Increased periodate concentration increases degradation - radical scavengers reduce degradation which proves their effects in this mechanism 7. IO_4_^-^ allows near complete mineralisation (some C2-3 chains formed). IO_4_^-^ does not degrade PFOA without US | ^16^ |
| Sonochemical degradation of perfluorinated chemicals in aqueous film-forming foams (2016) | 1. Agrees with finding 5 in ^12^ - 1 MHz was more effective than 500 kHz by 1.5-3.0 times (by measure of TOC) due to higher radical production 2. Disputes finding 5 in ^7^ - Linear increase in initial Ansul foam concentration encouraged linear increase in F^-^ release 3. 3M foam had a lower energy of degradation, than Ansul, and released SO_4_^2-^ faster, despite having a lower sulfonate concentration 4. 3M foam showed near constant F^-^ release with high concentration, while Ansul foam showed a nonlinear increase 5. At 930x dilution, Ansul F^-^ release was 45% higher than 3M, however the 3M foam’s TOC was degraded 32% faster 6. F^-^ release and TOC indicate PFAS degradation occurs during bubble saturation while F^-^ release occurs at sub-saturation conditions 7. F^-^ release and TOC removal show zero order kinetics, whereas SO_4_^2-^ shows switch to first order kinetics 8. For both foams, pH decreased during sonication and short chains were generated 9. PFOA degraded in the Ansul foam but not in the 3M foam, despite similar initial concentrations | ^17^ |
| Effect of chemical structure on the sonochemical degradation of perfluoroalkyl and polyfluoroalkyl substances (PFASs) (2016) | 1. Confirms findings in ^1^, ^2^, ^3^ and ^6^ - PFOA more easily degraded than PFOS due to sulfonate group 2. Confirms findings in ^3^, ^7^ and ^12^ - Zero order kinetics occur at high initial concentrations due to bubble saturation 3. Confirms findings in ^6^ - ≤C4 PFCA and PFSA chains are degraded slower than ≥C5 chains 4. Confirms findings in ^1^ and ^12^ - TOC, F^-^ and SO_4_^2-^ release indicate head group cleavage, oxidation of SO_4_^2-^ and generation of short species from PFOA/S 5. Disputes several others works - PFOA/S degradation showed an initial lag phase of around 30 minutes before defluorination occurred 6. 6:2 FTS chain is less fluorinated and hence has less electron withdrawing capability, which makes the SO_4_^2-^ group more polarised compared to PFOS 7. Higher F^-^ release for PFEEs than PFOS and PFBS may be due to susceptibility to OH radical attack 8. Polyfluorinated compounds degrade slower than perfluorinated species of similar chain length 9. pH decreased with sonication to PFOA and particularly for PFOS | ^18^ |
| Combined sonochemical and short-wavelength UV degradation of hydrophobic perfluorinated compounds (2017) | 1. Agrees with findings in ^1^, ^2^ and ^3^ - PFOA believed to be degraded at interface, primarily via thermal means 2. Agrees with findings in ^1^, ^3^, and ^12^ - Short chain generation expected, but disputes complete mineralisation ^1^, ^2^ due to partitioning to the bulk gas 3. Agrees with findings in ^6^ and ^13^ - Shorter chain PFASs are less volatile and degrade less well 4. Similarly to findings in ^5^ - Degradation appears pseudo first order for PFOA and PFPrA, PFO is zero order after 20 minutes 5. Hydrophobic compounds were better degraded by US than UV, but combined UV enhances defluorination and TOC removal and hence US+UV improves degradation by reducing intermediate concentrations 6. PFOA defluorination follows UV<US<US+UV, for perfluoropropanoic acid it follows US<UV<UV+US and defluorination % follows PFO>PFOA>PFPrA under US+UV 7. Carbonyl groups in PFOA and PFPrA absorb the specific wavelength of UV light used | ^19^ |
| Treatment of perfluorooctane sulfonic acid (PFOS) using a large-scale sonochemical reactor (2017) | 1. Disputes with finding 7 in ^8^    1. NaCl addition reduced F^-^ release but enhanced SO_4_^2-^ release. Na_2_CO_3_ also reduced F^-^ release and was believed to be due to scavenging of OH radicals.    2. NaHCO_3_ addition enhanced SO_4_^2-^ release and TOC removal 2. Similar to ^8^, ^14^, ^15^, ^16^ disputes ^5^ - TOC reduction, F^-^ release and SO_4_^2-^ release all peak at 4 and reduced below pH 4. 3. Similar to ^13^ - Single frequency was more efficient for PFOS degradation than dual frequency 4. F^-^ release increased proportionally to the initial PFOS concentration, however, SO_4_^2-^ and TOC release followed 2.6 > 5.3 > 0.32 mM. 5. %F^-^ release, %SO_4_^2-^ release and %TOC removal decreased with increasing initial PFOS concentration 6. Dual frequency (500 + 1,000 kHz) frequency enhanced fluoride release by 19% and TOC by 233% against 1,000 kHz alone. However, SO_4_^2-^ release was unaffected. 7. TOC was most energetically efficient at dual frequency and F^-^ and SO_4_^2-^ release were most efficient at single frequency. 8. Authors suggest that dual frequency operation increases bubble coalescence | ^20^ |
| Sono-chemical treatment of per- and poly-fluoroalkyl compounds in aqueous film-forming foams using a large-scale multi-transducer dual-frequency based acoustic reactor (2018) | 1. Confirms findings in ^8^, ^14^, ^15^, ^16^ - Ansul foam SO_4_^2-^ release is improved at pH 6.5 < pH 3 < pH4 (at 100x dilution) 2. Disagrees finding 3 in ^17^ - 3M foam was harder to degrade than Ansul foam 3. Disputes findings in ^7^ - F^-^ release was inversely proportional to dilution, TOC removal appeared proportional and SO_4_^2-^ release followed 25x > 100x > 10x > 100x 4. % F^-^ release was similar for both foams despite Ansul having near 2x the total organic fluorine (TOF) level of 3M 5. Ansul foam F^-^ and SO_4_^2-^ release follow zero order for 13 hours but TOC release quickly changes to first order 6. For both foams F^-^ and SO_4_^2-^ release were highest using dual frequency sonication 7. For the 3M foam, >C6 chains (both PFCAs and PFSAs) decreased and with time while ≤6C species and FTS decreased initially (2 hr) then increased (13 hr) at 500x dilution 8. Hydrocarbons in AFFF predicted to degrade via hydroxyl radical reaction | ^21^ |
| Autocatalytic degradation of perfluorooctanoic acid in a permanganate-ultrasonic system (2018) | 1. Confirms findings in ^1^ and ^9^ - Argon increases PFOA degradation rate (+ ≈8%) and defluorination rate (+ 60%) compared to air. Oxygen reduced both rates. 2. Confirms findings in ^10^ and ^13^ - Power enhanced degradation rate nonlinearly 3. Confirms findings in ^8^, ^14^, ^15^, ^16^ and ^21^ - Optimal pH is ≈4 4. Somewhat agrees with ^1^, [5] and [9] - Ultra-short chain formation detected, however, without mid-length chains detected 5. Conflicts finding 6 in ^1^ - Agrees minor role of radicals in degradation, however, tert-butyl alcohol and humic acid addition enhanced reaction and defluorination rates 6. Conflicts with finding 6 in ^8^ – Cations Cu (II) and Fe (II & III) reduced reactions rates by forming complexes with PFOA 7. Conflicts with ^14^ - Increased temperature (30 to 50°C) enhanced reaction rates 8. PFOA reaction rate was non-linearly enhanced by permanganate (MnO_4_^-^) addition but linearly enhanced by the resultant MnO_2_ formation. 9. US alone generated low fluoride release indicating incomplete degradation. PFOA degradation was pseudo first order while fluoride release was pseudo zeroth order 10. Synergistic effects of permanganate addition on fluoride release were greater than the combination of US and permanganate in isolation 11. Permanganate theorised to reduce cavitation threshold by acting as a nucleation point | ^22^ |
| Kinetic model for sonolytic degradation of non-volatile surfactants: Perfluoroalkyl substances (2019) | 1. Confirms findings in ^1^, ^2^, ^3^, ^6^ and ^18^ -    1. PFOA more easily degraded than PFOS due to thermal requirements. However, PFOS may dominate degradation over PFOA at high concentrations, due to its greater K_eq_    2. Switch between first order and zero order kinetics occurs due to bubble saturation at around 24 µMolar 2. Confirms findings in ^2^ and ^6^ (disputes ^3^) - Diffusion into the interface is the rate limiting step 3. Disputes findings in ^1^ and agrees with ^2^ - Short chain formation is not observed and degradation is via a sonochemical intermediate degraded in the gas or interfacial phase 4. Disputes findings in ^1^, ^2^, ^3^, ^7^ - Head group cleavage not thought to be the initial degradation step 5. Disputes findings in ^3^ and ^4^ - Modelling the bubble as a catalyst thought to be more accurate than Langmuir-Hinshelwood kinetics 6. Around 10x the number of bubbles reach the desired temperature for PFOA degradation compared to PFOS 7. pH is lowered during treatment and with increasing initial PFAS concentrations, hence pH reduction during treatment decreased with increasing initial concentration 8. OH• formation barely limited by PFAS concentration suggesting that O_2_ gas and water can still diffuse into the bubble at high PFAS concentrations 9. NO_2_^-^ concentration increases with time then drops. NO_3_^-^ concentration increases exponentially. Both are likely due to the oxidation of air in solution during treatment. 10. Results suggest that temperature of 1,600°C can sometimes be reached at core, while the interface can reach 350°C | ^23^ |
| Kinetics and mechanism of low-frequency ultrasound driven elimination of trace level aqueous perfluorooctane sulfonic acid and perfluorooctanoic acid (2019) | 1. Confirms findings in ^1^, ^2^, ^3^, ^6^, ^18^ and ^23^ that:    1. PFOA degraded faster than PFOS    2. Switch between first order and zero order kinetics occurs with increasing concentration 2. Confirms findings in ^1^, [5], [9], [16], [17], [21] and ^22^ - PFOA and other short chains are formed from PFOS sonication 3. Confirms findings in ^1^ and ^22^ - Radicals play a minor role in degradation (compared to interfacial pyrolysis) by scavenging radicals using tertbutyl alcohol 4. Confirms finding 5 in ^14^, disputes finding 7 in ^22^ - Degradation rate showed an optimum value at 20°C (in the range 10°C - 40°C) 5. Confirms findings in ^10^, ^13^ and ^22^ - Power density enhanced degradation rate nonlinearly (optimum at 3,750 W L^-1^ in range 1,500 - 4,100 W L^-1^ ) 6. General agreement with findings in ^8^, ^14^, ^15^, ^16^ and ^21^ - Optimal pH 2 was optimum in range 2-7 7. Disputes ^1^, ^2^, ^3^, ^6^, ^18^ and ^23^ –    1. Reaction rate decreased with increasing concentration above 180 and 220 pM for PFOA and PFOS, respectively    2. Switch between pseudo-first and zero order kinetics occurs much lower concentration (≈ 400 pM) than previous work. 8. Disputes finding 4 in ^6^ - Argues that short chain intermediates are absorbed by the bubble and degraded faster than long chains in the core due to high Henry’s constants 9. Power theorised to enhance number of active bubbles, increase collapse temperature, and mixing rate. | ^24^ |
| Synergistic degradation of PFAS in water and soil by dual-frequency ultrasonic activated persulfate (2020) | 1. Proof of concept for pulsed ultrasound (1s on 1s off) in a nitrogen agitated soil slurry 2. Confirms findings in ^1^, ^2^, ^3^, ^6^, ^18^, ^23^ and ^24^ - PFOA degraded faster than PFOS (Fluoride % release is of the order PFOA > 6:2 FTS > PFOS) 3. Confirms findings in ^1^, [5], [9], [16], [17], [21], ^22^ and ^24^ - PFOA and 6:2 FTS generated (possibly form larger molecules such as EtFOSAA and 8:2 FTS in the soil) 4. Confirms findings in ^10^, ^13^, ^22^ and ^24^ - Power density enhanced degradation rate nonlinearly – power proposed to enhance reaction rate due to enhanced bubble collapse temperature, bubble concentration, radical concentration, pyrolysis rate and mass transport. 5. Confirms findings in ^8^, ^14^, ^15^, ^16^, ^21^ and ^24^ - Low pH shows high reaction rates. Theorised to be due to OH reaction with SO_4_•^-^ to form OH• which live for less time as they are non-targeted and increased potential of OH• at low pH. 6. Confirms findings in ^24^ - pH lowered during treatment 7. PFOA degradation follows persulfate (PS) addition < 20 kHz (550 W) < 43 kHz (250 W) < 20 kHz + 43 kHz < PS + 20 kHz < PS + 43 kHz << PS + 20 kHz + 43 kHz 8. Both pyrolytic and chemical degradation effects reported, 43 kHz ultrasound generated less heat than the 20 kHz ultrasound 9. By product HF gas generated and captured using NaOH solution | ^25^ |
| Sonochemical degradation of poly- and perfluoroalkyl substances – A review (2020) | 1. First review of solely PFAS sonolysis research - effects of various ultrasonic and chemical parameters 2. Non-linear effects of power on reaction rate, seen at high power densities ^10^, ^13^, ^22^ and ^24^, may be due to scattering/blocking of sound wave energy by large bubbles as well as enhanced bubble coalescence and hence reduced collapse pressure 3. Reductions in reaction rate at higher temperatures may be due to enhanced bubble coalescence (as well as reduced surface tension and PFAS adsorption ^14^) 4. Sonication appears well suited to both in- and ex-situ treatment of PFAS. However, much works is needed for a fully parametrically optimised large scale PFAS sonolysis reactor 5. Reactor design should also consider costs, combinations of treatments, and effects of co-contaminants in real treatment waters | ^26^ |
| Ultrasonic degradation of perfluorooctane sulfonic acid (PFOS) correlated with sonochemical and sonoluminescence characterisation (2020) | 1. Confirms findings in ^1^, ^5^, ^9^, ^22^ and ^24^ – Low quantities of short chains (<1% of initial PFAS concentration) were formed during sonication and degraded with time 2. Confirms findings in ^24^ and ^25^ – pH was lowered during treatment 3. Confirms trend in ^6^ - Reaction rate of PFOS follows frequency dependence in the order: r_402.6 kHz_ > r_500 kHz_ > r_996.1 kHz_ >> r_44 kHz_ 4. PFOS appears to be primarily degraded via a sonochemical route (as opposed to a thermal one) based on sonoluminescence, sonochemiluminescence and dosimetry correlation 5. Theorises that headgroup cleavage occurs through release of solvated electron from the surface of collapsing bubble – supported in other PFAS treatment technologies 6. Ultrasound is highly cost effective (g degraded per kWh) compared to other technologies, second only to plasma treatment - which incurs high short chain production 7. Demonstrated little variation (±1%) in fluoride concentration measured via combustion ion chromatography and ion selective electrodes | ^27^ |
| Theoretical evaluation of chemical and physical feasibility of an in situ ultrasonic reactor for remediation of groundwater contaminated with per‐ and polyfluoroalkyl substances (2020) | 1. Theoretical proof of concept using sonolysis to treat PFAS in-situ, in subsurface groundwater and stop the plume progressing 2. Treatment efficiency was improved when treating concentrated source zones as opposed to downstream zones in the PFAS plume 3. Between 1-491 days required for treatment time– dependent on initial site concentration, PFAS composition, number of reactors used, well velocity, aquifer hydraulic conductivity and target concentrations 4. In-situ PFAS precursor pre-treatments (such as oxidation) could reduce treatment time but releases greater emissions 5. 4:2 FTS poses greatest challenge to treatment of the 14 PFAS and pre-cursors assessed 6. Treatment costs for the sites investigated varied from $330,000 - $1,220,000 which also depended on the target depth 7. Bore wells used in the treat treatment have greater emissions than operation of the transducers for a year, so minimising number of wells is paramount | ^28^ |
| A sustainability assessment of an in situ ultrasonic reactor for remediation of PFAS‐contaminated groundwater (2020) | 1. In situ PFAS sonication in groundwater has a theoretically lower environmental impact than pump and treat and is appropriate to low hydraulic conductivity plumes 2. PFBA treatment requires use of several reactors (due to low reaction rate) 3. Increasing the number of reactors used for a given plume reduces the number of wells required 4. In situ treatment theoretically reduces habitat disruption by limiting project footprint compared to pump and treat | ^29^ |
| Frequency-dependent sonochemical degradation of perfluoroalkyl substances and numerical analysis of cavity dynamics (2021) | 1. Confirms findings in ^1^, ^2^, ^3^, ^6^, ^18^, ^23^, ^24^ and ^25^ - PFOA degraded faster than PFOS, since fewer bubbles are predicted to reach the require collapse temperature 2. Confirms findings in ^10^, ^13^, ^22^ and ^24^ – Increasing power density enhanced PFOX degradation rates nonlinearly 3. Confirms finding 7 in ^22^, conflicts with findings in ^14^ and ^24^ – Increased temperature (14.5-30.7°C) enhanced PFOX reaction rate 4. Disputes findings in ^8^, ^14^, ^15^, ^16^, ^21^, ^24^ and ^26^ – High pH provided a faster PFOX reaction rate than low pH, however, the inverse was seen when sparging with argon 5. Disputes findings in ^1^, ^5^, ^9^, ^22^, ^24^ and ^27^ (confirms findings in ^2^) – No short chains formed during sonication of PFOX 6. Disputes findings in ^1^, ^9^ and ^22^ – No sparged gas, i.e. only dissolved air, provided a faster PFOX reaction rate than sparged argon (effect of gases dependent on PFAS type) 7. Disputes findings in ^6^ and ^27^ – PFOA reaction rate followed 575 kHz > 1,140 kHz > 860 kHz and that of PFOS followed 1,140 kHz > 575 kHz > 860 kHz (500 ml) 8. Rate order shown to be dependent on reactor power density 9. Sonochemical by-products and intermediates can alter interactions of PFOX with the bubble interface 10. The difference in the degradation rates of PFOA and PFOS reduces with increasing applied frequency and decreasing reactor volume 11. Use of Arrhenius equation suggests that the activation energy of PFOS is double that of PFOA | ^30^ |
| Power density modulated ultrasonic degradation of perfluoroalkyl substances with and without sparging Argon (2021) | 1. Confirms findings in ^1^, ^2^, ^3^, ^6^, ^18^, ^23^, ^24^, ^25^ and ^30^ - PFOA degraded faster than PFOS due to higher require collapse temperature 2. Confirms finding 8 in ^30^ - Rate order switched, from zero to pseudo-first order, based on increased power density 3. Disputes findings in ^16^ – periodate did not enhance reaction rates. Difference in observation may be due to lower concentration used here 4. Disputes findings in ^1^, ^5^, ^9^, ^22^, ^24^ and ^27^ but agrees with ^2^ and ^30^ – No short chains formed during sonication of PFOX 5. Decreasing the liquid volume resulted in an exponential increase in calorimetric power density 6. Increasing power density has non-linear increase on degradation rate 7. Argon sparging enhanced PFOX degradation rates at low powers 8. Oxidant addition altered PFOA degradation rate in the order r_chlorate_ ≥ r_iodate_ > r_persulfate_ > r_perchlorate_ > r_no oxidant_ ≥ r_periodate_ and that of PFOS followed r_iodate_ > r_chlorate_ = r_persulfate_ > r_perchlorate_ > r_no oxidant_ > r_periodate_ 9. Suggests that PFOS must be degraded thermally while PFOA can be degraded radically | ^31^ |
| Sonolytic destruction of Per- and polyfluoroalkyl substances in groundwater, aqueous Film-Forming Foams, and investigation derived waste (2021) | 1. Confirms findings in ^1^, ^2^, ^3^ and ^6^ - PFSAs degraded slower than equivalent length PFCAs 2. Confirms findings in ^6^, ^13^ and ^19^ - Shorter chain PFASs are less hydrophobic and degrade slower than longer chains 3. Confirms findings in ^10^, ^13^, ^22^, ^24^ and ^30^ - Nonlinear effect of increasing power density on reaction rate 4. Proof of degradation of novel PFAS - hexafluoropropylene oxide dimer acid (HFPO-DA) and 6:2 fluorotelomer sulfonamidoalkyl betaine (6:2 FTAB) 5. HFPO-DA also appears to follow zero order kinetics at high concentrations (23 mg L^-1^) and pseudo-first order at low concentrations (900 g µL^-1^) 6. High total dissolved solids reduced the degradation rates some PFAS, however others were enhanced by low suspended solids, compared to deionised water 7. No disinfection by-products formed although unspecified precipitates were formed during treatment of high suspended solid material 8. Degradation of novel ether containing species 9. 70-99% removal of all PFAS in the IDW and 61-99.6% in diluted AFFF 10. More fluoride detected than theoretically predicted– some other PFAS must have been present and degraded. F^-^ release and PFAS removal may be different rate orders 11. Power density effects were specific to the individual PFAS and the concentrations used (e.g., less effect on PFSAs than PFCAs) 12. Use of a closed reactor enhanced reaction rates compared to an open one, hence, the system pressure may affect availability of PFAS in the system 13. Moderate salt concentration may enhance PFAS hydrophobicity but too high a concentration may cause bubble agglomeration, reduced radical concentrations, competitive use of sonochemical activity and PFAS sorption to the reactor walls | ^32^ |
| Ineffectiveness of ultrasound at low frequency for treating per- and polyfluoroalkyl substances in sewage sludge (2022) | 1. 20 kHz ultrasound and persulfate addition ineffective at degrading PFAS in a solid sewage sludge (bar PFBA which was slightly degraded) 2. Some PFCAs increased in concentration in the liquid phase by sonication, likely due to disruption of sludge flocks, which was enhanced by persulphate addition 3. Sonication increased the soluble chemical oxygen demand in primary, secondary, and combined primary-secondary sludges, more so in the secondary sludge 4. Soluble chemical oxygen demand was barely affected by increasing treatment temperature from 40°C to 50°C 5. PFAAs and precursors were found in higher concentrations in the sludge solid phase than the liquid phase 6. Sonication suggested to increase PFAS pollution risk when treating sewage sludge 7. Use of higher frequencies may prove suitable for ultrasonic PFAS treatment in sludge | ^33^ |

Table S3: Data used in sonolysis meta-analysis

| PFAS | Ultrasonic Parameters | | | | | | | | | | | | | | | | | | | | | | | | | | PFAS Rate constant | | | | | | | | | | | | | F^-^ rate constant | | | | | | | | | | Ref | |
| --- | --- | --- | --- | --- | --- | --- | --- | --- | --- | --- | --- | --- | --- | --- | --- | --- | --- | --- | --- | --- | --- | --- | --- | --- | --- | --- | --- | --- | --- | --- | --- | --- | --- | --- | --- | --- | --- | --- | --- | --- | --- | --- | --- | --- | --- | --- | --- | --- | --- | --- | --- |
|  | ν  (kHz) | | | C_0_  (mg L^-1^) | | | | | | | | | PD  (W L^-1^) | | | | | | | | | Dissolved gas | | | | | 0^th^ order  (nM min^-1^) | | | | | | | Pseudo-1^st^ order  (10^-3^ min^-1^) | | | | | | 0^th^ order  (µM min^-1^) | | | | | Pseudo-1^st^ order  (x10^-3^ min^-1^) | | | | |  |  |
| PFOA | 20 | | | 20.0 | | | | | | | | | 6,000 | | | | | | | | | Air | | | | | - | | | | | | | - | | | | | | 0.38 | | | | | - | | | | | ^10^ | |
|  | 20 | | | 20 + 2,702.2 K_2_S_2_O_8_ | | | | | | | | | 6,000 | | | | | | | | | Air | | | | | - | | | | | | | - | | | | | | 0.67 | | | | | - | | | | | ^10^ | |
|  | 40 | | | 49.7 +Sol-gel TiO_2_ | | | | | | | | | 166.7 + 5.3 UV light | | | | | | | | | O2 | | | | | - | | | | | | | 2.20 | | | | | | - | | | | | 0.00 | | | | | ^5^ | |
|  | 40 | | | 50.0 | | | | | | | | | 150 | | | | | | | | | Air | | | | | - | | | | | | | 1.20 | | | | | | - | | | | | - | | | | | ^11^ | |
|  | 40 | | | 70.4 | | | | | | | | | 500 | | | | | | | | | N_2_ | | | | | - | | | | | | | 9.20 | | | | | | - | | | | | - | | | | | ^16^ | |
|  | 40 | | | 70.4 + 0.03 IO_4_ | | | | | | | | | 500 | | | | | | | | | N_2_ | | | | | - | | | | | | | 22.2 | | | | | | - | | | | | - | | | | | ^16^ | |
|  | 40 | | | 50.0 + 2,520 NaHCO_3_ | | | | | | | | | 150 | | | | | | | | | N_2_ | | | | | - | | | | | | | 24.0 | | | | | | - | | | | | - | | | | | ^11^ | |
|  | 200 | | | 10.0 | | | | | | | | | 3,333 | | | | | | | | | Air | | | | | - | | | | | | | 15.5 | | | | | | - | | | | | - | | | | | ^1^ | |
|  | 200 | | | 10.0 | | | | | | | | | 3,333 | | | | | | | | | Ar | | | | | - | | | | | | | 32.0 | | | | | | - | | | | | - | | | | | ^1^ | |
|  | 354 | | | 7.11 | | | | | | | | | 250 | | | | | | | | | Ar | | | | | 39.0 | | | | | | | 18.4 | | | | | | - | | | | | - | | | | | ^3^ | |
|  | 354 | | | 0.103 | | | | | | | | | 250 | | | | | | | | | Ar | | | | | - | | | | | | | 41.0 | | | | | | - | | | | | - | | | | | ^2^ | |
|  | 354 | | | 95.3 | | | | | | | | | 250 | | | | | | | | | Ar | | | | | 1022.0 | | | | | | | - | | | | | | - | | | | | - | | | | | ^3^ | |
|  | 618 | | | 4.97 | | | | | | | | | 250 | | | | | | | | | Ar | | | | | - | | | | | | | 36.0 | | | | | | - | | | | | 0.30 | | | | | ^2^ | |
| PFOS | 25 | | | 5.00 | | | | | | | | | - | | | | | | | | | Ar | | | | | - | | | | | | | - | | | | | | 0.10 | | | | | - | | | | | ^12^ | |
|  | 44 | | | 10.0 | | | | | | | | | 100 | | | | | | | | | Air | | | | | 0.0 | | | | | | | 0.00 | | | | | | 0.00 | | | | | 0.00 | | | | | ^27^ | |
|  | 200 | | | 10.0 | | | | | | | | | 3,333 | | | | | | | | | Ar | | | | | - | | | | | | | 16.0 | | | | | | - | | | | | - | | | | | ^1^ | |
|  | 200 | | | 10.0 | | | | | | | | | 3,333 | | | | | | | | | Air | | | | | - | | | | | | | 6.8 | | | | | | - | | | | | - | | | | | ^1^ | |
|  | 354 | | | 1.04 | | | | | | | | | 250 | | | | | | | | | Ar | | | | | - | | | | | | | 28.0 | | | | | | - | | | | | - | | | | | ^3^ | |
|  | 354 | | | 106 | | | | | | | | | 250 | | | | | | | | | Ar | | | | | 1150.0 | | | | | | | - | | | | | | - | | | | | - | | | | | ^3^ | |
|  | 354 | | | 0.104 | | | | | | | | | 250 | | | | | | | | | Ar | | | | | - | | | | | | | 27.0 | | | | | | - | | | | | - | | | | | ^2^ | |
|  | 400 | | | 10.0 | | | | | | | | | 100 | | | | | | | | | Air | | | | | 133.0 | | | | | | | 1.30 | | | | | | 2.10 | | | | | 1.40 | | | | | ^27^ | |
|  | 500 | | | 5.00 | | | | | | | | | - | | | | | | | | | Ar | | | | | - | | | | | | | - | | | | | | 0.70 | | | | | - | | | | | ^12^ | |
|  | 500 | | | 50.0 | | | | | | | | | - | | | | | | | | | Ar | | | | | - | | | | | | | - | | | | | | 3.60 | | | | | - | | | | | ^12^ | |
|  | 500 | | | 260 | | | | | | | | | - | | | | | | | | | Ar | | | | | - | | | | | | | - | | | | | | 7.00 | | | | | - | | | | | ^12^ | |
|  | 500 | | | 10.0 | | | | | | | | | 100 | | | | | | | | | Air | | | | | 108.0 | | | | | | | 1.30 | | | | | | 1.90 | | | | |  | | | | | ^27^ | |
|  | 1,000 | | | 5.00 | | | | | | | | | - | | | | | | | | | Ar | | | | | - | | | | | | | - | | | | | | 0.90 | | | | | 0.00 | | | | | ^12^ | |
|  | 1,000 | | | 50.0 | | | | | | | | | - | | | | | | | | | Ar | | | | | - | | | | | | | - | | | | | | 0.10 | | | | | - | | | | | ^12^ | |
|  | 1,000 | | | 230 | | | | | | | | | - | | | | | | | | | Ar | | | | | - | | | | | | | 0.00 | | | | | | 0.00 | | | | | - | | | | | ^12^ | |
|  | 618 | | | 5.38 | | | | | | | | | 250 | | | | | | | | | Ar | | | | | - | | | | | | | 16.0 | | | | | | - | | | | | - | | | | | ^2^ | |
|  | 1,000 | | | 10.0 | | | | | | | | | 100 | | | | | | | | | Air | | | | | 106.0 | | | | | | | 6.80 | | | | | | - | | | | | - | | | | | ^27^ | |
| PFOA & PFOS | 354 | | | 0.10 & 0.10 | | | | | | | | | 250 | | | | | | | | | Ar | | | | | - | | | | | | | 0.047 & 0.024 | | | | | | - | | | | | - | | | | | ^8^ | |
|  | 612 | | | 0.10 & 0.10 | | | | | | | | | 250 | | | | | | | | | Ar | | | | | - | | | | | | | 0.008 & 0.008 | | | | | | - | | | | | - | | | | | ^8^ | |
|  | 358 | | | (4.11 & 5.00) x10^-5^ | | | | | | | | | 333 | | | | | | | | | Ar | | | | | - | | | | | | | 0.057 & 0.040 | | | | | | - | | | | | - | | | | | ^13^ | |
|  | 610 | | | (4.11 & 5.00) x10^-5^ | | | | | | | | | 333 | | | | | | | | | Ar | | | | | - | | | | | | | 0.043 & 0.029 | | | | | | - | | | | | - | | | | | ^13^ | |
|  | 202 | | | (4.11 & 5.00) x10^-5^ | | | | | | | | | 250 | | | | | | | | | Ar | | | | | - | | | | | | | 0.020 & 0.010 | | | | | | - | | | | | - | | | | | ^13^ | |
|  | 20 + 202 | | | (4.11 & 5.00) x10^-5^ | | | | | | | | | 250 | | | | | | | | | Ar | | | | | - | | | | | | | 0.027 & 0.013 | | | | | | - | | | | | - | | | | | ^13^ | |
|  | 610 | | | (4.11 & 5.00) x10^-5^ | | | | | | | | | 250 | | | | | | | | | Ar | | | | | - | | | | | | | 0.034 & 0.020 | | | | | | - | | | | | - | | | | | ^13^ | |
|  | 20 + 610 | | | (4.11 & 5.00) x10^-5^ | | | | | | | | | 250 | | | | | | | | | Ar | | | | | - | | | | | | | 0.037 & 0.021 | | | | | | - | | | | | - | | | | | ^13^ | |
| PFHA & PFHS | 202 | | | 0.117 & 0.092 | | | | | | | | | 250 | | | | | | | | | Ar | | | | | - | | | | | | | 0.019 & 0.012 | | | | | | - | | | | | - | | | | | ^6^ | |
|  | 358 | | | 0.117 & 0.092 | | | | | | | | | 250 | | | | | | | | | Ar | | | | | - | | | | | | | 0.039 & 0.030 | | | | | | - | | | | | - | | | | | ^6^ | |
|  | 610 | | | 0.117 & 0.092 | | | | | | | | | 250 | | | | | | | | | Ar | | | | | - | | | | | | | 0.036 & 0.022 | | | | | | - | | | | | - | | | | | ^6^ | |
|  | 202 | | | 0.101 & 0.092 | | | | | | | | | 333 | | | | | | | | | Ar | | | | | - | | | | | | | 0.025 & 0.016 | | | | | | - | | | | | - | | | | | ^13^ | |
|  | 610 | | | 0.101 & 0.092 | | | | | | | | | 250 | | | | | | | | | Ar | | | | | - | | | | | | | 0.036 & 0.022 | | | | | | - | | | | | - | | | | | ^13^ | |
|  | 610 | | | 0.101 & 0.092 | | | | | | | | | 333 | | | | | | | | | Ar | | | | | - | | | | | | | 0.034 & 0.027 | | | | | | - | | | | | - | | | | | ^13^ | |
|  | 1060 | | | 0.117 & 0.092 | | | | | | | | | 250 | | | | | | | | | Ar | | | | | - | | | | | | | 0.022 & 0.012 | | | | | | - | | | | | - | | | | | ^6^ | |
| PFBA & PFBS | 202 | | | 0.101 & 0.090 | | | | | | | | | 250 | | | | | | | | | Ar | | | | | - | | | | | | | 0.007 & 0.013 | | | | | | - | | | | | - | | | | | ^6^ | |
|  | 358 | | | 0.101 & 0.090 | | | | | | | | | 250 | | | | | | | | | Ar | | | | | - | | | | | | | 0.012 & 0.018 | | | | | | - | | | | | - | | | | | ^6^ | |
|  | 610 | | | 0.101 & 0.090 | | | | | | | | | 250 | | | | | | | | | Ar | | | | | - | | | | | | | 0.017 & 0.023 | | | | | | - | | | | | - | | | | | ^6^ | |
|  | 1060 | | | 0.101 & 0.090 | | | | | | | | | 250 | | | | | | | | | Ar | | | | | - | | | | | | | 0.008 & 0.009 | | | | | | - | | | | | - | | | | | ^6^ | |
|  | 202 | | | 0.101 & 0.090 | | | | | | | | | 250 | | | | | | | | | Ar | | | | | - | | | | | | | 0.007 & 0.013 | | | | | | - | | | | | - | | | | | ^13^ | |
|  | 610 | | | 0.101 & 0.090 | | | | | | | | | 250 | | | | | | | | | Ar | | | | | - | | | | | | | 0.017 & 0.017 | | | | | | - | | | | | - | | | | | ^13^ | |
|  | 610 | | | 0.101 & 0.090 | | | | | | | | | 333 | | | | | | | | | Ar | | | | | - | | | | | | | 0.021 & 0.021 | | | | | | - | | | | | - | | | | | ^13^ | |
| PFAS/  AFFF brand | | | Ultrasonic Parameters | | | | | | | | | | | | | | | | | | | | | | | | | | | | | | | | | | | | | | | | | 0^th^ order F^-^ rate constant  (µM min^-1^) | | | | | | Ref | |
|  |  |  | ν  (kHz) | | | | C_0_  (mg L^-1^) | | | | | | | Reactor volume (L) | | | | | | | Reactor shape | | | | | | | | Transducer mounting | | | | | | | Dissolved gas | | | | | | | |  |  |  |  |  |  |  |  |
| PFOS | | | 25 | | | | 5.0 | | | | | | | 12.0 | | | | | | | Cubic | | | | | | | | Sidewall | | | | | | | Argon | | | | | | | | 0.061 | | | | | | ^12^ | |
|  |  |  | 500 | | | | 5.0 | | | | | | | 12.0 | | | | | | | Cubic | | | | | | | | Sidewall | | | | | | | Argon | | | | | | | | 0.728 | | | | | | ^12^ | |
|  |  |  | 1,000 | | | | 5.0 | | | | | | | 4.50 | | | | | | | Cylindrical | | | | | | | | Base | | | | | | | Argon | | | | | | | | 0.906 | | | | | | ^12^ | |
| 3M | | | 500 | | | | 930 | | | | | | | 12.0 | | | | | | | Cubic | | | | | | | | Sidewall | | | | | | | Argon | | | | | | | | 0.430 | | | | | | ^17^ | |
|  |  |  | 1,000 | | | | 930 | | | | | | | 4.50 | | | | | | | Cylindrical | | | | | | | | Base | | | | | | | Argon | | | | | | | | 1.50 | | | | | | ^17^ | |
| Ansul | | | 500 | | | | 930 | | | | | | | 12.0 | | | | | | | Cubic | | | | | | | | Sidewall | | | | | | | Argon | | | | | | | | 0.410 | | | | | | ^17^ | |
|  |  |  | 1,000 | | | | 930 | | | | | | | 4.50 | | | | | | | Cylindrical | | | | | | | | Base | | | | | | | Argon | | | | | | | | 2.17 | | | | | | ^17^ | |
| PFAS | | | ν  (kHz) | | | | C_0_  (mg L^-1^) | | | | | | | PD  (W L^-1^) | | | | | | | Reactor volume (L) | | | | | | | | Initial pH | | | | | | | Dissolved gas | | | | | | | | 0^th^ order rate constant  (nM min^-1^) | | | | | | Ref | |
|  |  |  |  |  |  |  |  |  |  |  |  |  |  |  |  |  |  |  |  |  |  |  |  |  |  |  |  |  |  |  |  |  |  |  |  |  |  |  |  |  |  |  |  | PFOA | | | | | PFOS |  |  |
| PFOA & PFOS | | | 575 | | | | 0.0476 & 0.0600 | | | | | | | 30.0 | | | | | | | 0.5 | | | | | | | | 6.02 | | | | | | | Air | | | | | | | | 0.570 | | | | | 0.280 | ^30^ | |
|  |  |  | 860 | | | | 0.0476 & 0.0600 | | | | | | | 50.0 | | | | | | | 0.5 | | | | | | | | 6.02 | | | | | | | Air | | | | | | | | 0.430 | | | | | 0.240 | ^30^ | |
|  |  |  | 1,140 | | | | 0.0476 & 0.0600 | | | | | | | 62.8 | | | | | | | 0.5 | | | | | | | | 6.02 | | | | | | | Air | | | | | | | | 0.440 | | | | | 0.360 | ^30^ | |
| PFAS | | | ν  (kHz) | | | | C_0_  (mg L^-1^) | | | | | | | PD  (W L^-1^) | | | | | | | Reactor volume (L) | | | | | | | | Initial pH | | | | | | | Dissolved gas | | | | | | | | Pseudo-1^st^ order rate constant (x10^-3^ min^-1^) | | | | | | Ref | |
|  |  |  |  |  |  |  |  |  |  |  |  |  |  |  |  |  |  |  |  |  |  |  |  |  |  |  |  |  |  |  |  |  |  |  |  |  |  |  |  |  |  |  |  | PFOA | | | | | PFOS |  |  |
| PFOA & PFOS | | | 575 | | | | 0.0422 & 0.0565 | | | | | | | 77.0 | | | | | | | 0.2 | | | | | | | | 6.02 | | | | | | | Air | | | | | | | | 23.5 | | | | | 6.00 | ^30^ | |
|  |  |  | 860 | | | | 0.0422 & 0.0565 | | | | | | | 113 | | | | | | | 0.2 | | | | | | | | 6.02 | | | | | | | Air | | | | | | | | 23.0 | | | | | 7.00 | ^30^ | |
|  |  |  | 1,140 | | | | 0.0422 & 0.0565 | | | | | | | 148 | | | | | | | 0.2 | | | | | | | | 6.02 | | | | | | | Air | | | | | | | | 22.5 | | | | | 6.50 | ^30^ | |
| PFAS | | | Ultrasonic Parameters | | | | | | | | | | | | | | | | | | | | | | | | | | | | | | PFAS Rate constant | | | | | | | | | | 0^th^ order rate constant | | | | | | | Ref | |
|  |  |  | ν  (kHz) | | | | | | | C_0_  (mg L^-1^) | | | | | | | | PD  (W L^-1^) | | | | | | | Dissolved gas | | | | | | | | Pseudo-1^st^ order  (10^-3^ min^-1^) | | | | | | | | | | F^-^  (µM min^-1^) | | | | SO_4_^2-^  (µM min^-1^) | | |  |  |
| PFOA & PFOS | | | 202 | | | | | | | (4.11 & 5.00) x10^-5^ | | | | | | | | 250 | | | | | | | Ar | | | | | | | | 0.020 & 0.010 | | | | | | | | | | - | | | | - | | | ^13^ | |
|  |  |  | 20 + 202 | | | | | | | (4.11 & 5.00) x10^-5^ | | | | | | | | 250 | | | | | | | Ar | | | | | | | | 0.027 & 0.013 | | | | | | | | | | - | | | | - | | | ^13^ | |
|  |  |  | 358 | | | | | | | (4.11 & 5.00) x10^-5^ | | | | | | | | 333 | | | | | | | Ar | | | | | | | | 0.057 & 0.040 | | | | | | | | | | - | | | | - | | | ^13^ | |
|  |  |  | 610 | | | | | | | (4.11 & 5.00) x10^-5^ | | | | | | | | 250 | | | | | | | Ar | | | | | | | | 0.034 & 0.020 | | | | | | | | | | - | | | | - | | | ^13^ | |
|  |  |  | 610 | | | | | | | (4.11 & 5.00) x10^-5^ | | | | | | | | 333 | | | | | | | Ar | | | | | | | | 0.043 & 0.029 | | | | | | | | | | - | | | | - | | | ^13^ | |
|  |  |  | 20 + 610 | | | | | | | (4.11 & 5.00) x10^-5^ | | | | | | | | 250 | | | | | | | Ar | | | | | | | | 0.037 & 0.021 | | | | | | | | | | - | | | | - | | | ^13^ | |
| PFOS | | | 9x 1,000 | | | | | | | 1,300 | | | | | | | | 109 | | | | | | | Ar | | | | | | | | - | | | | | | | | | | 0.30 | | | | 0.19 | | | ^20^ | |
|  |  |  | 3x 500 + 9x 1,000 | | | | | | | 1,300 | | | | | | | | 132 | | | | | | | Ar | | | | | | | | - | | | | | | | | | | 0.32 | | | | 0.32 | | | ^20^ | |
| AFFF brand | | | Ultrasonic Parameters | | | | | | | | | | | | | | | | | | | | | | | | | | | | | | PFAS Rate constant | | | | | | | | | | 0^th^ order rate constant | | | | | | | Ref | |
|  |  |  | ν  (kHz) | | | | | | | AFFF dilution ratio | | | | | | | | PD  (W L^-1^) | | | | | | | Dissolved gas | | | | | | | | Pseudo-1^st^ order  (10^-3^ min^-1^) | | | | | | | | | | F^-^  (µM min^-1^) | | | | SO_4_^2-^  (µM min^-1^) | | |  |  |
| Ansul | | | 9x 1,000 | | | | | | | 25 | | | | | | | | 109 | | | | | | | Ar | | | | | | | | - | | | | | | | | | | 0.0441 | | | | 0.223 | | | ^21^ | |
|  |  |  | 3x 500 + 9x 1,000 | | | | | | | 25 | | | | | | | | 132 | | | | | | | Ar | | | | | | | | - | | | | | | | | | | 0.0751 | | | | 0.163 | | | ^21^ | |
| 3M | | | 9x 1,000 | | | | | | | 25 | | | | | | | | 109 | | | | | | | Ar | | | | | | | | - | | | | | | | | | | 0.183 | | | | 0.257 | | | ^21^ | |
|  |  |  | 3x 500 + 9x 1,000 | | | | | | | 25 | | | | | | | | 132 | | | | | | | Ar | | | | | | | | - | | | | | | | | | | 0.288 | | | | 0.148 | | | ^21^ | |
| PFAS | Ultrasonic Parameters | | | | | | | | | | | | | | | | | | | PFAS pseudo-1^st^ order rate constant | | | | | | | | | | | | | | | | | | | | | | | | | | | | | | | Ref |
|  | C_0_  (mg L^-1^) | | | | | Dissolved gas | | | | | Volume  (L) | | | | PD  (W L^-1^) | | | | | ν (kHz) | | | | | | | | | | | | | | | | | | | | | | | | | | | | | | |  |
|  |  |  |  |  |  |  |  |  |  |  |  |  |  |  |  |  |  |  |  | 20 | | | | | | 40 | | | | | 202 | | | | | | 358 | | | | | 575# | | | | | | 610 | | |  |
| PFOA | 0.035 | | | | | Air | | | | | 0.5 | | | | 30.0 | | | | | - | | | | | | - | | | | | - | | | | | | - | | | | | 0.0025 | | | | | | - | | | ^31^ |
|  | 0.040 | | | | | Air | | | | | 0.3 | | | | 52.0 | | | | | - | | | | | | - | | | | | - | | | | | | - | | | | | 0.0030 | | | | | | - | | | ^31^ |
|  | 54.7 | | | | | - | | | | | 0.5 | | | | 60.0 | | | | | - | | | | | | 0.0025 | | | | | - | | | | | | - | | | | | - | | | | | | - | | | ^22^ |
|  | 0.043 | | | | | Air | | | | | 0.2 | | | | 77.0 | | | | | - | | | | | | - | | | | | - | | | | | | - | | | | | 0.0100 | | | | | | - | | | ^31^ |
|  | 0.099 | | | | | Ar | | | | | 0.6 | | | | 83.0 | | | | | - | | | | | | - | | | | | - | | | | | | 0.0063 | | | | | - | | | | | | 0.0080 | | | ^13^ |
|  | 0.042 | | | | | Air | | | | | 0.1 | | | | 147 | | | | | - | | | | | | - | | | | | - | | | | | | - | | | | | 0.024 | | | | | | - | | | ^31^ |
|  | 0.099 | | | | | Ar | | | | | 0.6 | | | | 166 | | | | | - | | | | | | - | | | | | - | | | | | | 0.0220 | | | | | - | | | | | | 0.0230 | | | ^13^ |
|  | 54.7 | | | | | - | | | | | 0.5 | | | | 180 | | | | | - | | | | | | 0.013 | | | | | - | | | | | | - | | | | | - | | | | | | - | | | ^22^ |
|  | 0.099 | | | | | Ar | | | | | 0.6 | | | | 250 | | | | | - | | | | | | - | | | | | - | | | | | | 0.0480 | | | | | - | | | | | | 0.0340 | | | ^13^ |
|  | 0.040 | | | | | Air | | | | | 0.05 | | | | 262 | | | | | - | | | | | | - | | | | | - | | | | | | - | | | | | 0.086 | | | | | | - | | | ^31^ |
|  | 0.099 | | | | | Ar | | | | | 0.6 | | | | 330 | | | | | - | | | | | | - | | | | | - | | | | | | 0.0570 | | | | | - | | | | | | 0.0430 | | | ^13^ |
|  | 7.5 x10^-5^ | | | | | - | | | | | 0.1 | | | | 375 | | | | | 0.075 | | | | | | - | | | | | - | | | | | | - | | | | | - | | | | | | - | | | ^24^ |
|  | 49.7 | | | | | - | | | | | 0.3 | | | | 500 | | | | | - | | | | | | 0.0313 | | | | | - | | | | | | - | | | | | - | | | | | | - | | | ^14^ |
| PFOS | 0.051 | | | | | Air | | | | | 0.5 | | | | 30.0 | | | | | - | | | | | | - | | | | | - | | | | | | - | | | | | 0.0020 | | | | | | - | | | ^31^ |
|  | 0.055 | | | | | Air | | | | | 0.3 | | | | 52.0 | | | | | - | | | | | | - | | | | | - | | | | | | - | | | | | 0.0025 | | | | | | - | | | ^31^ |
|  | 0.060 | | | | | Air | | | | | 0.2 | | | | 77.0 | | | | | - | | | | | | - | | | | | - | | | | | | - | | | | | 0.0050 | | | | | | - | | | ^31^ |
|  | 0.100 | | | | | Ar | | | | | 0.6 | | | | 83.0 | | | | | - | | | | | | - | | | | | - | | | | | | 0.0070 | | | | | - | | | | | | 0.0050 | | | ^13^ |
|  | 0.059 | | | | | Air | | | | | 0.2 | | | | 147 | | | | | - | | | | | | - | | | | | - | | | | | | - | | | | | 0.037 | | | | | | - | | | ^31^ |
|  | 0.100 | | | | | Ar | | | | | 0.6 | | | | 166 | | | | | - | | | | | | - | | | | | - | | | | | | 0.0170 | | | | | - | | | | | | 0.0180 | | | ^13^ |
|  | 0.100 | | | | | Ar | | | | | 0.6 | | | | 250 | | | | | - | | | | | | - | | | | | - | | | | | | 0.0280 | | | | | - | | | | | | 0.0220 | | | ^13^ |
|  | 0.057 | | | | | Air | | | | | 0.2 | | | | 262 | | | | | - | | | | | | - | | | | | - | | | | | | - | | | | | 0.068 | | | | | | - | | | ^31^ |
|  | 0.100 | | | | | Ar | | | | | 0.6 | | | | 330 | | | | | - | | | | | | - | | | | | - | | | | | | 0.0400 | | | | | - | | | | | | 0.0290 | | | ^13^ |
|  | 5.5 x10^-5^ | | | | | - | | | | | 0.1 | | | | 375 | | | | | 0.068 | | | | | | - | | | | | - | | | | | | - | | | | | - | | | | | | - | | | ^24^ |
| PFHxA | 0.100 | | | | | Ar | | | | | 0.6 | | | | 83.0 | | | | | - | | | | | | - | | | | | 0.0070 | | | | | | - | | | | | - | | | | | | 0.0100 | | | ^13^ |
|  | 0.100 | | | | | Ar | | | | | 0.6 | | | | 166 | | | | | - | | | | | | - | | | | | 0.0160 | | | | | | - | | | | | - | | | | | | 0.0210 | | | ^13^ |
|  | 0.100 | | | | | Ar | | | | | 0.6 | | | | 250 | | | | | - | | | | | | - | | | | | 0.0190 | | | | | | - | | | | | - | | | | | | 0.0360 | | | ^13^ |
|  | 0.100 | | | | | Ar | | | | | 0.6 | | | | 330 | | | | | - | | | | | | - | | | | | 0.0250 | | | | | | - | | | | | - | | | | | | 0.0340 | | | ^13^ |
| PFHxS | 0.092 | | | | | Ar | | | | | 0.6 | | | | 83.0 | | | | | - | | | | | | - | | | | | 0.0050 | | | | | | - | | | | | - | | | | | | 0.0070 | | | ^13^ |
|  | 0.092 | | | | | Ar | | | | | 0.6 | | | | 166 | | | | | - | | | | | | - | | | | | 0.0120 | | | | | | - | | | | | - | | | | | | 0.0140 | | | ^13^ |
|  | 0.092 | | | | | Ar | | | | | 0.6 | | | | 250 | | | | | - | | | | | | - | | | | | 0.0120 | | | | | | - | | | | | - | | | | | | 0.0220 | | | ^13^ |
|  | 0.092 | | | | | Ar | | | | | 0.6 | | | | 330 | | | | | - | | | | | | - | | | | | 0.0160 | | | | | | - | | | | | - | | | | | | 0.0270 | | | ^13^ |
| PFBA | 0.100 | | | | | Ar | | | | | 0.6 | | | | 83.0 | | | | | - | | | | | | - | | | | | 0.0044 | | | | | | - | | | | | - | | | | | | 0.0037 | | | ^13^ |
|  | 0.100 | | | | | Ar | | | | | 0.6 | | | | 166 | | | | | - | | | | | | - | | | | | 0.0064 | | | | | | - | | | | | - | | | | | | 0.0061 | | | ^13^ |
|  | 0.100 | | | | | Ar | | | | | 0.6 | | | | 250 | | | | | - | | | | | | - | | | | | 0.0072 | | | | | | - | | | | | - | | | | | | 0.0170 | | | ^13^ |
|  | 0.100 | | | | | Ar | | | | | 0.6 | | | | 330 | | | | | - | | | | | | - | | | | | 0.0065 | | | | | | - | | | | | - | | | | | | 0.0210 | | | ^13^ |
| PFBS | 0.090 | | | | | Ar | | | | | 0.6 | | | | 83.0 | | | | | - | | | | | | - | | | | | 0.0048 | | | | | | - | | | | | - | | | | | | 0.0040 | | | ^13^ |
|  | 0.090 | | | | | Ar | | | | | 0.6 | | | | 166 | | | | | - | | | | | | - | | | | | 0.0090 | | | | | | - | | | | | - | | | | | | 0.0060 | | | ^13^ |
|  | 0.090 | | | | | Ar | | | | | 0.6 | | | | 250 | | | | | - | | | | | | - | | | | | 0.0130 | | | | | | - | | | | | - | | | | | | 0.0170 | | | ^13^ |
|  | 0.090 | | | | | Ar | | | | | 0.6 | | | | 330 | | | | | - | | | | | | - | | | | | 0.0130 | | | | | | - | | | | | - | | | | | | 0.0210 | | | ^13^ |
| PFAS | Ultrasonic Parameters | | | | | | | | | | | | | | | | | | | | | | | | | | | | | | | PFAS pseudo-1^st^ order rate constant | | | | | | | | | | | | | | | | | | Ref | |
|  | C_0_ (mg L^-1^) | | | | | | | | ν (kHz) | | | | | | | Volume (L) | | | | | | | PD (W L^-1^) | | | | | | | | | Dissolved gas (polytropic index) | | | | | | | | | | | | | | | | | |  |  |
|  |  |  |  |  |  |  |  |  |  |  |  |  |  |  |  |  |  |  |  |  |  |  |  |  |  |  |  |  |  |  |  | O_2_ (1.397) | | | | | | Air (1.400) | | | | | N_2_ (1.403) | | | | Ar (1.670) | | |  |  |
| PFOA | 54.7 + (895 MnO_4_^-^) | | | | | | | | 40 | | | | | | | 0.5 | | | | | | | 180 | | | | | | | | | 0.0095 | | | | | | 0.013 | | | | | - | | | | 0.014 | | | ^22^ | |
|  | 70.4 + (895 IO_4_^-^) | | | | | | | | 40 | | | | | | | 0.3 | | | | | | | 500 | | | | | | | | | 0.0037 | | | | | | 0.0053 | | | | | 0.0092 | | | | - | | | ^16^ | |
| Substance | | Ultrasonic Parameters | | | | | | | | | | | | | | | | | | | | | | Structural parameters | | | | | | | | | | | | | | | | | Rate constant | | | | | | | | | Ref | |
|  |  | C_0_  (mg L^-1^) | | | | | | ν  (kHz) | | | | V  (L) | | | | | | | PD  (W L^-1^) | | | | | Acid head group | | | | | | Perfluoro chain length | | | | | Carbon chain length | | | | | | Pseudo-1^st^ order PFAS  (10^-3^ min^-1^) | | | | | Zero order fluoride  (μM min^-1^) | | | |  |  |
| PFEES | | 59.7 | | | | | | 500 | | | | 12.2 | | | | | | | 8 (W cm^-2^) | | | | | Sulphonic | | | | | | 4 | | | | | 4 | | | | | | - | | | | | 3.9 | | | | ^18^ | |
| PFBS | | 45.6 | | | | | | 500 | | | | 12.2 | | | | | | | 8 (W cm^-2^) | | | | | Sulphonic | | | | | | 4 | | | | | 4 | | | | | | - | | | | | 1.8 | | | | ^18^ | |
| PFHxS | | 56.1 | | | | | | 500 | | | | 12.2 | | | | | | | 8 (W cm^-2^) | | | | | Sulphonic | | | | | | 6 | | | | | 6 | | | | | | - | | | | | 2.6 | | | | ^18^ | |
| 6:2 FTS | | 56.0 | | | | | | 500 | | | | 12.2 | | | | | | | 8 (W cm^-2^) | | | | | Sulphonic | | | | | | 6 | | | | | 8 | | | | | | - | | | | | 1.5 | | | | ^18^ | |
| PFOS | | 50.0 | | | | | | 500 | | | | 12.2 | | | | | | | 8 (W cm^-2^) | | | | | Sulphonic | | | | | | 8 | | | | | 8 | | | | | | - | | | | | 3.5 | | | | ^18^ | |
| PFOS | | 10.0 | | | | | | 200 | | | | 0.06 | | | | | | | 3,333 | | | | | Sulphonic | | | | | | 8 | | | | | 8 | | | | | | 16 | | | | | - | | | | ^1^ | |
| OS^@^ | | 10.0 | | | | | | 200 | | | | 0.06 | | | | | | | 3,333 | | | | | Sulphonic | | | | | | 0 | | | | | 8 | | | | | | 220 | | | | | - | | | | ^1^ | |
| PFPrA | | 55.4 | | | | | | 500 | | | | 12.2 | | | | | | | 8 (W cm^-2^) | | | | | Carboxylic | | | | | | 2 | | | | | 3 | | | | | | - | | | | | 1.6 | | | | ^18^ | |
| PFPeA | | 49.7 | | | | | | 500 | | | | 12.2 | | | | | | | 8 (W cm^-2^) | | | | | Carboxylic | | | | | | 4 | | | | | 5 | | | | | | - | | | | | 2.5 | | | | ^18^ | |
| PFHxA | | 48.2 | | | | | | 500 | | | | 12.2 | | | | | | | 8 (W cm^-2^) | | | | | Carboxylic | | | | | | 5 | | | | | 6 | | | | | | - | | | | | 3.5 | | | | ^18^ | |
| PFOA | | 46.8 | | | | | | 500 | | | | 12.2 | | | | | | | 8 (W cm^-2^) | | | | | Carboxylic | | | | | | 7 | | | | | 8 | | | | | | - | | | | | 3.7 | | | | ^18^ | |
| PFOA | | 10.0 | | | | | | 200 | | | | 0.06 | | | | | | | 3,333 | | | | | Carboxylic | | | | | | 7 | | | | | 8 | | | | | | 32 | | | | | - | | | | ^1^ | |
| OA^@^ | | 10.0 | | | | | | 200 | | | | 0.06 | | | | | | | 3,333 | | | | | Carboxylic | | | | | | 0 | | | | | 8 | | | | | | 220 | | | | | - | | | | ^1^ | |
| PFOA & PFOS | | (4.11 & 5.00) x10^-5^ | | | | | | 358 | | | | 0.60 | | | | | | | 333 | | | | | Sulphonic & carboxylic | | | | | | 7 & 8 | | | | | 8 & 8 | | | | | | 0.057 & 0.040 | | | | | - | | | | ^13^ | |
| PFHA & PFHS | | 0.117 & 0.092 | | | | | | 358 | | | | 0.60 | | | | | | | 250 | | | | | Sulphonic & carboxylic | | | | | | 5 & 6 | | | | | 6 & 6 | | | | | | 0.039 & 0.030 | | | | | - | | | | ^6^ | |
| PFBA & PFBS | | 0.101 & 0.090 | | | | | | 619 | | | | 0.60 | | | | | | | 333 | | | | | Sulphonic & carboxylic | | | | | | 3 & 4 | | | | | 4 & 4 | | | | | | 0.021 & 0.021 | | | | | - | | | | ^6^ | |
| PFAS | | | | | Ultrasonic Parameters | | | | | | | | | | | | | | | | | | | | | | | | | | | | | | | | | | Kinetic transition concentration (μM) | | | | | | | | | | | Ref | |
|  |  |  |  |  | Frequency (kHz) | | | | | | | | | | | | Power Density (W L^-1^) | | | | | | | | | | | Dissolved gas choice (-) | | | | | | | | | | |  |  |  |  |  |  |  |  |  |  |  |  |  |
| Na-PFOS | | | | | 354 | | | | | | | | | | | | 250 | | | | | | | | | | | Argon | | | | | | | | | | | 39.0 | | | | | | | | | | | ^3^ | |
| NH_4_-PFOA | | | | | 354 | | | | | | | | | | | | 250 | | | | | | | | | | | Argon | | | | | | | | | | | 30.5 | | | | | | | | | | | ^3^ | |
| PFOS in FC-600 AFFF | | | | | 505 | | | | | | | | | | | | 188 | | | | | | | | | | | Argon | | | | | | | | | | | 14.6 - 29.2* | | | | | | | | | | | ^7^ | |
| PFOA & K-PFOS | | | | | 575 | | | | | | | | | | | | 77^+^ | | | | | | | | | | | Air | | | | | | | | | | | 23.6  29.5 | | | | | | | | | | | ^23^ | |
| PFOA & K-PFOS | | | | | 20 | | | | | | | | | | | | 1,500 – 3,750 | | | | | | | | | | | Air^^^ | | | | | | | | | | | 0.0004  0.0006 | | | | | | | | | | | ^24^ | |
| K-PFOS | | | | | 400-1,000 | | | | | | | | | | | | 200 | | | | | | | | | | | Air | | | | | | | | | | | 2.30 – 2.90 | | | | | | | | | | | ^27^ | |

| PFAS | | Oxidant | | | | | Radicals formed^ | | | ν (kHz) | | | | | Power density  (W L^-1^) | | Degradation rate  (min^-1^) | | Ref |
| --- | --- | --- | --- | --- | --- | --- | --- | --- | --- | --- | --- | --- | --- | --- | --- | --- | --- | --- | --- |
| PFOA | | KMnO_4_ | | | | | $OH\bullet$ | | | 40 | | | | | 180 | | 0.015 | | ^22^ |
| PFOA  PFOS  6:2 FTS | | Na_2_S_2_O_8_ | | | | | $OH\bullet$ &${SO}_{4}^{-\bullet}$ | | | 20 & 43 | | | | | 550 & 250 | | 0.370 | | ^25^ |
| PFECs | | K_2_S_2_O_8_ | | | | | ${SO}_{4}^{-\bullet}$^-^ | | | 28 | | | | | 33.3 | | 0.320 | | ^9^ |
| PFOA | | TiO_2_ + 254 nm UV light | | | | | $OH\bullet$ & $O_{2}^{-\bullet}$ | | | 40 | | | | | 167 | | 0.350 | | ^5^ |
| PFOA | | KIO_4_ | | | | | ${IO}_{3}\bullet$ | | | 40 | | | | | 500 | | 0.00022 – 0.0022 | | ^16^ |
| PFOA | | NaHCO_3_ | | | | | $CO_{3}^{\bullet-}$ | | | 40 | | | | | 150 | | 0.0022 | | ^11^ |
| NH_4_-PFOA | | K_2_S_2_O_8_ | | | | | ${SO}_{4}^{-\bullet}$ | | | 20 | | | | | 3,000 | | - | | ^10^ |
| PFOA | | Na_2_SO_4_ | | | | | ${SO}_{4}^{-\bullet}$ | | | 40 | | | | | 500 | | - | | ^14^ |
| PFAS | | Ultrasonic Parameters | | | | | | | | | | | | | | | | | - Ref |
|  |  | ν  (kHz) | | | C_0,PFAS_  (μM) | | | PD  (W L^-1^) | | | | Degradation rate (min^-1^) | | pH_0_ | | Oxidant | | C_0, Oxidant_ (mM) |  |
| PFOA | | 40 | | | 120 | | | 500 | | | | Not given | | 4.3 | | Na_2_SO_4_ | | 0 & 46 | ^14^_ |
| PFOA | | 40 | | | 132 | | | 180 | | | | 1.5 x10^-2^ | | 4.0 | | KMnO_4_ | | 6.0 | ^22^_ |
| PFOA & KPFOS | | 20 | | | 2 x10^-4^ | | | 3,750 | | | | 7.5 x10^-2^  6.8 x10^-2^ | | 2.0 | | None | | N/A | ^24^ |
| PFOA & PFOS | | 575 | | | 0.102 & 0.113 | | | 77 | | | | 2.4 x10^-2^  7.0 x10^-3^ | | Not given | | None | | N/A | ^30^ |
| PFAS | Ultrasonic Parameters | | | | | | | | | | | | | | | | | | Ref |
|  | C_0,PFAS_  (μM) | | ν  (kHz) | PD  (W L^-1^) | | Temperature (°C) | | | Dissolved gas | | Oxidant/  Reagents | | C_0, Oxidant_ (mM) | | | | | |  |
| PFOA | 120 | | 40 | 166.7 + 5.3 UV | | 25 | | | O_2_ | | RdH or Sol-gel TiO_2_ | | 8.26 | | | | | | _^5^ |
| PFOA + PFOS | 0.200 + 0.240 | | 612 | 250 | | 10 | | | Argon | | None | | N/A | | | | | | ^8^ |
| NH_4_PFOA | 46.4 | | 20 | 6,000 | | 25 | | | Air | | K_2_S_2_O_8_ | | 10 | | | | | | ^10^ |
| PFOA | 120.75 | | 40 | 150 | | 25 | | | Air^ | | Na_2_CO­_3_ | | - | | | | | | ^11^ |
| PFOA | 120 | | 40 | 500 | | 25 | | | Air^ | | Na_2_SO_4_ | | 46 | | | | | | ^14^ |
| PFOA | 120 | | 40 | 500 | | 25 | | | Air^ | | CTAB | | 0.12 | | | | | | ^15^ |
| PFOA | 170.1 | | 40 | 500 | | 25 | | | Air^ | | KIO_4_ | | 4.5 | | | | | | ^16^ |
| PFOS | 2,600 | | 1 MHz + 500 kHz | 131.9 | | 25 | | | Air^ | | None | | N/A | | | | | | ^20^ |
| PFOA | 132 | | 40 | 180 | | 30 | | | Air^ | | KMnO_4_ | | 10 | | | | | | ^22^ |
| Various in AFFF | 1.6 | | 1 MHz + 500 kHz | 131.9 | | - | | | Air^ | | None | | N/A | | | | | | ^21^ |
| PFOA + PFOS | 2 x 10^-4^ | | 20 | 3,750 | | 20 | | | Air^ | | None | | N/A | | | | | | ^24^ |
| PFOA | 10 | | 20 | 550 | | - | | | Air^ | | Na_2_SO_4_ | | 7.04 | | | | | | ^25^ |
| PFOA + PFOS | 0.102 + 0.113 | | 575 | 77 | | 21.3 | | | Air | | Nine | | N/A | | | | | | ^30^ |
| PFOA + PFOS | 0.102 + 0.113 | | 575 | 77 | | 21.3 | | | Argon sparging | | Nine | | N/A | | | | | | ^30^ |

| AFFF brand | Dilution range tested | Frequency (kHz) | Optimum dilution factor | | | | Ref |
| --- | --- | --- | --- | --- | --- | --- | --- |
|  |  |  | (PFAS removal) | (TOC removal) | (F^-^ release) | (SO_4_^2-^ release) |  |
| FC-600 | 250 – 50,000 $\times$ | 505 | 5,000 $\times$ | - | - | - | ^7^ |
| 3M | 200 – 929.4 $\times$ | 1,000 | - | 200 $\times$ | 200 $\times$ | - | ^17^ |
| Ansul | 200 – 929.4 $\times$ | 1,000 | - | 200 $\times$ | 200 $\times$ | - | ^17^ |
| 3M | 10 – 500 $\times$ | 500 + 1,000 | - | 500 $\times$ | 10 $\times$ | 25 $\times$ | ^21^ |
| Ansul | 25 – 900 $\times$ | 500 + 1,000 | - | 500 – 900 $\times$ | 25 – 100 $\times$ | 100 $\times$ | ^21^~ |

| Technology | Treatment time (h) | Initial Concentration  (mg L^−1^) | Efficiency  (g kW^−1^ h^−1^) | Short chain formation | Ref |
| --- | --- | --- | --- | --- | --- |
| Electrochemical | 4 | 0.0152 | 0.00033 | Up to 50% | ^34^ |
| Photochemical | 240 | 20.0 | 0.00133 | Observed, significant quantity indicated (71% F− release) | ^35^ |
| Photochemical, ferric ion | 60 | 10.0 | 0.00290 | ~14% of initial mass | ^36^ |
| Electrochemical | 2 | 8.00 | 0.00566 | Not discussed | ^37^ |
| Sonication, 618 kHz | 3 | 5.00 | 0.00801 | Almost none implied (~100% F− release) | ^2^ |
| Photochemical, persulfate | 2 | 10.0 | 0.00900 | Observed, significant quantity indicated (76% F− release) | ^38^ |
| Photochemical, propanol | 24 | 20.0 | 0.01520 | Not discussed | ^35^ |
| Sonication, 400 kHz | 4 | 9.42 | 0.01550 | 1% of initial mass | ^27^ |
| Plasma | 4 | 50.0 | 0.02600 | Not discussed, none implied | ^39^ |
| Sonication, 400 kHz | 2 | 9.42 | 0.02610 | 13% of initial mass | ^27^ |
| Sonication, 358 kHz | 3 | 59.5 | 0.04170 | Not discussed | ^3^ |
| Plasma | 0.5 | 0.0001 | 0.06900 | Observed, 5.65% of initial mass after 40 min) | ^40^ |
| Plasma | 1 | 100 | 0.62100 | Observed, significant quantity indicated (~30% F− release | ^41^ |
| *# Data estimate from graph*  **Data calculated based on 1 in 250-500 dilution of 7.3 mM PFOS in initial FC-600 AFFF*  *_ Mechanical stirring also applied*  *^+^Calorimetrically measured*  *^^^Assume since not stated or measured*  *~Optimum value changes over time*  OS^@^ - 1-Octane sulphonic acid  OA^@^ - n-Octanoic acid  *- Indicates data not reported or calculable*  *Data are shaded from white to dark green in order of increasing value in any column (or row in the case of dissolved gas effects)* | | | | | |

Table S4: m/z values of some PFEC ions and their breakdown products during sonolysis

| **Starting ion:** | | NDFTOTDA | PDFTODA | TDFTODA | UDFDOOA | UDFOHpA | NFDOHpA | NFOHxA | HpFOPA | PFOBA |
| --- | --- | --- | --- | --- | --- | --- | --- | --- | --- | --- |
| **m/z value:** | | 561 | 445 | 411 | 345 | 329 | 295 | 279 | 229 | 179 |
| **Breakdown products** | |  |  |  |  |  |  |  |  |  |
| Ion | m/z value: |  |  |  |  |  |  |  |  |  |
| NDFTOTDA | 561 |  |  |  |  |  |  | **Key** | **Meaning** |  |
| PDFTODA | 445 | ⏹ |  |  |  |  |  | ⏹ | Plausible |  |
| TDFTODA | 411 |  |  |  |  |  |  | ✔ | Observed |  |
| UDFDOOA | 345 |  |  |  |  |  |  |  |  |  |
| UDFOHpA | 329 | ⏹ | ⏹ |  |  |  |  |  |  |  |
| NFDOHpA | 295 |  |  | ⏹ |  |  |  |  |  |  |
| NFOHxA | 279 |  |  |  |  |  |  |  |  |  |
| HpFOPA | 229 |  |  |  | ✔ |  |  |  |  |  |
| PFOBA | 179 |  |  | ⏹ |  |  | ✔ |  |  |  |
|  | Reference: |  |  |  | ^9^ |  | ^9^ |  |  |  |

Table S5: List of perfluoroether carboxylate chemical structures, names and acronyms as decided based on structures given in ^9^

| **Structure** | **Name** | **Acronym** |
| --- | --- | --- |
| C_4_F_9_OC_2_F_4_OC_2_F_4_OCF_2_COOH | Nonadecafluoro-3,6,9-trioxatridecanoic acid | NDFTOTDA |
| C_4_F_9_OC_2_F_4_OCF_2_COOH | Pentadecafluoro-3,6-dioxadecanoic acid | PDFTODA |
| CF_3_OC_2_F_4_OC_2_F_4_OCF_2_COOH | Tridecafluoro-3,6,9-trioxadecanoic acid | TDFTODA |
| C_2_F_5_OC_2_F_4_OCF_2_COOH | Undecafluoro-3,6-dioxaoctanoic acid | UDFDOOA |
| C_4_F_9_OCF_2_COOH | Undecafluoro-3-oxaheptanoic acid | UDFOHpA |
| CF_3_OC_2_F_4_OCF_2_COOH | Nanofluoro-3,6-dioxaheptanoic acid | NFDOHpA |
| CF_3_OC_3_F_6_COOH | Nanofluoro-5-oxahexanoic acid | NFOHxA |
| C_2_F_5_OCF_2_COOH | Heptafluoro-3-pentanoic acid | HpFOPA |
| CF_3_OCF_2_COOH | Pentafluoro-3-Butanoic acid | PFOBA |

# Supplementary Information References

1. Moriwaki H, Takagi Y, Tanaka M, Tsuruho K, Okitsu K, Maeda Y. Sonochemical decomposition of perfluorooctane sulfonate and perfluorooctanoic acid. *Environ Sci Technol*. 2005;39(9):3388-3392. doi:10.1021/es040342v

2. Vecitis CD, Park H, Cheng J, Mader BT, Hoffmann MR. Kinetics and Mechanism of the Sonolytic Conversion of the Aqueous Perfluorinated Surfactants, Perfiuorooctanoate (PFOA), and Perfluorooctane Sulfonate (PFOS) into Inorganic Products. *J Phys Chem A*. 2008;112(18):4261-4270. doi:10.1021/jp801081y

3. Vecitis CD, Park H, Cheng J, Mader BT, Hoffmann MR. Enhancement of perfluorooctanoate and perfluorooctanesulfonate activity at acoustic cavitation bubble interfaces. *J Phys Chem C*. 2008;112(43):16850-16857. doi:10.1021/jp804050p

4. Cheng J, Vecitis CD, Park H, Mader BT, Hoffmann MR. Sonochemical degradation of perfluorooctane sulfonate (PFOS) and perfluorooctanoate (PFOA) in landfill groundwater: Environmental matrix effects. *Environ Sci Technol*. 2008;42(21):8057-8063. doi:10.1021/es8013858

5. Panchangam SC, Lin AYC, Tsai JH, Lin CF. Sonication-assisted photocatalytic decomposition of perfluorooctanoic acid. *Chemosphere*. 2009;75(5):654-660. doi:10.1016/j.chemosphere.2008.12.065

6. Campbell TY, Vecitis CD, Mader BT, Hoffmann MR. Perfluorinated surfactant chain-length effects on sonochemical kinetics. *J Phys Chem A*. 2009;113(36):9834-9842. doi:10.1021/jp903003w

7. Vecitis CD, Wang YJ, Cheng J, Park H, Mader BT, Hoffmann MR. Sonochemical Degradation of Perfluorooctanesulfonate in Aqueous Film-Forming Foams. *Environ Sci Technol*. 2010;44(1):432-438. doi:Doi 10.1021/Es902444r

8. Jie Cheng, Chad D. Vecitis, Hyunwoong Park, Brian T. Mader MRH, Cheng JIE, Vecitis CD, et al. Sonochemical Degradation of Perfluorooctane Sulfonate (PFOS) and Perfluorooctanoate (PFOA) in Groundwater: Kinetic Effects of Matrix Inorganics. *Environ Sci Technol*. 2010;44(1):445–450. doi:10.1021/es902651g

9. Hori H, Nagano Y, Murayama M, Koike K, Kutsuna S. Efficient decomposition of perfluoroether carboxylic acids in water with a combination of persulfate oxidant and ultrasonic irradiation. *J Fluor Chem*. 2012;141:5-10. doi:10.1016/j.jfluchem.2012.05.012

10. Hao F, Guo W, Wang A, Leng Y, Li H. Intensification of sonochemical degradation of ammonium perfluorooctanoate by persulfate oxidant. *Ultrason Sonochem*. 2014;21(2):554-558. doi:10.1016/j.ultsonch.2013.09.016

11. Phan Thi L-A, Do H-T, Lo S-L. Enhancing decomposition rate of perfluorooctanoic acid by carbonate radical assisted sonochemical treatment. *Ultrason Sonochem*. 2014;21(5):1875-1880. doi:10.1016/J.ULTSONCH.2014.03.027

12. Rodriguez-Freire L, Balachandran R, Sierra-Alvarez R, Keswani M. Effect of sound frequency and initial concentration on the sonochemical degradation of perfluorooctane sulfonate (PFOS). *J Hazard Mater*. 2015;300:662-669. doi:10.1016/j.jhazmat.2015.07.077

13. Campbell T, Hoffmann MR. Sonochemical degradation of perfluorinated surfactants: Power and multiple frequency effects. *Sep Purif Technol*. 2015;156(April):1019-1027. doi:10.1016/j.seppur.2015.09.053

14. Lin JC, Lo SL, Hu CY, Lee YC, Kuo J. Enhanced sonochemical degradation of perfluorooctanoic acid by sulfate ions. *Ultrason Sonochem*. 2015;22:542-547. doi:10.1016/j.ultsonch.2014.06.006

15. Lin JC, Hu CY, Lo SL. Effect of surfactants on the degradation of perfluorooctanoic acid (PFOA) by ultrasonic (US) treatment. *Ultrason Sonochem*. 2016;28:130-135. doi:10.1016/j.ultsonch.2015.07.007

16. Lee YC, Chen MJ, Huang CP, Kuo J, Lo SL. Efficient sonochemical degradation of perfluorooctanoic acid using periodate. *Ultrason Sonochem*. 2016;31:499-505. doi:10.1016/j.ultsonch.2016.01.030

17. Rodriguez-Freire L, Abad-Fernández N, Sierra-Alvarez R, et al. Sonochemical degradation of perfluorinated chemicals in aqueous film-forming foams. *J Hazard Mater*. 2016;317:275-283. doi:10.1016/j.jhazmat.2016.05.078

18. Fernandez NA, Rodriguez-Freire L, Keswani M, Sierra-Alvarez R. Effect of chemical structure on the sonochemical degradation of perfluoroalkyl and polyfluoroalkyl substances (PFASs). *Environ Sci Water Res Technol*. 2016;2(6):975-983. doi:10.1039/c6ew00150e

19. Sekiguchi K, Kudo T, Sankoda K. Combined sonochemical and short-wavelength UV degradation of hydrophobic perfluorinated compounds. *Ultrason Sonochem*. 2017;39(January):87-92. doi:10.1016/j.ultsonch.2017.04.002

20. Gole VL, Fishgold A, Sierra-Alvarez R, Deymier P, Keswani M. Treatment of perfluorooctane sulfonic acid (PFOS) using a large-scale sonochemical reactor. *Sep Purif Technol*. 2018;194(November 2017):104-110. doi:10.1016/j.seppur.2017.11.009

21. Gole VL, Sierra-Alvarez R, Peng H, Giesy JP, Deymier P, Keswani M. Sono-chemical treatment of per- and poly-fluoroalkyl compounds in aqueous film-forming foams by use of a large-scale multi-transducer dual-frequency based acoustic reactor. *Ultrason Sonochem*. 2018;45(February):213-222. doi:10.1016/j.ultsonch.2018.02.014

22. Hu Y bo, Lo SL, Li YF, Lee YC, Chen MJ, Lin JC. Autocatalytic degradation of perfluorooctanoic acid in a permanganate-ultrasonic system. *Water Res*. 2018;140:148-157. doi:10.1016/j.watres.2018.04.044

23. Shende T, Andaluri G, Suri RPS. Kinetic model for sonolytic degradation of non-volatile surfactants: Perfluoroalkyl substances. *Ultrason Sonochem*. 2019;51:359-368. doi:10.1016/j.ultsonch.2018.08.028

24. Panda D, Sethu V, Manickam S. Kinetics and mechanism of low-frequency ultrasound driven elimination of trace level aqueous perfluorooctanesulfonic acid and perfluorooctanoic acid. *Chem Eng Process - Process Intensif*. 2019;142(May):107542. doi:10.1016/j.cep.2019.107542

25. Lei Y, Tian Y, Sobhani Z, Naidu R, Fang C. Synergistic degradation of PFAS in water and soil by dual-frequency ultrasonic activated persulfate. *Chem Eng J*. 2020;388(January):124215. doi:10.1016/j.cej.2020.124215

26. Cao H, Zhang W, Wang C, Liang Y. Sonochemical degradation of poly- and perfluoroalkyl substances – A review. *Ultrason Sonochem*. 2020;69(June). doi:10.1016/j.ultsonch.2020.105245

27. Wood RJ, Sidnell T, Ross I, et al. Ultrasonic degradation of perfluorooctane sulfonic acid (PFOS) correlated with sonochemical and sonoluminescence characterisation. *Ultrason Sonochem*. 2020;68(August 2019):105196. doi:10.1016/j.ultsonch.2020.105196

28. Laramay F, Crimi M. Theoretical evaluation of chemical and physical feasibility of an in situ ultrasonic reactor for remediation of groundwater contaminated with per- and polyfluoroalkyl substances. *Remediation*. 2020;31(1):45-58. doi:10.1002/rem.21666

29. Laramay F, Crimi M. A sustainability assessment of an in situ ultrasonic reactor for remediation of PFAS-contaminated groundwater. *Remediation*. 2020;31(1):59-72. doi:10.1002/rem.21667

30. Shende T, Andaluri G, Suri R, Gangadhar A, Suri R. Frequency-dependent sonochemical degradation of perfluoroalkyl substances. *Sep Purif Technol*. 2021;261(118250):118250. doi:10.1016/j.seppur.2020.118250

31. Shende T, Andaluri G, Suri R. Power density modulated ultrasonic degradation of perfluoroalkyl substances with and without sparging Argon. *Ultrason Sonochem*. 2021;76. doi:10.1016/j.ultsonch.2021.105639

32. Singh Kalra S, Cranmer B, Dooley G, et al. Sonolytic destruction of Per- and polyfluoroalkyl substances in groundwater, aqueous Film-Forming Foams, and investigation derived waste. *Chem Eng J*. 2021;425(March):131778. doi:10.1016/j.cej.2021.131778

33. Zhang W, Zhang Q, Liang Y. Ineffectiveness of ultrasound at low frequency for treating per- and polyfluoroalkyl substances in sewage sludge. *Chemosphere*. 2022;286(P2):131748. doi:10.1016/j.chemosphere.2021.131748

34. Uwayezu JN, Carabante I, Lejon T, et al. Electrochemical degradation of per- and poly-fluoroalkyl substances using boron-doped diamond electrodes. *J Environ Manage*. 2021;290(February):112573. doi:10.1016/j.jenvman.2021.112573

35. Yamamoto T, Noma Y, Sakai SI, Shibata Y. Photodegradation of perfluorooctane sulfonate by UV irradiation in water and alkaline 2-propanol. *Environ Sci Technol*. 2007;41(16):5660-5665. doi:10.1021/es0706504

36. Jin L, Zhang P, Shao T, Zhao S. Ferric ion mediated photodecomposition of aqueous perfluorooctane sulfonate (PFOS) under UV irradiation and its mechanism. *J Hazard Mater*. 2014;271:9-15. doi:10.1016/j.jhazmat.2014.01.061

37. Sukeesan S, Boontanon N, Boontanon SK. Improved electrical driving current of electrochemical treatment of Per- and Polyfluoroalkyl Substances (PFAS) in water using Boron-Doped Diamond anode. *Environ Technol Innov*. 2021;23:101655. doi:10.1016/j.eti.2021.101655

38. Park H, Vecitis CD, Cheng J, Dalleska NF, Mader BT, Hoffmann MR. Reductive degradation of perfluoroalkyl compounds with aquated electrons generated from iodide photolysis at 254 nm. *Photochem Photobiol Sci*. 2011;10(12):1945-1953. doi:10.1039/c1pp05270e

39. Yasuoka K, Sasaki K, Hayashi R. An energy-efficient process for decomposing perfluorooctanoic and perfluorooctane sulfonic acids using dc plasmas generated within gas bubbles. *Plasma Sources Sci Technol*. 2011;20(3). doi:10.1088/0963-0252/20/3/034009

40. Stratton GR, Dai F, Bellona CL, Holsen TM, Dickenson ERV, Mededovic Thagard S. Plasma-Based Water Treatment: Efficient Transformation of Perfluoroalkyl Substances in Prepared Solutions and Contaminated Groundwater. *Environ Sci Technol*. 2017;51(3):1643-1648. doi:10.1021/acs.est.6b04215

41. Lewis AJ, Joyce T, Hadaya M, et al. Rapid degradation of PFAS in aqueous solutions by reverse vortex flow gliding arc plasma. *Environ Sci Water Res Technol*. 2020;6(4):1044-1057. doi:10.1039/c9ew01050e
